# Supplementary material for: Computational perspectives revealed prospective vaccine candidates from five structural proteins of novel SARS corona virus 2019 (SARS-CoV-2)
Source: PeerJ. 2020 Sep 29;8:e9855. doi: 10.7717/peerj.9855 (PMC7531350; doi:10.7717/peerj.9855)
Supplement: Supplemental Information 1 [file peerj-08-9855-s001.pdf]

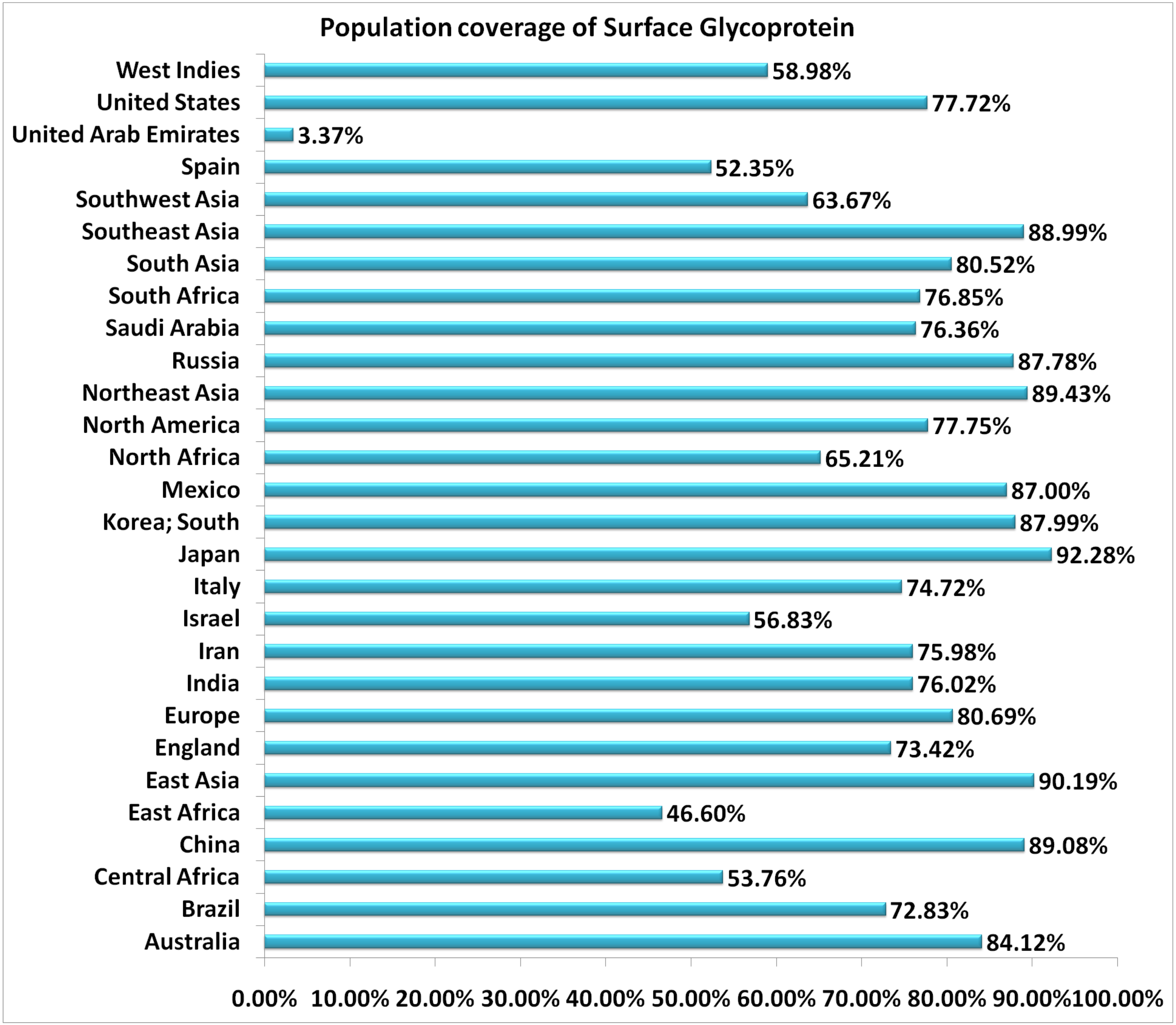
Population coverage of Surface Glycoprotein
West Indies 58.98%
United States 77.72%
United Arab Emirates 3.37%
Spain 52.35%
Southwest Asia 63.67%
Southeast Asia 88.99%
South Asia 80.52%
South Africa 76.85%
Saudi Arabia 76.36%
Russia 87.78%
Northeast Asia 89.43%
North America 77.75%
North Africa 65.21%
Mexico 87.00%
Korea; South 87.99%
Japan 92.28%
Italy 74.72%
Israel 56.83%
Iran 75.98%
India 76.02%
Europe 80.69%
England 73.42%
East Asia 90.19%
East Africa 46.60%
China 89.08%
Central Africa 53.76%
Brazil 72.83%
Australia 84.12%
0.00% 10.00% 20.00% 30.00% 40.00% 50.00% 60.00% 70.00% 80.00% 90.00% 100.00%

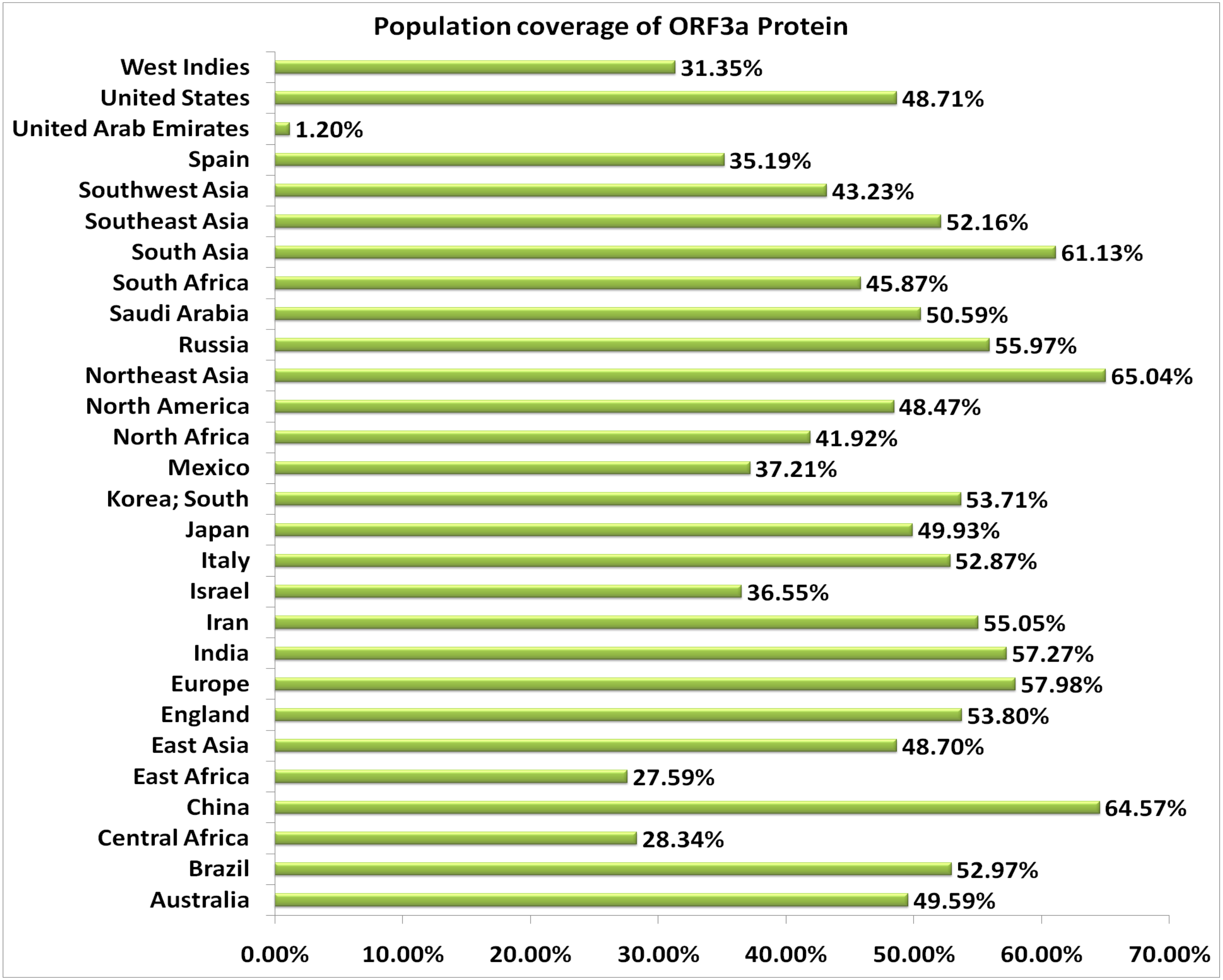

Population coverage of ORF3a Protein
West Indies
31.35%
United States
48.71%
United Arab Emirates
1.20%
Spain
35.19%
Southwest Asia
43.23%
Southeast Asia
52.16%
South Asia
61.13%
South Africa
45.87%
Saudi Arabia
50.59%
Russia
55.97%
Northeast Asia
65.04%
North America
48.47%
North Africa
41.92%
Mexico
37.21%
Korea; South
53.71%
Japan
49.93%
Italy
52.87%
Israel
36.55%
Iran
55.05%
India
57.27%
Europe
57.98%
England
53.80%
East Asia
48.70%
East Africa
27.59%
China
64.57%
Central Africa
28.34%
Brazil
52.97%
Australia
49.59%
0.00%
10.00%
20.00%
30.00%
40.00%
50.00%
60.00%
70.00%

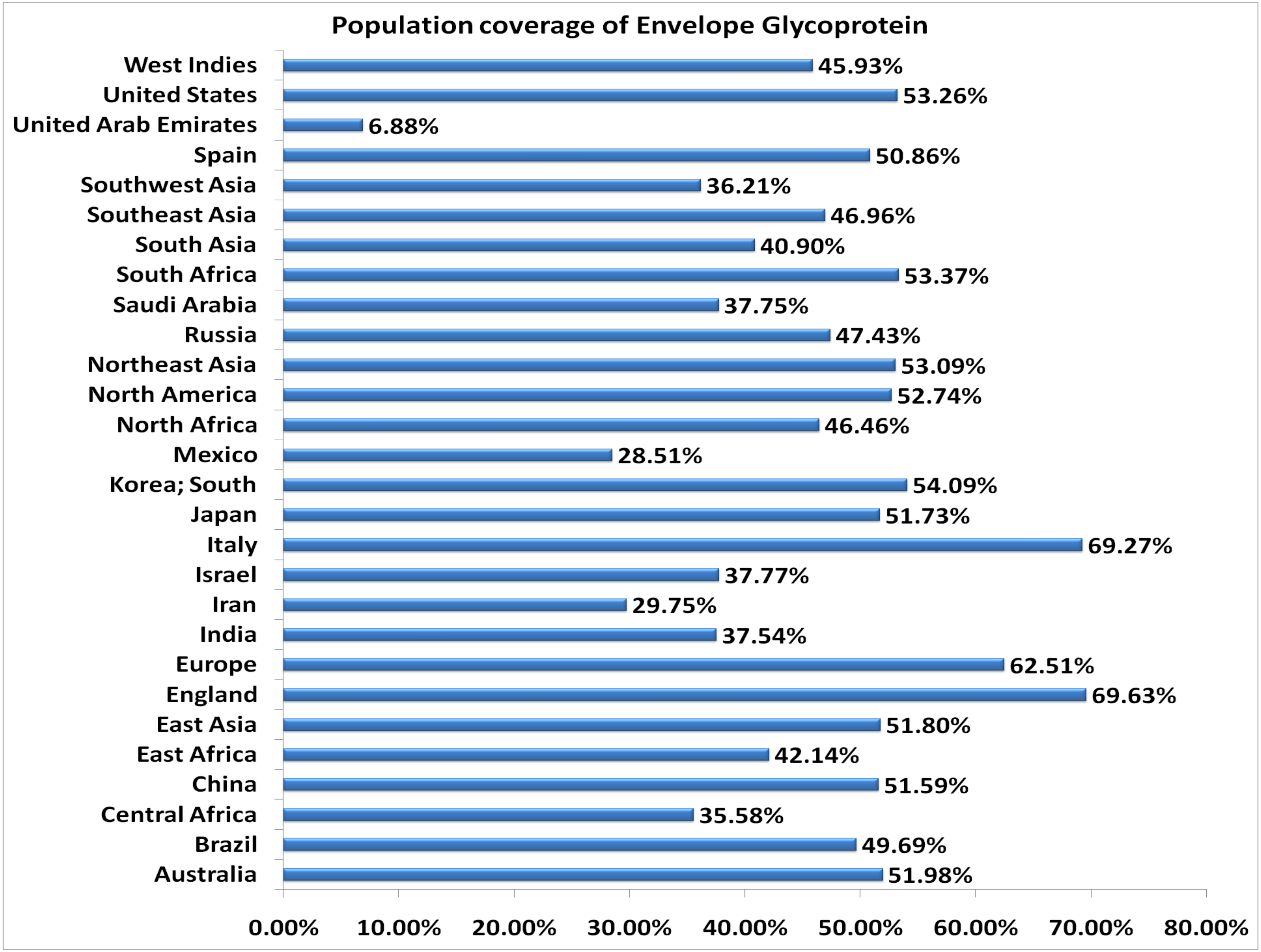
Population coverage of Envelope Glycoprotein
West Indies 45.93%
United States 53.26%
United Arab Emirates 6.88%
Spain 50.86%
Southwest Asia 36.21%
Southeast Asia 46.96%
South Asia 40.90%
South Africa 53.37%
Saudi Arabia 37.75%
Russia 47.43%
Northeast Asia 53.09%
North America 52.74%
North Africa 46.46%
Mexico 28.51%
Korea; South 54.09%
Japan 51.73%
Italy 69.27%
Israel 37.77%
Iran 29.75%
India 37.54%
Europe 62.51%
England 69.63%
East Asia 51.80%
East Africa 42.14%
China 51.59%
Central Africa 35.58%
Brazil 49.69%
Australia 51.98%
0.00%
10.00%
20.00%
30.00%
40.00%
50.00%
60.00%
70.00%
80.00%

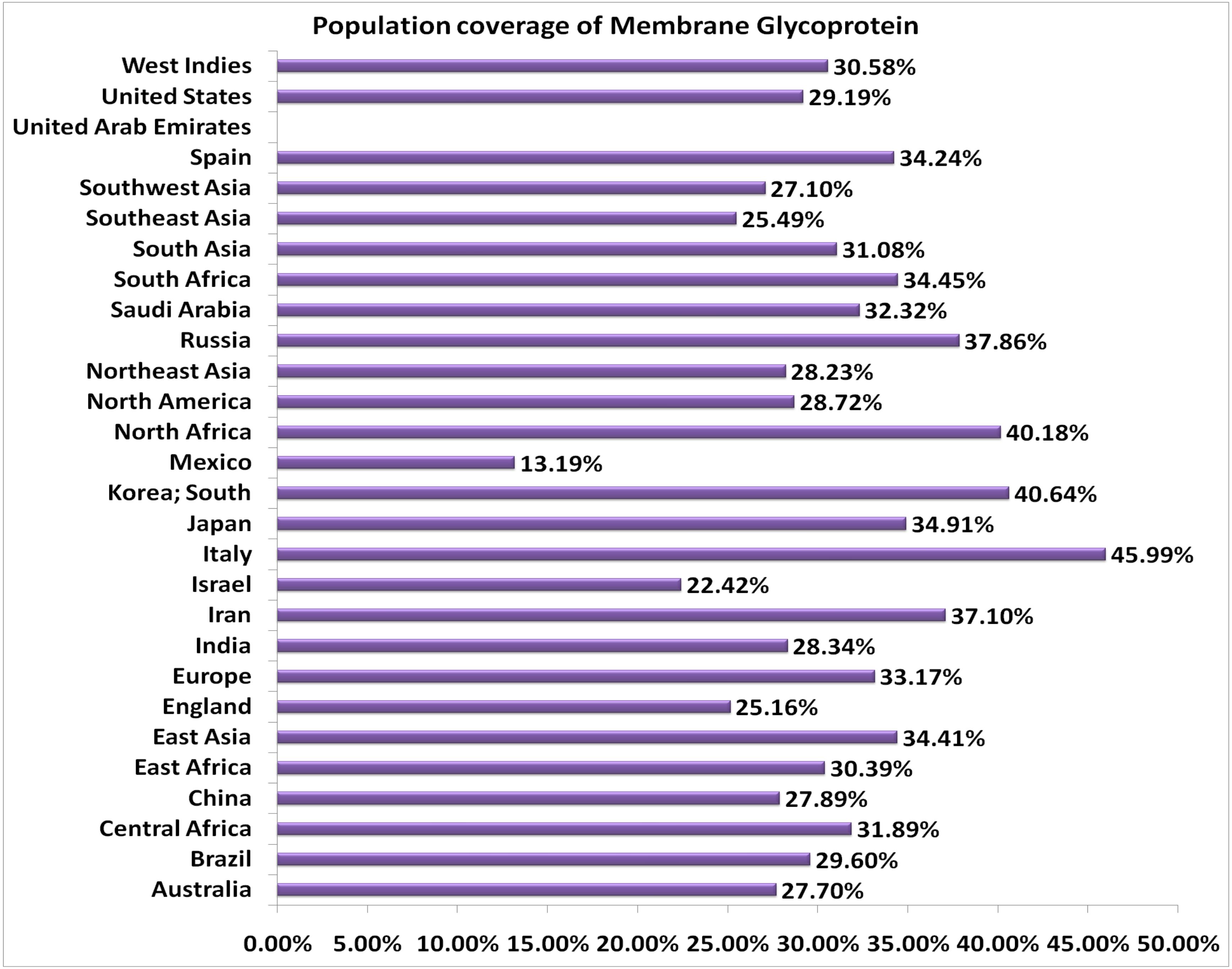

Population coverage of Membrane Glycoprotein
West Indies
United States
United Arab Emirates
Spain
Southwest Asia
Southeast Asia
South Asia
South Africa
Saudi Arabia
Russia
Northeast Asia
North America
North Africa
Mexico
Korea; South
Japan
Italy
Israel
Iran
India
Europe
England
East Asia
East Africa
China
Central Africa
Brazil
Australia
30.58%
29.19%
34.24%
27.10%
25.49%
31.08%
34.45%
32.32%
37.86%
28.23%
28.72%
40.18%
13.19%
40.64%
34.91%
45.99%
22.42%
37.10%
28.34%
33.17%
25.16%
34.41%
30.39%
27.89%
31.89%
29.60%
27.70%
0.00%
5.00%
10.00%
15.00%
20.00%
25.00%
30.00%
35.00%
40.00%
45.00%
50.00%

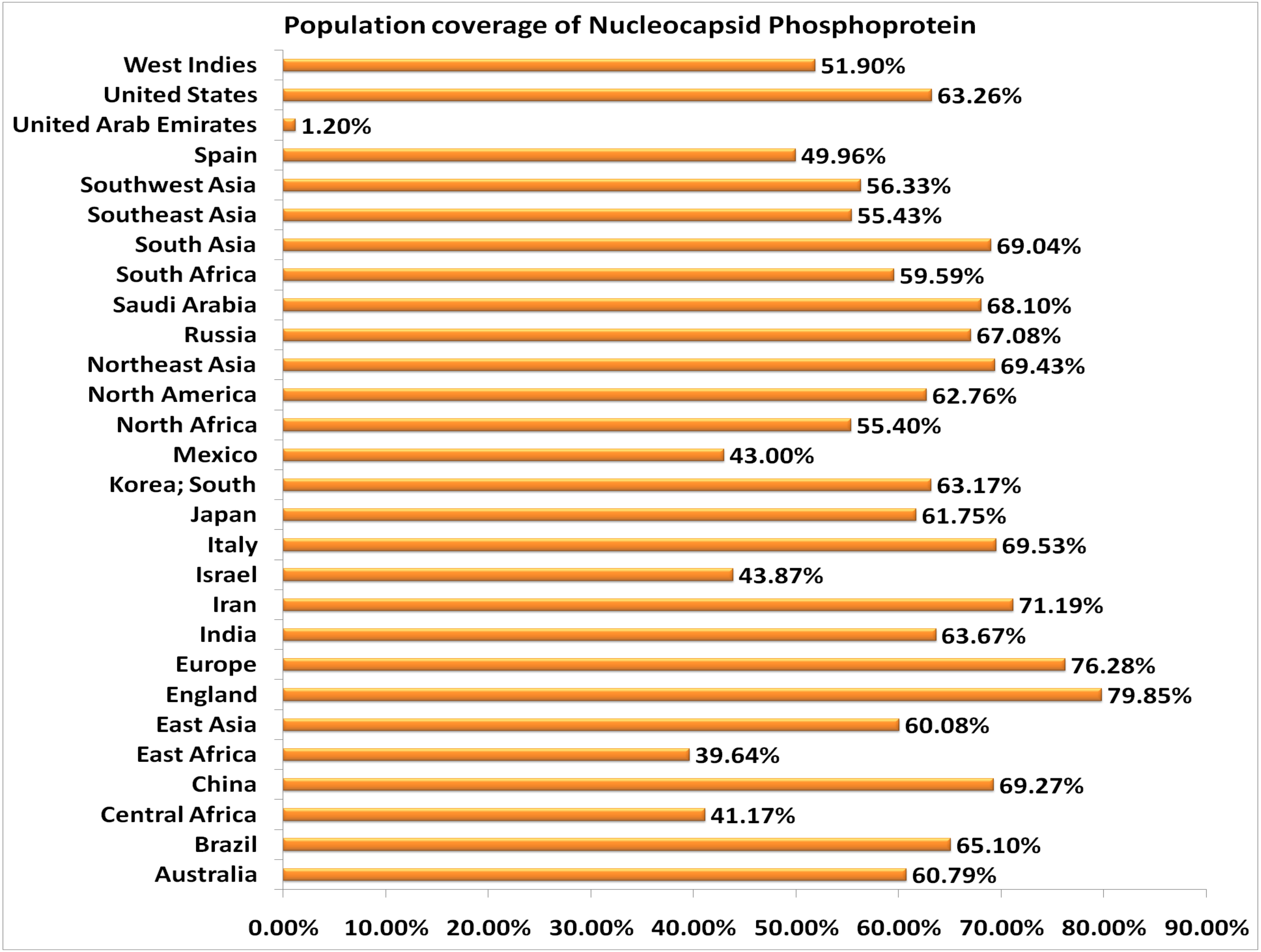
Population coverage of Nucleocapsid Phosphoprotein
West Indies
51.90%
United States
63.26%
United Arab Emirates
1.20%
Spain
49.96%
Southwest Asia
56.33%
Southeast Asia
55.43%
South Asia
69.04%
South Africa
59.59%
Saudi Arabia
68.10%
Russia
67.08%
Northeast Asia
69.43%
North America
62.76%
North Africa
55.40%
Mexico
43.00%
Korea; South
63.17%
Japan
61.75%
Italy
69.53%
Israel
43.87%
Iran
71.19%
India
63.67%
Europe
76.28%
England
79.85%
East Asia
60.08%
East Africa
39.64%
China
69.27%
Central Africa
41.17%
Brazil
65.10%
Australia
60.79%
0.00%
10.00%
20.00%
30.00%
40.00%
50.00%
60.00%
70.00%
80.00%
90.00%
