# Supplementary figures and images for: Computational perspectives revealed prospective vaccine candidates from five structural proteins of novel SARS corona virus 2019 (SARS-CoV-2)
Source: PeerJ. 2020 Sep 29;8:e9855. doi: 10.7717/peerj.9855 (PMC7531350; doi:10.7717/peerj.9855)

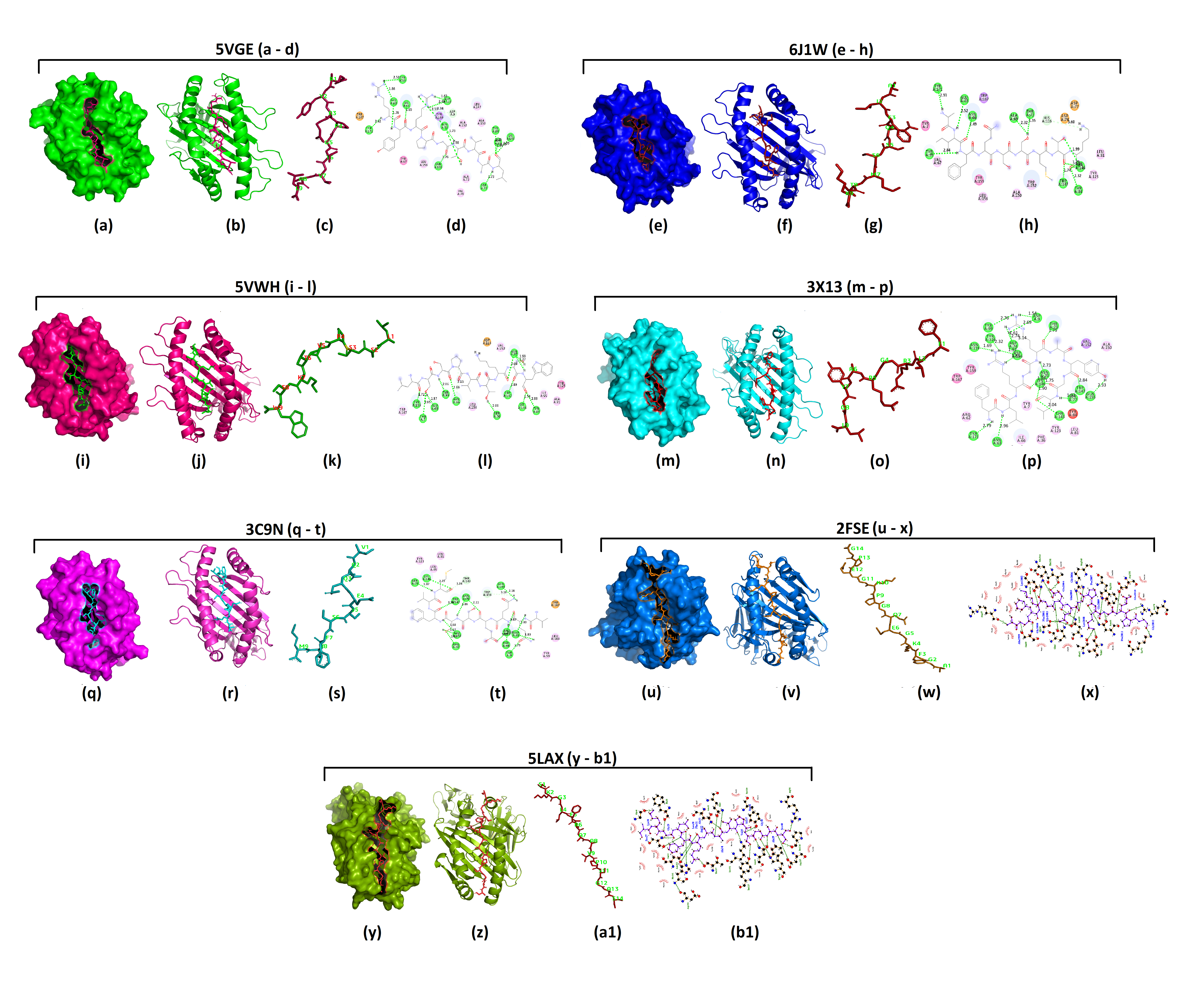

Supplement: Supplemental Information 2 — HADDOCK 2.4 was used for the re-docking using the MHC I and II alleles and the corresponding ligands as available in the form of crystal structures submitted to PDB. 3D structures of best clusters were then visualized using PYMOL 2.3.4 and 2D interaction map was visualized using Discovery Studio tools (MHC I epitopes)** and Ligplot+ v.1.4.5 (for MHC II epitopes)##. Images pertaining to peptide and HLA allele from 5VGE (a-d); (A) 3D structure of surface of the chain ‘A’ from MHC-I HLA allele, “HLA-C*07:02” and the sticky form of peptide “RYRPGTVAL” derived from crystal structure-5VGE (PDB-ID) (B) peptide “RYRPGTVAL“ and the chain ‘A’ of HLA-C*07:02 in 3D cartoon structure (C) Sticky form of peptide “RYRPGTVAL” with positions of residues (D) 2D interaction map of peptide “RYRPGTVAL” and the residues from chain ‘A’ of HLA-C*07:02. Images pertaining to peptide and HLA allele from 6J1W (e-h); (E) 3D structure of surface of the chain ‘A’ of “HLA-A*30:01” and the sticky form of peptide “AIFQSSMTK” from 6J1W (F) peptide “AIFQSSMTK” and the chain ‘A’ of “HLA-A*30:01” in 3D cartoon structure (G­) Sticky form of peptide “AIFQSSMTK” with positions of residues (H) 2D interaction map of peptide “AIFQSSMTK” and the residues from chain ‘A’ of HLA-A*30:01. Images of peptide and HLA allele from 5VWH (i-l); (I) 3D structure of surface of the chain ‘A’ of “HLA-B*58:01” and the sticky form of peptide “LSSPVTKSW” from 5VWH (J) peptide “LSSPVTKSW” and the chain ‘A’ from “HLA-B*58:01” in 3D cartoon structure (K) sticky form of peptide “LSSPVTKSW” with positions of residues (L) 2D interaction map of peptide “LSSPVTKSW” and the residues from chain ‘A’ of HLA-B*58:01. Images of peptide and HLA allele from 3X13 (m-p); (M) 3D structure of surface of the chain ‘A’ from “HLA-B*08:01” and the sticky form of peptide “FLRGRAYGL” from 3X13 (N) peptide “FLRGRAYGL” and the chain ‘A’ of “HLA-B*08:01” in the 3D cartoon structure (O) Sticky form of peptide “FLRGRAYGL” with positions of residues (P) 2D int [file peerj-08-9855-s002.png]
