# Supplementary material for: Computational perspectives revealed prospective vaccine candidates from five structural proteins of novel SARS corona virus 2019 (SARS-CoV-2)
Source: PeerJ. 2020 Sep 29;8:e9855. doi: 10.7717/peerj.9855 (PMC7531350; doi:10.7717/peerj.9855)
Supplement: Supplemental Information 3 [file peerj-08-9855-s003.docx]

| **SARS-CoV-2 Proteins** | **Position** | **Vaxijen score** |
| --- | --- | --- |
| **Surface glycoprotein (S)** | **21563-25384** | **0.4646** |
| **Orf3A Protein** | **25393-26220** | **0.4945** |
| **Envelope protein (E)** | **26245-26472** | **0.6025** |
| **Membrane glycoprotein (M)** | **26523-27191** | **0.5102** |
| **ORF6 protein** | **27202-27387** | **0.6131** |
| **Nucleocapsid phosphoprotein (N)** | **28274-29533** | **0.5059** |

**Table-S1: Antigenicity score of different structural proteins from SARS-CoV-2 predicted by VaxiJen tool**
