# Supplementary material for: Computational perspectives revealed prospective vaccine candidates from five structural proteins of novel SARS corona virus 2019 (SARS-CoV-2)
Source: PeerJ. 2020 Sep 29;8:e9855. doi: 10.7717/peerj.9855 (PMC7531350; doi:10.7717/peerj.9855)
Supplement: Supplemental Information 4 — The combined score was provided by the Net-CTL server. [file peerj-08-9855-s004.docx]

Supplementary Table- S2: All predicted CD8 + T cell epitopes and their antigenicity and immunogenicity scores. The combined score was provided by the Net-CTL server.

| **Surface glycoprotein** | | | | |
| --- | --- | --- | --- | --- |
| **EPITOPE** | **POSITION** | **Antigenicity (Vaxijen Score)** | **immunogenicity** | **Combined score**  **(Net-CTL Score)** |
| AEIRASANL | 1016 | 0.7082 | 0.00689 | 1.8005 |
| AEVQIDRLI | 989 | -0.5562 | 0.08452 | 1.2946 |
| AYSNNSIAI | 706 | 0.8274 | -0.08706 | 1.5591 |
| CNDPFLGVY | 136 | 0.4295 | 0.15232 | 1.3355 |
| CVADYSVLY | 361 | -0.0293 | -0.09595 | 2.5759 |
| DEDDSEPVL | 1257 | 0.5104 | -0.02257 | 1.3047 |
| ECSNLLLQY | 748 | 0.3331 | -0.15849 | 1.5378 |
| EPVLKGVKL | 1262 | 1.2301 | -0.26702 | 1.2802 |
| ETKCTLKSF | 298 | 0.8720 | -0.37555 | 1.5231 |
| EYVSQPFLM | 169 | 0.2605 | -0.18399 | 1.7025 |
| FAMQMAYRF | 898 | 1.0278 | -0.28061 | 1.5848 |
| FERDISTEI | 464 | -0.7442 | 0.10425 | 1.4106 |
| FEYVSQPFL | 168 | 0.6324 | -0.17076 | 1.8668 |
| FLHVTYVPA | 1062 | 1.3346 | 0.11472 | 1.3772 |
| FQPTNGVGY | 497 | 0.3114 | 0.1157 | 1.2969 |
| FRKSNLKPF | 456 | 0.6280 | -0.44169 | 1.395 |
| FTNVYADSF | 392 | -0.5651 | -0.00527 | 1.4262 |
| FVFKNIDGY | 192 | -0.1304 | -0.0215 | 2.2795 |
| FVSNGTHWF | 1095 | 0.0807 | 0.16605 | 1.2938 |
| GAEHVNNSY | 652 | 0.9347 | -0.00296 | 1.996 |
| GEVFNATRF | 339 | -0.1511 | 0.22473 | 1.7314 |
| GQTGKIADY | 413 | 1.4019 | 0.00796 | 1.3104 |
| GRLQSLQTY | 999 | -0.0743 | -0.36678 | 1.7162 |
| GTITSGWTF | 880 | 0.3272 | 0.16268 | 1.5724 |
| GVYFASTEK | 89 | 0.7112 | 0.09023 | 1.4615 |
| GVYYHKNNK | 142 | 0.8264 | -0.18566 | 1.3335 |
| GVYYPDKVF | 35 | 0.0652 | -0.15272 | 1.2993 |
| HADQLTPTW | 625 | 0.3807 | -0.0703 | 1.7854 |
| IAIPTNFTI | 712 | 0.7052 | 0.18523 | 1.5865 |
| IPTNFTISV | 714 | 0.8820 | 0.17229 | 1.5427 |
| IYQTSNFRV | 312 | 0.3109 | -0.03669 | 1.6294 |
| KCYGVSPTK | 378 | 1.4199 | -0.06931 | 1.2722 |
| KIADYNYKL | 417 | 1.6639 | -0.10379 | 1.4347 |
| KTSVDCTMY | 733 | 1.1824 | -0.11115 | 2.3795 |
| KVFRSSVLH | 41 | -0.6913 | -0.19839 | 1.3419 |
| LAGTITSGW | 878 | 0.3218 | 0.09638 | 1.4057 |
| LGAENSVAY | 699 | 0.4173 | 0.00912 | 1.2832 |
| LLALHRSYL | 241 | 0.5241 | -0.06002 | 1.4391 |
| LPPAYTNSF | 24 | 0.3775 | -0.03341 | 1.5189 |
| LTDEMIAQY | 865 | 0.1043 | 0.02757 | 3.6616 |
| MIAQYTSAL | 869 | 0.1114 | -0.18768 | 1.3248 |
| MTSCCSCLK | 1237 | 0.4270 | -0.36816 | 1.336 |
| NGVEGFNCY | 481 | 0.7783 | 0.22039 | 1.4016 |
| NIDGYFKIY | 196 | -0.2462 | 0.04366 | 1.9606 |
| NSASFSTFK | 370 | 0.1232 | -0.09434 | 1.3454 |
| NSFTRGVYY | 30 | -0.1877 | 0.19204 | 1.6915 |
| NSIAIPTNF | 710 | 0.1744 | 0.23071 | 1.6445 |
| NYLYRLFRK | 450 | -0.8611 | 0.16168 | 1.3274 |
| NYNYLYRLF | 448 | -0.1480 | 0.0171 | 1.9482 |
| PFFSNVTWF | 57 | 0.6638 | 0.06627 | 1.4947 |
| PYRVVVLSF | 507 | 1.0281 | 0.03138 | 1.8786 |
| QIYKTPPIK | 787 | -0.0833 | -0.12244 | 1.4526 |
| QLTPTWRVY | 628 | 1.2119 | 0.31555 | 1.3281 |
| QSAPHGVVF | 1054 | 0.2234 | 0.1239 | 1.64 |
| QYIKWPWYI | 1208 | 1.4177 | 0.21624 | 1.8109 |
| RLFRKSNLK | 454 | -0.2829 | -0.28759 | 1.7563 |
| RLQSLQTYV | 1000 | -0.2167 | -0.29331 | 1.2727 |
| RRARSVASQ | 682 | 0.5490 | -0.1211 | 1.572 |
| RSFIEDLLF | 815 | -0.5782 | 0.27446 | 1.9914 |
| SANNCTFEY | 162 | -0.0924 | 0.13273 | 1.8739 |
| SIIAYTMSL | 691 | 0.5234 | -0.12935 | 1.3658 |
| SPRRARSVA | 680 | 0.7729 | 0.0402 | 1.5619 |
| STECSNLLL | 746 | 0.4871 | -0.20478 | 2.3492 |
| SVLYNSASF | 366 | 0.1857 | -0.23299 | 1.2612 |
| TFEYVSQPF | 167 | 0.6641 | -0.19099 | 1.6168 |
| TLKSFTVEK | 302 | 0.0809 | 0.00741 | 1.3483 |
| TLLALHRSY | 240 | 0.8009 | 0.00244 | 1.3654 |
| TRFQTLLAL | 236 | 0.3406 | -0.0377 | 1.6214 |
| TSNQVAVLY | 604 | 0.4387 | -0.01327 | 3.0758 |
| VASQSIIAY | 687 | 0.1366 | -0.0709 | 1.7978 |
| VFVSNGTHW | 1094 | 0.3438 | -0.07581 | 1.2876 |
| VLNDILSRL | 976 | -0.8524 | 0.03 | 1.3533 |
| VTYVPAQEK | 1065 | 0.8132 | 0.02711 | 1.396 |
| VYAWNRKRI | 350 | 0.5003 | 0.12625 | 1.5755 |
| VYSTGSNVF | 635 | -0.3099 | -0.11871 | 1.8571 |
| WMESEFRVY | 152 | 0.2698 | 0.14153 | 1.9232 |
| WPWYIWLGF | 1212 | 1.4953 | 0.41673 | 1.2753 |
| WTAGAAAYY | 258 | 0.6306 | 0.15259 | 3.1128 |
| YFPLQSYGF | 489 | 0.5107 | -0.26661 | 1.6931 |
| YLQPRTFLL | 269 | 0.4532 | 0.1305 | 1.5152 |
| YQPYRVVVL | 505 | 0.5964 | 0.1409 | 1.9051 |
| YRLFRKSNL | 453 | 0.0522 | -0.1818 | 1.6065 |
| YSSANNCTF | 160 | -0.1036 | -0.04954 | 1.9531 |
| **ORF3a protein** | | | | |
| **EPITOPE** | **POSITION** | **Antigenicity score** | **Immunogenicity** | **Combined score** |
| FTIGTVTLK | 8 | 2.0317 | 0.18024 | 1.298 |
| IPIQASLPF | 35 | 0.9697 | -0.20683 | 1.7242 |
| IQASLPFGW | 37 | 1.4578 | -0.05641 | 1.6591 |
| KIITLKKRW | 61 | 0.9074 | -0.2833 | 1.333 |
| LKKRWQLAL | 65 | 1.0692 | 0.10224 | 1.5823 |
| KRWQLALSK | 67 | -0.0172 | -0.12465 | 1.983 |
| ALSKGVHFV | 72 | 1.0008 | -0.10314 | 1.3158 |
| VHFVCNLLL | 77 | 0.7487 | 0.00511 | 1.6281 |
| FVCNLLLLF | 79 | 0.1047 | -0.06109 | 1.4309 |
| LLLLFVTVY | 83 | 0.3277 | 0.19498 | 1.2826 |
| LFVTVYSHL | 86 | -0.0495 | -0.03154 | 1.3048 |
| APFLYLYAL | 103 | 0.4046 | 0.03254 | 1.471 |
| FLYLYALVY | 105 | 0.1273 | 0.03563 | 1.5379 |
| LYLYALVYF | 106 | 0.1541 | 0.05302 | 1.8319 |
| YLYALVYFL | 107 | 0.0134 | 0.13151 | 1.4887 |
| LYALVYFLQ | 108 | 0.0247 | 0.13058 | 1.3717 |
| VYFLQSINF | 112 | 1.1117 | -0.13315 | 1.7629 |
| VRIIMRLWL | 121 | -0.5413 | 0.1749 | 1.332 |
| IIMRLWLCW | 123 | -0.2766 | 0.15193 | 1.6735 |
| IMRLWLCWK | 124 | -0.0759 | 0.29482 | 1.4803 |
| LLYDANYFL | 139 | -0.03 | 0.11841 | 1.5602 |
| FLCWHTNCY | 146 | 0.8927 | 0.23647 | 1.4805 |
| PYNSVTSSI | 159 | 0.2647 | -0.32811 | 1.5605 |
| TSPISEHDY | 176 | 0.4588 | 0.10373 | 1.6929 |
| YQIGGYTEK | 184 | 1.0504 | 0.19808 | 1.3104 |
| HSYFTSDYY | 204 | 0.1808 | 0.01523 | 1.7776 |
| FTSDYYQLY | 207 | -0.1135 | -0.1427 | 3.747 |
| TSDYYQLYS | 208 | 0.1845 | -0.12068 | 1.689 |
| YYQLYSTQL | 211 | 0.4644 | -0.24301 | 1.6394 |
| STDTGVEHV | 220 | 0.6575 | 0.22152 | 1.9456 |
| HVTFFIYNK | 227 | 0.9862 | 0.36278 | 1.3031 |
| EEHVQIHTI | 241 | 0.0295 | 0.1145 | 1.3214 |
| HTIDGSSGV | 247 | 0.5786 | -0.17703 | 1.6951 |
| **Envelope protein** | | | | |
| **EPITOPE** | **POSITION** | **Antigenicity score** | **Immunogenicity** | **Combined score** |
| SEETGTLIV | 6 | 0.3052 | 0.2095 | 1.4227 |
| LLFLAFVVF | 18 | 0.8144 | 0.2341 | 1.2517 |
| FLAFVVFLL | 20 | 0.5308 | 0.30188 | 1.4408 |
| VFLLVTLAI | 25 | 0.8134 | 0.07548 | 1.5566 |
| FLLVTLAIL | 26 | 0.9645 | 0.17608 | 1.4215 |
| LTALRLCAY | 34 | 0.2825 | 0.01886 | 2.6158 |
| VSLVKPSFY | 49 | 0.7476 | -0.25372 | 1.7149 |
| SLVKPSFYV | 50 | 0.414 | -0.27349 | 1.2831 |
| LVKPSFYVY | 51 | 0.4213 | -0.11106 | 1.3262 |
| YVYSRVKNL | 57 | 0.702 | -0.26419 | 1.3968 |
| **Membrane glycoprotein** | | | | |
| **EPITOPE** | **POSITION** | **Antigenicity score** | **Immunogenicity** | **Combined score** |
| LTWICLLQF | 29 | 1.1393 | 0.06584 | 1.3476 |
| WICLLQFAY | 31 | 0.7317 | -0.02684 | 1.4105 |
| FAYANRNRF | 37 | 0.7785 | 0.10537 | 1.2776 |
| AYANRNRFL | 38 | 0.2181 | 0.16258 | 1.2548 |
| YANRNRFLY | 39 | 0.0256 | 0.18472 | 1.6155 |
| NRFLYIIKL | 43 | 0.3128 | 0.13484 | 1.586 |
| RFLYIIKLI | 44 | 0.4257 | 0.05908 | 1.5176 |
| FLYIIKLIF | 45 | 0.3056 | 0.12772 | 1.4667 |
| LYIIKLIFL | 46 | 0.4865 | 0.1374 | 1.4294 |
| YIIKLIFLW | 47 | 0.1245 | 0.033 | 1.7624 |
| KLIFLWLLW | 50 | 0.4968 | 0.34287 | 1.6986 |
| LWLLWPVTL | 54 | 0.7197 | 0.24802 | 1.3976 |
| LWPVTLACF | 57 | 1.159 | 0.06682 | 1.645 |
| LAAVYRINW | 67 | 1.4322 | 0.2079 | 1.9258 |
| MACLVGLMW | 84 | 0.7889 | -0.06852 | 1.498 |
| LVGLMWLSY | 87 | 1.0633 | -0.06867 | 1.3974 |
| GLMWLSYFI | 89 | 0.2537 | 0.06464 | 1.3055 |
| WLSYFIASF | 92 | -0.1644 | 0.11822 | 1.4065 |
| LSYFIASFR | 93 | 0.3283 | 0.21181 | 1.4994 |
| SYFIASFRL | 94 | 0.4821 | 0.18333 | 1.7172 |
| YFIASFRLF | 95 | -0.1142 | 0.06887 | 1.7536 |
| RLFARTRSM | 101 | 0.1998 | 0.11133 | 1.3243 |
| ARTRSMWSF | 104 | 1.2394 | -0.17144 | 1.3676 |
| RIAGHHLGR | 150 | -0.4947 | 0.11919 | 1.2901 |
| VATSRTLSY | 170 | 0.9457 | -0.17295 | 1.4642 |
| ATSRTLSYY | 171 | 0.6108 | -0.11604 | 2.6146 |
| SRTLSYYKL | 173 | 0.3474 | -0.29226 | 1.4734 |
| QRVAGDSGF | 185 | 0.0861 | -0.01317 | 1.3418 |
| YSRYRIGNY | 196 | 0.3996 | 0.21358 | 1.6623 |
| SRYRIGNYK | 197 | -0.141 | 0.20476 | 1.4006 |
| SSDNIALLV | 213 | 0.338 | 0.15128 | 2.9325 |
| **ORF6 protein** | | | | |
| **EPITOPE** | **POSITION** | **Antigenicity score** | **Immunogenicity** | **Combined score** |
| HLVDFQVTI | 3 | 1.4119 | 0.0982 | 1.2661 |
| LLIIMRTFK | 15 | 0.4377 | 0.156 | 1.3525 |
| KVSIWNLDY | 23 | 0.8195 | 0.29343 | 2.6352 |
| IIKNLSKSL | 36 | -0.3592 | -0.5217 | 1.3011 |
| **Nucelocapsid phosphoprotein** | | | | |
| **EPITOPE** | **POSITION** | **Antigenicity score** | **Immunogenicity** | **Combined score** |
| QRNAPRITF | 9 | 0.4654 | 0.21019 | 1.6151 |
| LPNNTASWF | 45 | -0.0835 | 0.05582 | 1.2663 |
| NTASWFTAL | 48 | -0.0708 | 0.22775 | 1.2834 |
| FPRGQGVPI | 66 | 0.7585 | -0.00164 | 1.647 |
| SSPDDQIGY | 78 | 0.526 | 0.0634 | 1.8805 |
| YYRRATRRI | 86 | -0.6565 | 0.21744 | 1.4885 |
| YRRATRRIR | 87 | -0.2931 | 0.26413 | 1.3008 |
| RRIRGGDGK | 92 | 0.2078 | 0.1987 | 1.5726 |
| KMKDLSPRW | 100 | 1.7462 | -0.19333 | 1.4454 |
| DLSPRWYFY | 103 | 1.7645 | 0.25933 | 1.4994 |
| LSPRWYFYY | 104 | 1.2832 | 0.35734 | 2.3408 |
| SPRWYFYYL | 105 | 0.734 | 0.34101 | 1.6154 |
| GANKDGIIW | 124 | -0.6134 | 0.02448 | 1.3086 |
| GTTLPKGFY | 164 | 0.0225 | -0.11536 | 1.6848 |
| LLLDRLNQL | 222 | 0.1566 | -0.01446 | 1.2648 |
| QQQGQTVTK | 240 | 0.6709 | -0.02224 | 1.2955 |
| KSAAEASKK | 249 | 0.7679 | -0.07922 | 1.4421 |
| KPRQKRTAT | 257 | 0.2029 | -0.20542 | 1.6339 |
| KAYNVTQAF | 266 | 0.5669 | -0.00587 | 1.8413 |
| ELIRQGTDY | 290 | -0.2497 | 0.0601 | 1.9108 |
| QFAPSASAF | 306 | 0.5495 | -0.23949 | 1.2686 |
| TWLTYTGAI | 329 | 0.5439 | 0.11986 | 1.5146 |
| LLNKHIDAY | 352 | -0.3003 | -0.02074 | 1.3867 |
| KTFPPTEPK | 361 | 0.7571 | 0.1306 | 1.4314 |
| KKADETQAL | 374 | 0.4135 | 0.09416 | 1.7029 |
| FSKQLQQSM | 403 | 0.1526 | -0.50082 | 1.2806 |
