# Supplementary material for: Computational perspectives revealed prospective vaccine candidates from five structural proteins of novel SARS corona virus 2019 (SARS-CoV-2)
Source: PeerJ. 2020 Sep 29;8:e9855. doi: 10.7717/peerj.9855 (PMC7531350; doi:10.7717/peerj.9855)
Supplement: Supplemental Information 5 [file peerj-08-9855-s005.docx]

**Supplementary Table-S3: CD8+ T-cell epitopes derived from different structural proteins of SARS-CoV-2, their combined score, MHC-I alleles, conservancy and allergenicity**

| **Surface glycoprotein** | | | | | | | |
| --- | --- | --- | --- | --- | --- | --- | --- |
| **NO.** | **Epitopes** | **Combined score** | **MHC-I interaction with an affinity of IC50 < 250 and total score** | **Conservancy** | **Position** | **Allergenicity** | |
|  |  |  |  |  |  | **AllerTOP** | **AllergenFP** |
|  | LTDEMIAQY | 3.6616 | HLA-C*12:03, 6.5(3.2)  HLA-C*05:01, 10.51(1.5)  HLA-A*01:01, 67.07(0.2)  HLA-C*14:02, 178.08(39) | 77.78% | 865-873 | NO | YES |
|  | WTAGAAAYY | 3.1128 | HLA-C*03:03, 6.81(2.7)  HLA-A*29:02, 25.27(0.3)  HLA-C*12:03, 52.74(75)  HLA-A*30:02, 58.81(0.2)  HLA-B*15:02, 71.81(5.5)  HLA-A*26:01, 78.45(0.2)  HLA-B*35:01, 83.09(1.2)  HLA-A*68:01, 141.41(2.1)  HLA-C*14:02, 195.26(43)  HLA-B*15:01, 244.9(1.6)  HLA-A*01:01, 248.62(0.3) | 55.56% | 258-266 | NO | NO |
|  | YLQPRTFLL | 1.9947 | HLA-A*02:01, 11.99(0.3)  HLA-B*15:02, 25.89(1.1)  HLA-A*02:06, 50.06(1.7)  HLA-C*03:03, 53.77(36)  HLA-C*07:02, 77.29(2.5)  HLA-C*12:03, 84.56(94)  HLA-C*14:02, 122.07(27) | 66.67% | 269-277 | YES | YES |
|  | WMESEFRVY | 1.9232 | HLA-C*12:03, 30.56(44)  HLA-C*14:02, 63.62(13)  HLA-C*03:03, 68.48(43)  HLA-B*15:01, 118.57(0.8)  HLA-B*35:01, 177.24(2.2)  HLA-A*29:02, 227.9(1.4)  HLA-C*07:02, 247.26(16) | 44.44% | 152-160 | YES | YES |
|  | YQPYRVVVL | 1.9051 | HLA-C*12:03, 37.77(59)  HLA-A*02:06, 68.16(2.3)  HLA-B*39:01, 75.92(0.5)  HLA-B*15:02, 92.94(7.4)  HLA-B*15:01, 181.97(1.2)  HLA-C*14:02, 198.89(43)  HLA-C*03:03, 199.8(70) | 100.00% | 505-513 | NO | NO |
|  | PYRVVVLSF | 1.8786 | HLA-C*14:02, 9.45(1.4)  HLA-A*23:01, 46.71(0.3)  HLA-C*12:03, 61.97(83)  HLA-C*07:02, 66.86(2)  HLA-B*15:02, 173.87(14)  HLA-A*24:02, 193.92(0.4) | 100.00% | 507-515 | NO | NO |
|  | QYIKWPWYI | 1.8109 | HLA-A*23:01, 15.36(0.2)  HLA-C*12:03, 33.82(51)  HLA-C*14:02, 39.41(6.3)  HLA-A*24:02, 40.42(0.2)  HLA-C*03:03, 71.22(44) | 88.89% | 1208-1216 | YES | NO |
|  | AEIRASANL | 1.8005 | HLA-C*03:03, 11.98(6.1)  HLA-B*40:01, 25.87(0.2)  HLA-B*15:02, 55.87(3.9)  HLA-C*12:03, 70.01(88)  HLA-B*40:02, 93.51(0.6)  HLA-B*44:03, 174.37(0.3) | 100.00% | 1016-1024 | NO | YES |
|  | NSIAIPTNF | 1.6445 | HLA-C*03:03, 26.64(19)  HLA-C*12:03, 66.55(86)  HLA-C*15:02, 190.29(12)  HLA-B*58:01, 223.51(1.4) | 88.89% | 710-718 | YES | NO |
|  | QSAPHGVVF | 1.64 | HLA-C*03:03, 35.53(25)  HLA-B*15:01, 60.53(0.4)  HLA-B*15:02, 76.24(5.9)  HLA-B*58:01, 106.73(0.8)  HLA-C*12:03, 122.8(99)  HLA-A*32:01, 185.75(1.4)  HLA-C*15:02, 201.56(13) | 88.89% | 1054-1062 | YES | YES |
|  | IAIPTNFTI | 1.5865 | HLA-C*03:03, 6.02(2.2)  HLA-C*12:03, 11.04(13)  HLA-B*58:01, 26.62(0.3)  HLA-B*53:01, 120.74(0.5)  HLA-C*15:02, 126.3(7.9)  HLA-A*02:06, 186.44(5.4) | 88.89% | 712-720 | NO | YES |
|  | VYAWNRKRI | 1.5755 | HLA-C*14:02, 14.3(2.4)  HLA-C*12:03, 75.19(91)  HLA-C*03:03, 129.6(60)  HLA-A*23:01, 163.46(0.6)  HLA-A*24:02, 214.1(0.4) | 77.78% | 350-358 | YES | YES |
|  | GTITSGWTF | 1.5724 | HLA-C*03:03, 45.45(32)  HLA-C*12:03, 70.5(89)  HLA-B*58:01, 91.47(0.7)  HLA-B*15:02, 145.28(12) | 77.78% | 880-888 | YES | YES |
|  | SPRRARSVA | 1.5619 | HLA-B*07:02, 5.64(0.1)  HLA-C*12:03, 18.41(23)  HLA-B*15:02, 195.08(15) | 33.33% | 680-688 | NO | YES |
|  | IPTNFTISV | 1.5427 | HLA-C*12:03, 18.24(23)  HLA-B*07:02, 116.92(0.7) | 77.78% | 714-722 | NO | YES |
|  | PFFSNVTWF | 1.4947 | HLA-C*12:03, 39.73(62)  HLA-C*14:02, 48.82(8.2)  HLA-C*07:02, 109.69(4.4)  HLA-B*15:02, 118.36(9.2)  HLA-A*23:01, 133.48(0.5)  HLA-C*03:03, 149.83(64) | 77.78% | 57-65 | YES | YES |
|  | GVYFASTEK | 1.4615 | HLA-C*03:03, 11.47(5.9)  HLA-A*11:01, 27.3(0.4)  HLA-C*12:03, 29.05(41)  HLA-A*03:01, 54.05(0.3)  HLA-C*14:02, 91.54(19)  HLA-C*15:02, 101.02(6.4)  HLA-A*68:01, 123.16(1.9)  HLA-A*30:01, 199.51(3) | 77.78% | 89-97 | NO | NO |
|  | LAGTITSGW | 1.4057 | HLA-C*12:03, 7.83(5.7)  HLA-B*58:01, 24.9(0.3)  HLA-C*03:03, 39.59(28)  HLA-B*53:01, 61.21(0.3)  HLA-C*05:01, 110.83(8.3)  HLA-B*57:01, 146.49(0.4) | 55.56% | 878-786 | NO | NO |
|  | NGVEGFNCY | 1.4016 | HLA-C*12:03, 22.55(29)  HLA-C*03:03, 30.8(22)  HLA-B*15:02, 89.58(7.1)  HLA-B*35:01, 95.62(1.4)  HLA-C*14:02, 113.92(25) | 44.44% | 481-489 | YES | YES |
|  | VTYVPAQEK | 1.396 | HLA-C*03:03, 9.41(4.2)  HLA-C*15:02, 19.33(0.8)  HLA-C*12:03, 26.25(36)  HLA-C*14:02, 29.21(4.7)  HLA-A*11:01, 53(0.6)  HLA-A*03:01, 107.1(0.4)  HLA-A*30:01, 223.34(3.2) | 77.78% | 1065-1073 | YES | YES |
|  | FLHVTYVPA | 1.3772 | HLA-C*03:03, 10.41(5)  HLA-C*14:02, 20.77(3.1)  HLA-C*12:03, 41.41(64)  HLA-A*02:01, 103.3(1.7)  HLA-A*02:06, 192.99(5.5)  HLA-B*15:02, 225.02(17) | 88.89% | 1062-1070 | YES | YES |
|  | TLLALHRSY | 1.3654 | HLA-C*03:03, 22.67(16)  HLA-C*14:02, 42.81(7.1)  HLA-C*12:03, 61.97(83)  HLA-A*29:02, 140.85(1)  HLA-B*15:02, 157.48(13)  HLA-B*15:01, 220.8(1.4) | 33.33% | 240-248 | YES | YES |
|  | TLKSFTVEK | 1.3483 | HLA-C*12:03, 8.37(6.9)  HLA-A*11:01, 101.22(0.9)  HLA-A*68:01, 111.55(1.7)  HLA-A*30:01, 133.34(2)  HLA-C*03:03, 153.67(64)  HLA-A*03:01, 196.69(0.8) | 44.44% | 302-310 | YES | YES |
|  | CNDPFLGVY | 1.3355 | HLA-C*12:03, 3.87(1)  HLA-C*05:01, 52.8(5.4)  HLA-B*15:02, 194.63(15)  HLA-C*14:02, 249.24(51) | 55.56% | 136-144 | YES | NO |
|  | QLTPTWRVY | 1.3281 | HLA-C*03:03, 29.48(21)  HLA-C*12:03, 44.17(68)  HLA-C*14:02, 54.77(9.5)  HLA-B*15:02, 90.2(7.1)  HLA-C*07:02, 121.66(5.1) | 77.78% | 628-636 | NO | NO |
|  | GQTGKIADY | 1.3104 | HLA-C*03:03, 15.68(9)  HLA-C*12:03, 36.07(56)  HLA-A*30:02, 157.92(0.8) | 88.89% | 413-421 | NO | YES |
|  | FQPTNGVGY | 1.2969 | HLA-C*12:03, 13.34(15)  HLA-B*15:02, 73.99(5.7)  HLA-B*15:01, 137.4(0.9) | 55.56% | 497-505 | YES | NO |
|  | FVSNGTHWF | 1.2938 | HLA-B*15:02, 42.68(2.7)  HLA-C*03:03, 55.66(37)  HLA-B*35:01, 57.75(0.9)  HLA-C*12:03, 92.72(96)  HLA-A*02:06, 101.75(3.2)  HLA-C*05:01, 204.96(13)  HLA-C*14:02, 245.82(51) | 77.78% | 1095-1103 | NO | NO |
|  | LGAENSVAY | 1.2832 | HLA-B*35:01, 8.58(0.3)  HLA-C*03:03, 20.92(15)  HLA-C*12:03, 45.94(70)  HLA-B*15:01, 122.17(0.8)  HLA-B*15:02, 148.67(12) | 66.67% | 699-707 | NO | YES |
|  | WPWYIWLGF | 1.2753 | HLA-B*35:01, 9.89(0.3)  HLA-B*53:01, 31.61(0.2)  HLA-C*12:03, 127.7(99)  HLA-B*07:02, 234.9(1.1)  HLA-B*15:02, 239.45(18) | 88.89% | 1212-1220 | YES | YES |
| **ORF3a protein** | | | | | | | |
| **NO.** | **Epitopes** | **Combined score** | **MHC-I interaction with an affinity of IC50 < 250 and total score** | **Conservancy** | **Position** | **Allergenicity** | |
|  |  |  |  |  |  | **AllerTOP** | **AllergenFP** |
|  | STDTGVEHV | 1.9456 | HLA-A*02:06, 142.08(4.4)  HLA-C*14:02, 153.68(35)  HLA-C*12:03, 4.79(1.6)  HLA-C*05:01, 7.27(0.9) | 55.56% | 220-228 | YES | NO |
|  | LYLYALVYF | 1.8319 | HLA-C*14:02, 8.46(1.3)  HLA-A*23:01, 16.42(0.2)  HLA-A*24:02, 69.28(0.2)  HLA-C*03:03, 93.24(52)  HLA-C*12:03, 160.77(100) | 88.89% | 106-114 | NO | YES |
|  | TSPISEHDY | 1.6929 | HLA-C*12:03, 14.86(17)  HLA-C*14:02, 98.54(21)  HLA-B*15:02, 115.4(9)  HLA-A*30:02, 231.98(1.1) | 33.33% | 176-184 | YES | YES |
|  | VHFVCNLLL | 1.6281 | HLA-B*38:01, 33.11(0.2)  HLA-C*12:03, 37.08(58)  HLA-C*07:01, 82.06(4.1)  HLA-B*39:01, 188.53(1.1)  HLA-C*14:02, 189.06(41)  HLA-B*15:02, 234(18) | 66.67% | 77-85 | YES | YES |
|  | LKKRWQLAL | 1.5823 | HLA-C*12:03, 15.6(18)  HLA-B*15:02, 48.33(3.2)  HLA-C*03:03, 107.05(55) | 88.89% | 65-73 | NO | NO |
|  | FLYLYALVY | 1.5379 | HLA-A*29:02, 12.01(0.2)  HLA-C*14:02, 13.47(2.2)  HLA-C*03:03, 26.15(19)  HLA-C*12:03, 26.55(36)  HLA-B*15:01, 35.8(0.2)  HLA-B*15:02, 44.89(2.9)  HLA-B*35:01, 101.76(1.4)  HLA-A*03:01, 132.67(0.6) | 88.89% | 105-113 | NO | YES |
|  | YLYALVYFL | 1.4887 | HLA-A*02:01, 1.08(0.1)  HLA-C*03:03, 12.31(6.4)  HLA-A*02:06, 14.01(0.5)  HLA-B*15:02, 25.24(1)  HLA-C*14:02, 45.25(7.5)  HLA-C*12:03, 85.54(94)  HLA-C*07:02, 201.91(12)  HLA-A*32:01, 248.27(1.7) | 88.89% | 107-115 | NO | YES |
|  | HSYFTSDYY | 1.4211 | HLA-C*12:03, 19.91(25)  HLA-C*03:03, 22.99(17)  HLA-C*14:02, 49.73(8.4)  HLA-C*15:02, 64.62(3.9)  HLA-B*35:01, 66.31(1.1)  HLA-B*15:02, 79.29(6.2)  HLA-A*30:02, 109.51(0.5)  HLA-A*29:02, 173.28(1.1)  HLA-B*58:01, 182.93(1.2)  HLA-B*15:01, 190.54(1.3)  HLA-C*07:01, 203.78(9.5)  HLA-A*68:01, 236.31(3.3) | 66.67% | 204-212 | YES | YES |
|  | LYALVYFLQ | 1.3717 | HLA-C*12:03, 64(84)  HLA-A*24:02, 70.08(0.2)  HLA-C*14:02, 89.45(19)  HLA-C*03:03, 163.15(66) | 88.89% | 108-116 | NO | YES |
|  | FLCWHTNCY | 1.3341 | HLA-B*15:02, 34.05(1.7)  HLA-C*12:03, 40.19(63)  HLA-C*14:02, 59.1(12)  HLA-B*35:01, 68.64(1.1)  HLA-A*29:02, 95.44(0.7)  HLA-C*03:03, 102(54)  HLA-B*15:01, 194.53(1.3) | 66.67% | 146-154 | NO | YES |
|  | EEHVQIHTI | 1.3214 | HLA-C*12:03, 47.22(71)  HLA-C*03:03, 47.49(33)  HLA-B*40:01, 146.18(1) | 66.67% | 241-249 | NO | NO |
|  | YQIGGYTEK | 1.3104 | HLA-C*12:03, 28.78(41)  HLA-A*02:06, 204.43(5.8)  HLA-C*03:03, 7.61(3.2) | 77.78% | 184-192 | NO | YES |
|  | HVTFFIYNK | 1.3031 | HLA-A*68:01, 14.67(0.3)  HLA-A*11:01, 26.74(0.4)  HLA-C*12:03, 43.97(68)  HLA-A*30:01, 75.15(1.3)  HLA-A*31:01, 91.93(1.5)  HLA-C*03:03, 100.14(53)  HLA-A*03:01, 160.62(0.7) | 66.67% | 227-235 | NO | NO |
|  | FTIGTVTLK | 1.298 | HLA-C*03:03, 8.12(3.4)  HLA-A*68:01, 11.05(0.3)  HLA-C*12:03, 36.91(57)  HLA-A*11:01, 55.75(0.6)  HLA-C*14:02, 160.92(36)  HLA-C*07:01, 184.99(8.7) | 44.44% | 08-16 | NO | YES |
|  | LLLLFVTVY | 1.2826 | HLA-C*12:03, 47.77(72)  HLA-C*03:03, 49.04(33)  HLA-B*15:01, 70.95(0.5)  HLA-C*14:02, 149.83(34)  HLA-A*29:02, 170.12(1.1) | 88.89% | 83-91 | NO | YES |
| **Envelope protein** | | | | | | | |
| **NO.** | **Epitopes** | **Combined score** | **MHC-I interaction with an affinity of IC50 < 250 and total score** | **Conservancy** | **Position** | **Allergenicity** | |
|  |  |  |  |  |  | **AllerTOP** | **AllergenFP** |
|  | FLAFVVFLL | 1.4408 | HLA-A*02:01, 2.05(0.2)  HLA-A*02:06, 11.26(0.5)  HLA-B*15:02, 16.83(0.5)  HLA-C*03:03, 23.15(17)  HLA-A*68:02, 34.08(0.5)  HLA-C*12:03, 202.86(100) | 100% | 20-28 | NO | YES |
|  | FLLVTLAIL | 1.4215 | HLA-C*03:03, 15.36(8.8)  HLA-A*02:01, 17.95(0.4)  HLA-B*15:02, 20.33(0.7)  HLA-C*14:02, 50.65(8.6)  HLA-A*02:06, 52.3(1.8)  HLA-C*12:03, 204.74(100) | 100% | 26-34 | NO | YES |
|  | LLFLAFVVF | 1.2517 | HLA-B*15:01, 8.51(0.1)  HLA-A*32:01, 31.91(0.3)  HLA-C*12:03, 48.44(72)  HLA-C*14:02, 121.79(27)  HLA-B*15:02, 157.11(13)  HLA-C*03:03, 166.57(66)  HLA-A*02:06, 220.06(6.2) | 100% | 18-26 | NO | YES |
|  | LTALRLCAY | 2.6158 | HLA-C*03:03, 24.58(18)  HLA-C*12:03, 50.72(74)  HLA-C*14:02, 84.64(18)  HLA-A*01:01, 108.78(0.2)  HLA-B*15:01, 141.9(0.9)  HLA-A*29:02, 148.16(1)  HLA-B*15:02, 150.04(13)  HLA-B*14:02, 173.4(0.4)  HLA-A*30:02, 241.8(1.1) | 100% | 34-42 | NO | NO |
|  | SEETGTLIV | 1.4227 | HLA-C*12:03, 19.46(25)  HLA-C*05:01, 53.78(5.5)  HLA-B*40:01, 92.44(0.7) | 100% | 06--14 | NO | NO |
|  | VFLLVTLAI | 1.5566 | HLA-C*14:02, 18.47(2.9)  HLA-C*12:03, 90.2(95)  HLA-A*23:01, 113.61(0.5)  HLA-C*03:03, 238.56(73) | 100% | 25-33 | NO | YES |
| **Membrane glycoprotein** | | | | | | | |
| **NO.** | **Epitopes** | **Combined score** | **MHC-I interaction with an affinity of IC50 < 250 and total score** | **Conservancy** | **Position** | **Allergenicity** | |
|  |  |  |  |  |  | **AllerTOP** | **AllergenFP** |
|  | SSDNIALLV | 2.9325 | HLA-C*12:03, 4.57(1.5)  HLA-C*05:01, 5.29(0.6)  HLA-C*15:02, 8.88(0.2)  HLA-C*06:02, 53.92(1)  HLA-C*07:01, 59.17(3.1)  HLA-C*08:02, 166.78(1.6)  HLA-A*01:01, 190.78(0.3) | 88.89% | 213-221 | YES | YES |
|  | LAAVYRINW | 1.9258 | HLA-B*58:01, 17.31(0.2)  HLA-C*12:03, 18.88(24)  HLA-C*03:03, 20.92(15)  HLA-B*53:01, 101.12(0.4)  HLA-B*57:01, 119.07(0.4) | 100.00% | 67-75 | YES | YES |
|  | YIIKLIFLW | 1.7624 | HLA-B*58:01, 20.52(0.3)  HLA-C*12:03, 24.11(32)  HLA-C*03:03, 72.54(45)  HLA-B*53:01, 86.67(0.3)  HLA-A*02:01, 162.22(2.3)  HLA-A*02:06, 169.64(5)  HLA-B*57:01, 172.51(0.5) | 88.89% | 47-55 | NO | YES |
|  | SYFIASFRL | 1.7172 | HLA-B*15:02, 23.67(0.9)  HLA-C*14:02, 36.19(5.8)  HLA-C*12:03, 40.19(63)  HLA-C*07:02, 41.89(0.8)  HLA-A*23:01, 186.39(0.6)  HLA-A*24:02, 212.63(0.4) | 88.89% | 94-102 | NO | YES |
|  | KLIFLWLLW | 1.6986 | HLA-B*58:01, 11.46(0.2)  HLA-A*32:01, 22.53(0.3)  HLA-C*12:03, 43.17(67)  HLA-B*57:01, 56.47(0.3) | 88.89% | 50-58 | NO | YES |
|  | YSRYRIGNY | 1.6623 | HLA-C*12:03, 22.09(29)  HLA-B*15:02, 77.84(6)  HLA-C*07:01, 80.56(4.1)  HLA-B*15:01, 115.61(0.8)  HLA-A*30:02, 134.41(0.6)  HLA-C*14:02, 146.08(33) | 88.89% | 196-204 | YES | YES |
|  | LWPVTLACF | 1.645 | HLA-C*14:02, 43.21(7.1)  HLA-C*12:03, 44.79(69)  HLA-A*24:02, 147.1(0.3)  HLA-A*23:01, 227.73(0.7)  HLA-B*15:02, 236.17(18) | 100.00% | 57-65 | NO | YES |
|  | YANRNRFLY | 1.6155 | HLA-C*12:03, 10.89(12)  HLA-B*35:01, 19.75(0.4)  HLA-A*29:02, 29.36(0.3)  HLA-C*03:03, 35.04(25)  HLA-C*05:01, 38.78(4.4)  HLA-C*07:01, 57.69(3)  HLA-B*15:02, 78.92(6.1)  HLA-B*58:01, 162.29(1.1)  HLA-A*30:02, 202.51(0.9)  HLA-C*06:02, 233.24(3.1) | 88.89% | 39-47 | YES | YES |
|  | NRFLYIIKL | 1.586 | HLA-C*07:01, 2.88(0.2)  HLA-C*06:02, 6.93(0.2)  HLA-C*07:02, 23.94(0.2)  HLA-C*12:03, 35.66(55)  HLA-C*14:02, 65.85(13)  HLA-B*15:02, 74.34(5.7)  HLA-B*27:05, 167.32(0.6)  HLA-C*03:03, 234.75(73)  HLA-B*39:01, 245.69(1.3) | 100.00% | 43-51 | NO | YES |
|  | RFLYIIKLI | 1.5176 | HLA-C*03:03, 28.41(20)  HLA-C*14:02, 66.31(13)  HLA-C*12:03, 81.13(93)  HLA-A*23:01, 240.12(0.8) | 88.89% | 44-52 | NO | NO |
|  | LSYFIASFR | 1.4994 | HLA-A*68:01, 3.92(0.2)  HLA-C*03:03, 9.09(4.1)  HLA-A*31:01, 12.2(0.3)  HLA-C*12:03, 22.97(30)  HLA-C*15:02, 46.6(2.8)  HLA-C*14:02, 125.49(28)  HLA-A*03:01, 171.71(0.7)  HLA-A*11:01, 182.09(1.4) | 88.89% | 93-101 | NO | YES |
|  | FLYIIKLIF | 1.4667 | HLA-C*14:02, 25.56(4)  HLA-C*03:03, 46.51(32)  HLA-B*15:02, 77.3(6)  HLA-C*12:03, 95.54(96) | 88.89% | 45-53 | NO | NO |
|  | LYIIKLIFL | 1.4294 | HLA-C*14:02, 17.16(2.8)  HLA-C*03:03, 62.17(40)  HLA-C*12:03, 69.37(88) | 88.89% | 46-64 | NO | YES |
|  | LWLLWPVTL | 1.3976 | HLA-C*03:03, 20.68(15)  HLA-C*12:03, 96.87(96)  HLA-B*15:02, 157.48(13)  HLA-A*23:01, 236.28(0.7)  HLA-C*14:02, 240.22(50) | 100.00% | 54-62 | YES | NO |
|  | LTWICLLQF | 1.3476 | HLA-C*14:02, 129.6(29)  HLA-B*58:01, 141.02(0.9)  HLA-C*12:03, 166.42(100)  HLA-A*32:01, 245.43(1.7) | 77.78% | 29-37 | NO | NO |
|  | RLFARTRSM | 1.3243 | HLA-C*12:03, 22.29(29)  HLA-A*32:01, 42.35(0.4)  HLA-C*03:03, 59.78(39)  HLA-C*07:02, 66.24(2)  HLA-B*15:01, 74.81(0.5)  HLA-B*08:01, 87.81(0.3)  HLA-C*14:02, 100.83(22)  HLA-C*07:01, 156.37(7.7)  HLA-B*14:02, 158.5(0.3) | 100.00% | 101-109 | YES | YES |
|  | GLMWLSYFI | 1.3055 | HLA-A*02:01, 5.15(0.2)  HLA-A*02:06, 35.6(1.1)  HLA-C*12:03, 43.27(67)  HLA-A*32:01, 44.15(0.4)  HLA-C*14:02, 157.26(35) | 88.89% | 89-97 | NO | YES |
|  | FAYANRNRF | 1.2776 | HLA-C*03:03, 2.98(0.8)  HLA-C*12:03, 28.58(40)  HLA-B*15:02, 31.2(1.5)  HLA-B*35:01, 39.68(0.7)  HLA-C*14:02, 57.09(10)  HLA-B*53:01, 249.39(0.9) | 88.89% | 37-45 | YES | YES |
|  | AYANRNRFL | 1.2548 | HLA-C*03:03, 10.95(5.5)  HLA-C*07:02, 47(0.9)  HLA-C*14:02, 55.54(9.6)  HLA-B*15:02, 60.98(4.5)  HLA-C*12:03, 113.29(98)  HLA-C*07:01, 221.9(11) | 88.89% | 38-46 | NO | YES |
| **ORF6 protein** | | | | | | | |
| **NO.** | **Epitopes** | **Combined score** | **MHC-I interaction with an affinity of IC50 < 250 and total score** | **Conservancy** | **Position** | **Allergenicity** | |
|  |  |  |  |  |  | **AllerTOP** | **AllergenFP** |
|  | KVSIWNLDY | 2.6352 | HLA-C*12:03, 25.59(35)  HLA-A*29:02, 44.84(0.4)  HLA-C*05:01, 94.55(7.8)  HLA-A*30:02, 125.15(0.6) | 55.56% | 23-31 | NO | NO |
|  | LLIIMRTFK | 1.3525 | HLA-C*03:03, 47.27(33)  HLA-C*12:03, 48.55(72)  HLA-A*03:01, 103.7(0.4)  HLA-A*11:01, 162.66(1.3)  HLA-A*68:01, 210.61(3) | 77.78% | 15-23 | NO | YES |
|  | HLVDFQVTI | 1.2661 | HLA-C*12:03, 35.01(54)  HLA-C*03:03, 42.71(30)  HLA-A*32:01, 112.18(0.9)  HLA-A*02:01, 116.71(1.8) | 100.00% | 3-11 | YES | NO |
| **Nucleocapsid phosphoprotein** | | | | | | | |
| **NO.** | **Epitopes** | **Combined score** | **MHC-I interaction with an affinity of IC50 < 250 and total score** | **Conservancy** | **Position** | **Allergenicity** | |
|  |  |  |  |  |  | **AllerTOP** | **AllergenFP** |
|  | LSPRWYFYY | 2.3408 | HLA-C*12:03, 14.49(17)  HLA-A*29:02, 116.88(0.8)  HLA-A*01:01, 199.77(0.3)  HLA-B*15:02, 242.78(18) | 100.00% | 104-112 | NO | YES |
|  | SSPDDQIGY | 1.8805 | HLA-C*12:03, 15.21(18)  HLA-C*07:01, 166.78(8)  HLA-C*14:02, 223.16(47) | 88.89% | 78-86 | NO | YES |
|  | KKADETQAL | 1.7029 | HLA-C*03:03, 50.42(34)  HLA-C*12:03, 130.68(99)  HLA-C*07:02, 156.01(7.7)  HLA-B*15:02, 165.28(13)  HLA-B*39:01, 195.16(1.1) | 66.67% | 374-382 | YES | YES |
|  | SPRWYFYYL | 1.6154 | HLA-B*07:02, 20.88(0.2)  HLA-B*08:01, 23.36(0.2)  HLA-B*15:02, 50.96(3.5)  HLA-C*12:03, 92.3(96) | 100.00% | 105-113 | NO | NO |
|  | QRNAPRITF | 1.6151 | HLA-C*07:02, 42.18(0.8)  HLA-C*07:01, 53.35(2.8)  HLA-B*15:02, 61.55(4.5)  HLA-C*06:02, 63.65(1.1)  HLA-C*12:03, 75.02(91)  HLA-C*03:03, 104.13(54)  HLA-C*14:02, 181.39(40) | 88.89% | 9--17 | NO | YES |
|  | RRIRGGDGK | 1.5726 | HLA-C*12:03, 35.17(54)  HLA-B*27:05, 64.65(0.3)  HLA-C*03:03, 75.26(46)  HLA-C*07:02, 235.05(15) | 88.89% | 92-100 | NO | NO |
|  | TWLTYTGAI | 1.5146 | HLA-C*14:02, 16.85(2.7)  HLA-C*03:03, 37.03(26)  HLA-C*12:03, 80.57(93) | 88.89% | 329-337 | NO | NO |
|  | DLSPRWYFY | 1.4994 | HLA-C*03:03, 36.52(26)  HLA-C*12:03, 38.83(61)  HLA-A*29:02, 44.03(0.4)  HLA-C*07:02, 191.94(12)  HLA-B*15:02, 202.87(16)  HLA-A*30:02, 215.5(1) | 88.89% | 103-111 | NO | YES |
|  | KTFPPTEPK | 1.4314 | HLA-A*11:01, 7.31(0.2)  HLA-A*30:01, 11.4(0.2)  HLA-C*12:03, 15.31(18)  HLA-C*14:02, 54.02(9.3)  HLA-A*03:01, 68.35(0.4)  HLA-A*31:01, 90.67(1.5)  HLA-A*68:01, 112.84(1.7)  HLA-C*03:03, 147.43(63)  HLA-A*32:01, 161.78(1.3) | 100.00% | 361-369 | NO | NO |
