# Supplementary material for: Computational perspectives revealed prospective vaccine candidates from five structural proteins of novel SARS corona virus 2019 (SARS-CoV-2)
Source: PeerJ. 2020 Sep 29;8:e9855. doi: 10.7717/peerj.9855 (PMC7531350; doi:10.7717/peerj.9855)
Supplement: Supplemental Information 6 [file peerj-08-9855-s006.docx]

Supplementary Table:S4 Population coverage of the epitopes from SARS-CoV-2and their respective HLA alleles

| **Population/area** | **Surface glycoprotein** | **ORF3a protein** | **Envelope protein** | **Membrane glycoprotein** | **Nucleocapsid phosphoprotein** |
| --- | --- | --- | --- | --- | --- |
| American Samoa | 90.94% | 40.74% | 54.84% | 7.84% | 41.93% |
| American Samoa Polynesian | 90.94% | 40.74% | 54.84% | 7.84% | 41.93% |
| Argentina | 40.46% | 67.89% | 14.05% | 2.09% | 70.08% |
| Argentina Amerindian | 40.46% | 67.89% | 14.05% | 2.09% | 70.08% |
| Australia | 84.12% | 49.59% | 51.98% | 27.70% | 60.79% |
| Australia Australian Aborigines | 84.97% | 46.26% | 41.83% | 23.98% | 50.32% |
| Australia Caucasoid | 79.43% | 54.60% | 68.89% | 29.09% | 79.85% |
| Austria | 60.92% | 39.82% | 41.92% | 10.02% | 67.40% |
| Austria Caucasoid | 60.92% | 39.82% | 41.92% | 10.02% | 67.40% |
| Belgium | 59.30% | 54.03% | 45.86% | 10.32% | 68.61% |
| Belgium Caucasoid | 59.30% | 54.03% | 45.86% | 10.32% | 68.61% |
| Brazil | 72.83% | 52.97% | 49.69% | 29.60% | 65.10% |
| Brazil Amerindian | 79.38% | 74.67% | 16.28% | 16.28% | 74.67% |
| Brazil Caucasoid | 63.60% | 43.30% | 38.30% | 16.92% | 59.52% |
| Brazil Mixed | 72.00% | 46.14% | 55.08% | 32.89% | 64.38% |
| Bulgaria | 82.08% | 38.74% | 36.08% | 32.10% | 54.13% |
| Bulgaria Caucasoid | 55.28% | 30.11% | 21.69% | 28.18% | 49.12% |
| Bulgaria Other | 81.40% | 42.79% | 66.40% | 16.30% | 53.21% |
| Burkina Faso | 15.82% | 15.82% | 9.04% | 20.87% | 22.95% |
| Burkina Faso Black | 15.82% | 15.82% | 9.04% | 20.87% | 22.95% |
| Cameroon | 57.72% | 28.70% | 39.32% | 37.68% | 42.77% |
| Cameroon Black | 57.72% | 28.70% | 39.32% | 37.68% | 42.77% |
| Cape Verde | 63.96% | 37.43% | 46.13% | 34.19% | 55.25% |
| Cape Verde Black | 63.96% | 37.43% | 46.13% | 34.19% | 55.25% |
| Central Africa | 53.76% | 28.34% | 35.58% | 31.89% | 41.17% |
| Central African Republic | 0.00% | 0.00% | 0.00% | 5.48% | 0.00% |
| Central African Republic Black | 0.00% | 0.00% | 0.00% | 5.48% | 0.00% |
| Central America | 2.19% | 1.40% | 1.40% | 2.18% | 1.40% |
| Chile | 79.15% | 51.76% | 51.46% | 17.11% | 59.19% |
| Chile Amerindian | 95.86% | 63.46% | 51.79% | 4.74% | 63.46% |
| Chile Mixed | 67.20% | 37.20% | 45.38% | 13.45% | 48.27% |
| China | 89.08% | 64.57% | 51.59% | 27.89% | 69.27% |
| China Oriental | 89.08% | 64.57% | 51.59% | 27.89% | 69.27% |
| Croatia | 69.59% | 42.24% | 34.39% | 17.55% | 61.20% |
| Croatia Caucasoid | 69.59% | 42.24% | 34.39% | 17.55% | 61.20% |
| Cuba | 57.50% | 31.74% | 46.26% | 28.21% | 52.25% |
| Cuba Caucasoid | 61.23% | 33.58% | 46.21% | 26.15% | 54.70% |
| Cuba Mulatto | 51.00% | 28.60% | 46.29% | 31.52% | 47.85% |
| Czech Republic | 68.27% | 51.89% | 50.40% | 17.60% | 69.70% |
| Czech Republic Caucasoid | 68.27% | 51.89% | 50.40% | 17.60% | 69.70% |
| East Africa | 46.60% | 27.59% | 42.14% | 30.39% | 39.64% |
| East Asia | 90.19% | 48.70% | 51.80% | 34.41% | 60.08% |
| Ecuador | 70.60% | 11.36% |  |  | 11.36% |
| Ecuador Amerindian | 70.60% | 11.36% |  |  | 11.36% |
| England | 73.42% | 53.80% | 69.63% | 25.16% | 79.85% |
| England Caucasoid | 80.20% | 56.89% | 75.08% | 30.22% | 81.21% |
| England Jew | 8.42% | 2.78% | 7.26% | 2.78% | 2.78% |
| Equatorial Guinea | 9.75% | 9.75% | 9.75% | 9.75% | 9.75% |
| Equatorial Guinea Black | 9.75% | 9.75% | 9.75% | 9.75% | 9.75% |
| Europe | 80.69% | 57.98% | 62.51% | 33.17% | 76.28% |
| Finland | 89.91% | 74.03% | 69.06% | 32.67% | 86.00% |
| Finland Caucasoid | 89.91% | 74.03% | 69.06% | 32.67% | 86.00% |
| France | 78.09% | 55.05% | 72.57% | 40.48% | 75.09% |
| France Caucasoid | 78.09% | 55.05% | 72.57% | 40.48% | 75.09% |
| Georgia | 82.99% | 56.95% | 49.19% | 38.32% | 68.28% |
| Georgia Caucasoid | 84.51% | 60.41% | 53.58% | 40.97% | 71.64% |
| Georgia Kurd | 82.12% | 51.28% | 32.45% | 37.29% | 63.64% |
| Germany | 83.80% | 59.13% | 68.43% | 32.90% | 79.56% |
| Germany Caucasoid | 83.80% | 59.13% | 68.43% | 32.90% | 79.56% |
| Guatemala | 2.19% | 1.40% | 1.40% | 2.18% | 1.40% |
| Guatemala Amerindian | 2.19% | 1.40% | 1.40% | 2.18% | 1.40% |
| Guinea-Bissau | 52.38% | 20.55% | 18.86% | 46.61% | 40.02% |
| Guinea-Bissau Black | 52.38% | 20.55% | 18.86% | 46.61% | 40.02% |
| Hong Kong | 82.40% | 63.85% | 48.92% | 14.71% | 64.67% |
| Hong Kong Oriental | 82.40% | 63.85% | 48.92% | 14.71% | 64.67% |
| India | 76.02% | 57.27% | 37.54% | 28.34% | 63.67% |
| India Asian | 76.02% | 57.27% | 37.54% | 28.34% | 63.67% |
| Indonesia | 69.24% | 49.54% | 32.30% | 11.85% | 51.30% |
| Indonesia Austronesian | 69.24% | 49.54% | 32.30% | 11.85% | 51.30% |
| Iran | 75.98% | 55.05% | 29.75% | 37.10% | 71.19% |
| Iran Persian | 75.98% | 55.05% | 29.75% | 37.10% | 71.19% |
| Ireland Northern | 78.77% | 56.15% | 73.69% | 23.29% | 82.24% |
| Ireland Northern Caucasoid | 78.77% | 56.15% | 73.69% | 23.29% | 82.24% |
| Ireland South | 75.93% | 50.85% | 74.30% | 25.20% | 82.28% |
| Ireland South Caucasoid | 75.93% | 50.85% | 74.30% | 25.20% | 82.28% |
| Israel | 56.83% | 36.55% | 37.77% | 22.42% | 43.87% |
| Israel Arab | 73.54% | 49.93% | 31.20% | 32.74% | 61.52% |
| Israel Jew | 53.01% | 34.82% | 48.61% | 19.73% | 39.22% |
| Italy | 74.72% | 52.87% | 69.27% | 45.99% | 69.53% |
| Italy Caucasoid | 74.72% | 52.87% | 69.27% | 45.99% | 69.53% |
| Ivory Coast |  |  | 4.54% | 4.54% | 13.30% |
| Ivory Coast Black |  |  | 4.54% | 4.54% | 13.30% |
| Japan | 92.28% | 49.93% | 51.73% | 34.91% | 61.75% |
| Japan Oriental | 92.28% | 49.93% | 51.73% | 34.91% | 61.75% |
| Jordan | 60.85% | 48.66% | 36.53% | 20.99% | 56.29% |
| Jordan Arab | 60.85% | 48.66% | 36.53% | 20.99% | 56.29% |
| Kenya | 40.22% | 23.03% | 38.88% | 31.73% | 34.89% |
| Kenya Black | 40.22% | 23.03% | 38.88% | 31.73% | 34.89% |
| Korea; South | 87.99% | 53.71% | 54.09% | 40.64% | 63.17% |
| Korea; South Oriental | 87.99% | 53.71% | 54.09% | 40.64% | 63.17% |
| Lebanon | 39.47% | 22.74% | 28.09% | 24.48% | 24.48% |
| Lebanon Mixed | 39.47% | 22.74% | 28.09% | 24.48% | 24.48% |
| Macedonia | 19.42% | 11.83% | 12.03% | 18.82% | 27.61% |
| Macedonia Caucasoid | 19.42% | 11.83% | 12.03% | 18.82% | 27.61% |
| Malaysia | 56.56% | 38.14% | 15.17% | 12.22% | 39.76% |
| Malaysia Austronesian | 47.81% | 42.85% | 39.16% | 26.04% | 42.85% |
| Malaysia Oriental | 60.39% | 39.40% | 11.60% | 11.54% | 41.20% |
| Mali | 75.24% | 43.02% | 25.57% | 49.48% | 52.97% |
| Mali Black | 75.24% | 43.02% | 25.57% | 49.48% | 52.97% |
| Martinique | 22.56% |  |  | 22.56% |  |
| Martinique Black | 22.56% |  |  | 22.56% |  |
| Mexico | 87.00% | 37.21% | 28.51% | 13.19% | 43.00% |
| Mexico Amerindian | 91.87% | 43.69% | 21.51% | 10.86% | 47.13% |
| Mexico Mestizo | 58.85% | 31.88% | 36.41% | 6.37% | 41.71% |
| Mongolia | 62.46% | 37.04% | 22.06% | 2.76% | 37.04% |
| Mongolia Oriental | 62.46% | 37.04% | 22.06% | 2.76% | 37.04% |
| Morocco | 59.38% | 36.93% | 57.82% | 30.11% | 53.92% |
| Morocco Arab | 54.73% | 30.32% | 59.02% | 26.38% | 49.87% |
| Morocco Caucasoid | 62.76% | 41.68% | 57.08% | 32.89% | 56.89% |
| New Caledonia | 96.59% | 38.01% | 34.54% | 12.83% | 42.04% |
| New Caledonia Melanesian | 96.59% | 38.01% | 34.54% | 12.83% | 42.04% |
| North Africa | 65.21% | 41.92% | 46.46% | 40.18% | 55.40% |
| North America | 77.75% | 48.47% | 52.74% | 28.72% | 62.76% |
| Northeast Asia | 89.43% | 65.04% | 53.09% | 28.23% | 69.43% |
| Oceania | 89.63% | 51.53% | 43.11% | 21.31% | 58.01% |
| Oman | 49.31% | 41.17% | 26.12% | 34.40% | 67.50% |
| Oman Arab | 49.31% | 41.17% | 26.12% | 34.40% | 67.50% |
| Pakistan | 82.09% | 65.45% | 32.24% | 26.13% | 71.75% |
| Pakistan Asian | 81.63% | 65.73% | 31.78% | 27.62% | 72.46% |
| Pakistan Mixed | 82.91% | 64.87% | 33.35% | 22.38% | 70.22% |
| Papua New Guinea | 98.68% | 68.01% | 32.34% | 23.07% | 68.07% |
| Papua New Guinea Melanesian | 98.68% | 68.01% | 32.34% | 23.07% | 68.07% |
| Peru | 70.27% | 37.12% | 10.20% | 1.99% | 38.87% |
| Peru Amerindian | 69.66% | 35.84% | 8.38% |  | 37.62% |
| Peru Mestizo | 1.99% | 1.99% | 1.99% | 1.99% | 1.99% |
| Philippines | 91.04% | 52.51% | 66.42% | 9.75% | 52.51% |
| Philippines Austronesian | 91.04% | 52.51% | 66.42% | 9.75% | 52.51% |
| Poland | 82.14% | 60.75% | 62.27% | 36.23% | 77.52% |
| Poland Caucasoid | 82.14% | 60.75% | 62.27% | 36.23% | 77.52% |
| Portugal | 71.43% | 46.68% | 58.28% | 35.86% | 62.92% |
| Portugal Caucasoid | 71.43% | 46.68% | 58.28% | 35.86% | 62.92% |
| Romania | 67.24% | 46.27% | 35.22% | 16.65% | 63.05% |
| Romania Caucasoid | 67.24% | 46.27% | 35.22% | 16.65% | 63.05% |
| Russia | 87.78% | 55.97% | 47.43% | 37.86% | 67.08% |
| Russia Caucasoid | 59.04% | 19.00% | 22.21% | 19.00% | 19.00% |
| Russia Mixed | 58.45% | 31.49% | 34.95% | 31.49% | 31.49% |
| Russia Other | 89.23% | 56.52% | 51.22% | 31.69% | 72.74% |
| Russia Siberian | 87.26% | 58.43% | 48.80% | 40.38% | 67.41% |
| Rwanda | 13.51% | 0.40% |  | 13.51% | 2.97% |
| Rwanda Black | 13.51% | 0.40% |  | 13.51% | 2.97% |
| Sao Tome and Principe | 48.81% | 27.29% | 18.21% | 28.24% | 46.59% |
| Sao Tome and Principe Black | 48.81% | 27.29% | 18.21% | 28.24% | 46.59% |
| Saudi Arabia | 76.36% | 50.59% | 37.75% | 32.32% | 68.10% |
| Saudi Arabia Arab | 76.36% | 50.59% | 37.75% | 32.32% | 68.10% |
| Scotland | 31.78% | 35.30% | 31.00% | 33.34% | 52.35% |
| Scotland Caucasoid | 31.78% | 35.30% | 31.00% | 33.34% | 52.35% |
| Senegal | 61.73% | 30.47% | 39.92% | 44.84% | 45.82% |
| Senegal Black | 61.73% | 30.47% | 39.92% | 44.84% | 45.82% |
| Serbia | 7.65% |  | 15.91% | 1.00% | 22.03% |
| Serbia Caucasoid | 7.65% |  | 15.91% | 1.00% | 22.03% |
| Singapore | 83.94% | 53.53% | 42.83% | 21.92% | 57.96% |
| Singapore Austronesian | 81.66% | 49.71% | 43.28% | 19.26% | 54.75% |
| Singapore Oriental | 87.01% | 57.91% | 44.22% | 24.30% | 61.93% |
| South Africa | 76.85% | 45.87% | 53.37% | 34.45% | 59.59% |
| South Africa Black | 50.79% | 34.39% | 44.94% | 29.44% | 52.28% |
| South Africa Other | 85.98% | 67.56% | 44.41% | 19.11% | 74.21% |
| South America | 70.41% | 44.57% | 33.31% | 16.65% | 50.77% |
| South Asia | 80.52% | 61.13% | 40.90% | 31.08% | 69.04% |
| Southeast Asia | 88.99% | 52.16% | 46.96% | 25.49% | 55.43% |
| Southwest Asia | 63.67% | 43.23% | 36.21% | 27.10% | 56.33% |
| Spain | 52.35% | 35.19% | 50.86% | 34.24% | 49.96% |
| Spain Caucasoid | 52.35% | 35.19% | 50.86% | 34.24% | 49.96% |
| Sri Lanka | 23.96% | 19.72% | 20.61% |  | 19.72% |
| Sri Lanka Asian | 23.96% | 19.72% | 20.61% |  | 19.72% |
| Sudan | 66.72% | 48.77% | 45.45% | 42.09% | 61.04% |
| Sudan Arab | 44.13% | 21.08% | 7.30% | 17.93% | 33.92% |
| Sudan Mixed | 66.20% | 51.06% | 48.18% | 43.77% | 63.25% |
| Sweden | 83.85% | 63.22% | 55.25% | 3.27% | 79.92% |
| Sweden Caucasoid | 83.85% | 63.22% | 55.25% | 3.27% | 79.92% |
| Switzerland | 59.55% | 22.38% | 28.94% | 28.94% | 28.94% |
| Switzerland Caucasoid | 59.55% | 22.38% | 28.94% | 28.94% | 28.94% |
| Taiwan | 92.93% | 53.71% | 55.84% | 30.11% | 56.25% |
| Taiwan Oriental | 92.93% | 53.71% | 55.84% | 30.11% | 56.25% |
| Thailand | 82.67% | 64.60% | 43.20% | 26.36% | 70.15% |
| Thailand Oriental | 82.67% | 64.60% | 43.20% | 26.36% | 70.15% |
| Tunisia | 68.35% | 43.00% | 56.11% | 41.00% | 56.38% |
| Tunisia Arab | 68.35% | 43.00% | 56.11% | 41.00% | 56.38% |
| Turkey | 29.10% | 18.64% | 18.64% | 18.64% | 18.64% |
| Turkey Caucasoid | 29.10% | 18.64% | 18.64% | 18.64% | 18.64% |
| Uganda | 59.30% | 37.79% | 48.63% | 32.89% | 51.45% |
| Uganda Black | 59.30% | 37.79% | 48.63% | 32.89% | 51.45% |
| United Arab Emirates | 3.37% | 1.20% | 6.88% |  | 1.20% |
| United Arab Emirates Arab | 3.37% | 1.20% | 6.88% |  | 1.20% |
| United Kingdom | 46.12% | 15.18% | 35.36% | 17.19% | 17.19% |
| United Kingdom Caucasoid | 46.12% | 15.18% | 35.36% | 17.19% | 17.19% |
| United States | 77.72% | 48.71% | 53.26% | 29.19% | 63.26% |
| United States Amerindian | 86.36% | 36.47% | 30.00% | 11.82% | 42.05% |
| United States Asian | 89.70% | 58.66% | 51.28% | 34.01% | 67.14% |
| United States Black | 66.68% | 42.33% | 43.75% | 34.23% | 57.56% |
| United States Caucasoid | 80.72% | 56.18% | 69.78% | 30.12% | 76.19% |
| United States Hispanic | 79.91% | 50.39% | 52.24% | 28.29% | 63.35% |
| United States Mestizo | 81.15% | 48.77% | 55.86% | 26.55% | 61.46% |
| United States Polynesian | 96.28% | 58.16% | 67.06% | 49.59% | 66.38% |
| Venezuela | 85.08% |  | 12.81% | 6.90% | 1.42% |
| Venezuela Amerindian | 84.51% |  | 12.92% |  |  |
| Venezuela Caucasoid | 9.18% |  |  | 11.45% | 2.39% |
| Venezuela Mestizo | 7.84% |  |  | 9.75% | 1.99% |
| Vietnam | 88.09% | 63.85% | 54.92% | 26.76% | 66.06% |
| Vietnam Oriental | 88.09% | 63.85% | 54.92% | 26.76% | 66.06% |
| West Africa | 61.74% | 30.81% | 36.30% | 40.28% | 45.15% |
| West Indies | 58.98% | 31.35% | 45.93% | 30.58% | 51.90% |
| World | 80.37% | 54.43% | 55.92% | 31.04% | 68.10% |
| Zambia | 58.04% | 43.63% | 63.12% | 19.18% | 52.29% |
| Zambia Black | 58.04% | 43.63% | 63.12% | 19.18% | 52.29% |
| Zimbabwe | 53.46% | 28.04% | 48.34% | 33.54% | 40.46% |
| Zimbabwe Black | 53.46% | 28.04% | 48.34% | 33.54% | 40.46% |
