# Supplementary material for: Computational perspectives revealed prospective vaccine candidates from five structural proteins of novel SARS corona virus 2019 (SARS-CoV-2)
Source: PeerJ. 2020 Sep 29;8:e9855. doi: 10.7717/peerj.9855 (PMC7531350; doi:10.7717/peerj.9855)
Supplement: Supplemental Information 7 [file peerj-08-9855-s007.docx]

Supplementary Table: S5 Predicted MHC II alleles and the corresponding epitopes

| **urface glycoprotein** | | | | | |
| --- | --- | --- | --- | --- | --- |
| **No** | **Core sequence** | **MHC II alleles** | **position** | **Peptide** | **IC50 value** |
|  | AEIRASANL | HLA-DRB1*15:01 | 1012-1026 | LIRAAEIRASANLAA | 768 |
|  | AEIRASANL | HLA-DRB1*13:02 | 1011-1025 | QLIRAAEIRASANLA | 962 |
|  | AEIRASANL | HLA-DRB1*15:01 | 1011-1025 | QLIRAAEIRASANLA | 1268 |
|  | AEIRASANL | HLA-DRB1*07:01 | 1011-1025 | QLIRAAEIRASANLA | 1566 |
|  | AEIRASANL | HLA-DQA1*04:01/DQB1*04:02 | 1013-1027 | IRAAEIRASANLAAT | 1690 |
|  | FLHVTYVPA | HLA-DRB1*01:01 | 1059-1073 | GVVFLHVTYVPAQEK | 90 |
|  | FLHVTYVPA | HLA-DRB1*01:01 | 1060-1074 | VVFLHVTYVPAQEKN | 94 |
|  | FLHVTYVPA | HLA-DPA1*01:03/DPB1*02:01 | 1057-1071 | PHGVVFLHVTYVPAQ | 98 |
|  | FLHVTYVPA | HLA-DPA1*01:03/DPB1*02:01 | 1058-1072 | HGVVFLHVTYVPAQE | 102 |
|  | FLHVTYVPA | HLA-DPA1*01:03/DPB1*02:01 | 1059-1073 | GVVFLHVTYVPAQEK | 102 |
|  | FLHVTYVPA | HLA-DPA1*01:03/DPB1*02:01 | 1060-1074 | VVFLHVTYVPAQEKN | 106 |
|  | FLHVTYVPA | HLA-DRB1*04:05 | 1058-1072 | HGVVFLHVTYVPAQE | 200 |
|  | FLHVTYVPA | HLA-DRB1*01:01 | 1061-1075 | VFLHVTYVPAQEKNF | 203 |
|  | FLHVTYVPA | HLA-DRB1*01:01 | 1062-1076 | FLHVTYVPAQEKNFT | 206 |
|  | FLHVTYVPA | HLA-DRB1*04:05 | 1057-1071 | PHGVVFLHVTYVPAQ | 208 |
|  | FLHVTYVPA | HLA-DRB1*04:05 | 1059-1073 | GVVFLHVTYVPAQEK | 214 |
|  | FLHVTYVPA | HLA-DRB1*04:05 | 1060-1074 | VVFLHVTYVPAQEKN | 217 |
|  | FLHVTYVPA | HLA-DRB5*01:01 | 1059-1073 | GVVFLHVTYVPAQEK | 230 |
|  | FLHVTYVPA | HLA-DPA1*01/DPB1*04:01 1 | 1057-1071 | PHGVVFLHVTYVPAQ | 237 |
|  | FLHVTYVPA | HLA-DRB1*04:01 | 1059-1073 | GVVFLHVTYVPAQEK | 256 |
|  | FLHVTYVPA | HLA-DRB1*04:01 | 1057-1071 | PHGVVFLHVTYVPAQ | 264 |
|  | FLHVTYVPA | HLA-DRB1*04:01 | 1058-1072 | HGVVFLHVTYVPAQE | 264 |
|  | FLHVTYVPA | HLA-DPA1*01/DPB1*04:01 1 | 1058-1072 | HGVVFLHVTYVPAQE | 274 |
|  | FLHVTYVPA | HLA-DPA1*01/DPB1*04:01 1 | 1059-1073 | GVVFLHVTYVPAQEK | 277 |
|  | FLHVTYVPA | HLA-DRB1*04:01 | 1060-1074 | VVFLHVTYVPAQEKN | 279 |
|  | FLHVTYVPA | HLA-DPA1*01/DPB1*04:01 1 | 1060-1074 | VVFLHVTYVPAQEKN | 300 |
|  | FLHVTYVPA | HLA-DPA1*01:03/DPB1*02:01 | 1061-1075 | VFLHVTYVPAQEKNF | 310 |
|  | FLHVTYVPA | HLA-DPA1*01:03/DPB1*02:01 | 1062-1076 | FLHVTYVPAQEKNFT | 336 |
|  | FLHVTYVPA | HLA-DPA1*03:01/DPB1*04:02 | 1057-1071 | PHGVVFLHVTYVPAQ | 479 |
|  | FLHVTYVPA | HLA-DRB1*11:01 | 1059-1073 | GVVFLHVTYVPAQEK | 485 |
|  | FLHVTYVPA | HLA-DRB1*11:01 | 1060-1074 | VVFLHVTYVPAQEKN | 506 |
|  | FLHVTYVPA | HLA-DRB1*04:05 | 1061-1075 | VFLHVTYVPAQEKNF | 522 |
|  | FLHVTYVPA | HLA-DPA1*02:01/DPB1*01:01 | 1057-1071 | PHGVVFLHVTYVPAQ | 543 |
|  | FLHVTYVPA | HLA-DPA1*02:01/DPB1*01:01 | 1059-1073 | GVVFLHVTYVPAQEK | 574 |
|  | FLHVTYVPA | HLA-DRB1*04:05 | 1062-1076 | FLHVTYVPAQEKNFT | 576 |
|  | FLHVTYVPA | HLA-DPA1*02:01/DPB1*01:01 | 1058-1072 | HGVVFLHVTYVPAQE | 577 |
|  | FLHVTYVPA | HLA-DPA1*02:01/DPB1*01:01 | 1060-1074 | VVFLHVTYVPAQEKN | 603 |
|  | FLHVTYVPA | HLA-DPA1*03:01/DPB1*04:02 | 1058-1072 | HGVVFLHVTYVPAQE | 610 |
|  | FLHVTYVPA | HLA-DRB1*04:01 | 1061-1075 | VFLHVTYVPAQEKNF | 628 |
|  | FLHVTYVPA | HLA-DRB1*08:02 | 1059-1073 | GVVFLHVTYVPAQEK | 689 |
|  | FLHVTYVPA | HLA-DRB1*08:02 | 1060-1074 | VVFLHVTYVPAQEKN | 704 |
|  | FLHVTYVPA | HLA-DRB1*08:02 | 1058-1072 | HGVVFLHVTYVPAQE | 706 |
|  | FLHVTYVPA | HLA-DRB1*12:01 | 1057-1071 | PHGVVFLHVTYVPAQ | 756 |
|  | FLHVTYVPA | HLA-DRB1*12:01 | 1058-1072 | HGVVFLHVTYVPAQE | 770 |
|  | FLHVTYVPA | HLA-DPA1*03:01/DPB1*04:02 | 1060-1074 | VVFLHVTYVPAQEKN | 778 |
|  | FLHVTYVPA | HLA-DRB1*04:01 | 1062-1076 | FLHVTYVPAQEKNFT | 784 |
|  | FLHVTYVPA | HLA-DRB1*08:02 | 1057-1071 | PHGVVFLHVTYVPAQ | 787 |
|  | FLHVTYVPA | HLA-DPA1*03:01/DPB1*04:02 | 1059-1073 | GVVFLHVTYVPAQEK | 787 |
|  | FLHVTYVPA | HLA-DPA1*01/DPB1*04:01 1 | 1061-1075 | VFLHVTYVPAQEKNF | 813 |
|  | FLHVTYVPA | HLA-DRB1*12:01 | 1059-1073 | GVVFLHVTYVPAQEK | 904 |
|  | FLHVTYVPA | HLA-DPA1*01/DPB1*04:01 1 | 1062-1076 | FLHVTYVPAQEKNFT | 964 |
|  | FLHVTYVPA | HLA-DRB1*11:01 | 1061-1075 | VFLHVTYVPAQEKNF | 973 |
|  | FLHVTYVPA | HLA-DRB1*09:01 | 1059-1073 | GVVFLHVTYVPAQEK | 1005 |
|  | FLHVTYVPA | HLA-DRB1*09:01 | 1060-1074 | VVFLHVTYVPAQEKN | 1044 |
|  | FLHVTYVPA | HLA-DRB1*08:02 | 1061-1075 | VFLHVTYVPAQEKNF | 1128 |
|  | FLHVTYVPA | HLA-DQA1*01:02/DQB1*06:02 | 1057-1071 | PHGVVFLHVTYVPAQ | 1155 |
|  | FLHVTYVPA | HLA-DRB1*11:01 | 1062-1076 | FLHVTYVPAQEKNFT | 1166 |
|  | FLHVTYVPA | HLA-DRB1*12:01 | 1060-1074 | VVFLHVTYVPAQEKN | 1170 |
|  | FLHVTYVPA | HLA-DPA1*02:01/DPB1*01:01 | 1061-1075 | VFLHVTYVPAQEKNF | 1175 |
|  | FLHVTYVPA | HLA-DQA1*05:01/DQB1*03:01 | 1057-1071 | PHGVVFLHVTYVPAQ | 1258 |
|  | FLHVTYVPA | HLA-DPA1*02:01/DPB1*01:01 | 1062-1076 | FLHVTYVPAQEKNFT | 1296 |
|  | FLHVTYVPA | HLA-DRB1*12:01 | 1061-1075 | VFLHVTYVPAQEKNF | 1719 |
|  | FLHVTYVPA | HLA-DPA1*03:01/DPB1*04:02 | 1061-1075 | VFLHVTYVPAQEKNF | 2067 |
|  | FLHVTYVPA | HLA-DPA1*03:01/DPB1*04:02 | 1062-1076 | FLHVTYVPAQEKNFT | 2437 |
|  | FLHVTYVPA | HLA-DQA1*03:01/DQB1*03:02 | 1058-1072 | HGVVFLHVTYVPAQE | 2587 |
|  | FLHVTYVPA | HLA-DRB1*12:01 | 1062-1076 | FLHVTYVPAQEKNFT | 2803 |
|  | FLHVTYVPA | HLA-DQA1*05:01/DQB1*02:01 | 1058-1072 | HGVVFLHVTYVPAQE | 2949 |
|  | FQPTNGVGY | HLA-DRB1*07:01 | 492-506 | LQSYGFQPTNGVGYQ | 307 |
|  | FQPTNGVGY | HLA-DRB1*07:01 | 493-507 | QSYGFQPTNGVGYQP | 321 |
|  | FQPTNGVGY | HLA-DRB1*07:01 | 494-508 | SYGFQPTNGVGYQPY | 346 |
|  | FQPTNGVGY | HLA-DRB1*07:01 | 495-509 | YGFQPTNGVGYQPYR | 353 |
|  | FQPTNGVGY | HLA-DRB1*09:01 | 494-508 | SYGFQPTNGVGYQPY | 569 |
|  | FQPTNGVGY | HLA-DRB1*09:01 | 495-509 | YGFQPTNGVGYQPYR | 581 |
|  | FQPTNGVGY | HLA-DRB1*01:01 | 496-510 | GFQPTNGVGYQPYRV | 726 |
|  | FQPTNGVGY | HLA-DRB1*07:01 | 497-511 | FQPTNGVGYQPYRVV | 831 |
|  | FQPTNGVGY | HLA-DRB1*07:01 | 496-510 | GFQPTNGVGYQPYRV | 859 |
|  | FQPTNGVGY | HLA-DRB5*01:01 | 494-508 | SYGFQPTNGVGYQPY | 1201 |
|  | FQPTNGVGY | HLA-DRB5*01:01 | 495-509 | YGFQPTNGVGYQPYR | 1224 |
|  | FQPTNGVGY | HLA-DRB1*09:01 | 497-511 | FQPTNGVGYQPYRVV | 1278 |
|  | FQPTNGVGY | HLA-DRB1*13:02 | 492-506 | LQSYGFQPTNGVGYQ | 1413 |
|  | FQPTNGVGY | HLA-DRB1*13:02 | 493-507 | QSYGFQPTNGVGYQP | 1474 |
|  | FQPTNGVGY | HLA-DRB1*12:01 | 493-507 | QSYGFQPTNGVGYQP | 1541 |
|  | FQPTNGVGY | HLA-DRB1*12:01 | 492-506 | LQSYGFQPTNGVGYQ | 1551 |
|  | FQPTNGVGY | HLA-DRB1*13:02 | 494-508 | SYGFQPTNGVGYQPY | 1612 |
|  | FQPTNGVGY | HLA-DRB1*13:02 | 495-509 | YGFQPTNGVGYQPYR | 1634 |
|  | FQPTNGVGY | HLA-DRB1*09:01 | 496-510 | GFQPTNGVGYQPYRV | 1810 |
|  | FQPTNGVGY | HLA-DRB3*01:01 | 492-506 | LQSYGFQPTNGVGYQ | 1874 |
|  | FQPTNGVGY | HLA-DRB3*01:01 | 493-507 | QSYGFQPTNGVGYQP | 1919 |
|  | FQPTNGVGY | HLA-DRB1*12:01 | 494-508 | SYGFQPTNGVGYQPY | 2201 |
|  | FQPTNGVGY | HLA-DRB1*12:01 | 495-509 | YGFQPTNGVGYQPYR | 2314 |
|  | FQPTNGVGY | HLA-DRB3*01:01 | 494-508 | SYGFQPTNGVGYQPY | 2322 |
|  | FQPTNGVGY | HLA-DRB3*01:01 | 495-509 | YGFQPTNGVGYQPYR | 2382 |
|  | FQPTNGVGY | HLA-DRB1*04:04 | 494-508 | SYGFQPTNGVGYQPY | 2973 |
|  | FVSNGTHWF | HLA-DRB3*01:01 | 1090-1104 | PREGVFVSNGTHWFV | 67 |
|  | FVSNGTHWF | HLA-DRB3*01:01 | 1091-1105 | REGVFVSNGTHWFVT | 67 |
|  | FVSNGTHWF | HLA-DRB3*01:01 | 1092-1106 | EGVFVSNGTHWFVTQ | 67 |
|  | FVSNGTHWF | HLA-DRB3*01:01 | 1093-1107 | GVFVSNGTHWFVTQR | 68 |
|  | FVSNGTHWF | HLA-DRB3*01:01 | 1094-1108 | VFVSNGTHWFVTQRN | 212 |
|  | FVSNGTHWF | HLA-DRB3*01:01 | 1095-1109 | FVSNGTHWFVTQRNF | 221 |
|  | FVSNGTHWF | HLA-DRB1*01:01 | 1090-1104 | PREGVFVSNGTHWFV | 424 |
|  | FVSNGTHWF | HLA-DRB1*01:01 | 1091-1105 | REGVFVSNGTHWFVT | 437 |
|  | FVSNGTHWF | HLA-DRB1*01:01 | 1092-1106 | EGVFVSNGTHWFVTQ | 474 |
|  | FVSNGTHWF | HLA-DRB1*01:01 | 1093-1107 | GVFVSNGTHWFVTQR | 545 |
|  | FVSNGTHWF | HLA-DRB1*07:01 | 1090-1104 | PREGVFVSNGTHWFV | 605 |
|  | FVSNGTHWF | HLA-DRB1*07:01 | 1091-1105 | REGVFVSNGTHWFVT | 608 |
|  | FVSNGTHWF | HLA-DRB1*07:01 | 1092-1106 | EGVFVSNGTHWFVTQ | 630 |
|  | FVSNGTHWF | HLA-DRB1*07:01 | 1093-1107 | GVFVSNGTHWFVTQR | 790 |
|  | FVSNGTHWF | HLA-DRB1*04:05 | 1094-1108 | VFVSNGTHWFVTQRN | 856 |
|  | FVSNGTHWF | HLA-DRB1*04:01 | 1090-1104 | PREGVFVSNGTHWFV | 858 |
|  | FVSNGTHWF | HLA-DRB1*13:02 | 1093-1107 | GVFVSNGTHWFVTQR | 921 |
|  | FVSNGTHWF | HLA-DRB1*04:01 | 1091-1105 | REGVFVSNGTHWFVT | 963 |
|  | FVSNGTHWF | HLA-DRB1*04:01 | 1092-1106 | EGVFVSNGTHWFVTQ | 989 |
|  | FVSNGTHWF | HLA-DRB1*07:01 | 1095-1109 | FVSNGTHWFVTQRNF | 1064 |
|  | FVSNGTHWF | HLA-DRB1*01:01 | 1094-1108 | VFVSNGTHWFVTQRN | 1082 |
|  | FVSNGTHWF | HLA-DRB1*01:01 | 1095-1109 | FVSNGTHWFVTQRNF | 1202 |
|  | FVSNGTHWF | HLA-DRB5*01:01 | 1091-1105 | REGVFVSNGTHWFVT | 1252 |
|  | FVSNGTHWF | HLA-DRB5*01:01 | 1090-1104 | PREGVFVSNGTHWFV | 1262 |
|  | FVSNGTHWF | HLA-DRB1*04:01 | 1093-1107 | GVFVSNGTHWFVTQR | 1281 |
|  | FVSNGTHWF | HLA-DRB1*07:01 | 1094-1108 | VFVSNGTHWFVTQRN | 1281 |
|  | FVSNGTHWF | HLA-DRB1*09:01 | 1090-1104 | PREGVFVSNGTHWFV | 1396 |
|  | FVSNGTHWF | HLA-DRB5*01:01 | 1092-1106 | EGVFVSNGTHWFVTQ | 1439 |
|  | FVSNGTHWF | HLA-DRB1*09:01 | 1092-1106 | EGVFVSNGTHWFVTQ | 1489 |
|  | FVSNGTHWF | HLA-DRB1*09:01 | 1091-1105 | REGVFVSNGTHWFVT | 1493 |
|  | FVSNGTHWF | HLA-DRB5*01:01 | 1093-1107 | GVFVSNGTHWFVTQR | 1634 |
|  | FVSNGTHWF | HLA-DRB1*09:01 | 1093-1107 | GVFVSNGTHWFVTQR | 1726 |
|  | FVSNGTHWF | HLA-DRB1*04:01 | 1094-1108 | VFVSNGTHWFVTQRN | 2123 |
|  | FVSNGTHWF | HLA-DRB1*11:01 | 1093-1107 | GVFVSNGTHWFVTQR | 2578 |
|  | FVSNGTHWF | HLA-DRB1*03:01 | 1092-1106 | EGVFVSNGTHWFVTQ | 2596 |
|  | FVSNGTHWF | HLA-DRB1*03:01 | 1091-1105 | REGVFVSNGTHWFVT | 2609 |
|  | FVSNGTHWF | HLA-DRB1*03:01 | 1090-1104 | PREGVFVSNGTHWFV | 2639 |
|  | FVSNGTHWF | HLA-DRB1*03:01 | 1093-1107 | GVFVSNGTHWFVTQR | 2688 |
|  | GQTGKIADY | HLA-DQA1*05:01/DQB1*03:01 | 410-424 | IAPGQTGKIADYNYK | 322 |
|  | GQTGKIADY | HLA-DQA1*05:01/DQB1*03:01 | 411-425 | APGQTGKIADYNYKL | 489 |
|  | GQTGKIADY | HLA-DQA1*05:01/DQB1*03:01 | 412-426 | PGQTGKIADYNYKLP | 1406 |
|  | GQTGKIADY | HLA-DQA1*05:01/DQB1*03:01 | 413-427 | GQTGKIADYNYKLPD | 1978 |
|  | GTITSGWTF | HLA-DRB1*07:01 | 880-894 | GTITSGWTFGAGAAL | 291 |
|  | GTITSGWTF | HLA-DRB1*07:01 | 875-889 | SALLAGTITSGWTFG | 419 |
|  | GTITSGWTF | HLA-DRB1*07:01 | 876-890 | ALLAGTITSGWTFGA | 423 |
|  | GTITSGWTF | HLA-DRB1*07:01 | 877-891 | LLAGTITSGWTFGAG | 525 |
|  | GTITSGWTF | HLA-DRB1*07:01 | 878-892 | LAGTITSGWTFGAGA | 565 |
|  | GTITSGWTF | HLA-DRB1*07:01 | 879-893 | AGTITSGWTFGAGAA | 1773 |
|  | GTITSGWTF | HLA-DRB1*15:01 | 878-892 | LAGTITSGWTFGAGA | 2589 |
|  | GTITSGWTF | HLA-DPA1*01:03/DPB1*02:01 | 877-891 | LLAGTITSGWTFGAG | 2778 |
|  | GVYFASTEK | HLA-DRB5*01:01 | 87-101 | NDGVYFASTEKSNII | 488 |
|  | GVYFASTEK | HLA-DRB5*01:01 | 86-100 | FNDGVYFASTEKSNI | 492 |
|  | GVYFASTEK | HLA-DRB5*01:01 | 85-99 | PFNDGVYFASTEKSN | 685 |
|  | GVYFASTEK | HLA-DRB5*01:01 | 84-98 | LPFNDGVYFASTEKS | 694 |
|  | GVYFASTEK | HLA-DRB1*11:01 | 84-98 | LPFNDGVYFASTEKS | 1292 |
|  | GVYFASTEK | HLA-DRB1*15:01 | 87-101 | NDGVYFASTEKSNII | 1388 |
|  | GVYFASTEK | HLA-DRB1*07:01 | 84-98 | LPFNDGVYFASTEKS | 1703 |
|  | GVYFASTEK | HLA-DRB1*04:05 | 85-99 | PFNDGVYFASTEKSN | 1779 |
|  | GVYFASTEK | HLA-DRB1*04:04 | 87-101 | NDGVYFASTEKSNII | 1894 |
|  | GVYFASTEK | HLA-DRB1*15:01 | 86-100 | FNDGVYFASTEKSNI | 1940 |
|  | GVYFASTEK | HLA-DRB1*04:04 | 86-100 | FNDGVYFASTEKSNI | 2026 |
|  | GVYFASTEK | HLA-DRB1*15:01 | 84-98 | LPFNDGVYFASTEKS | 2062 |
|  | GVYFASTEK | HLA-DRB1*15:01 | 85-99 | PFNDGVYFASTEKSN | 2144 |
|  | GVYFASTEK | HLA-DRB1*04:04 | 85-99 | PFNDGVYFASTEKSN | 2404 |
|  | GVYFASTEK | HLA-DRB1*04:05 | 84-98 | LPFNDGVYFASTEKS | 2462 |
|  | GVYFASTEK | HLA-DRB1*04:04 | 84-98 | LPFNDGVYFASTEKS | 2537 |
|  | IAIPTNFTI | HLA-DRB1*09:01 | 712-726 | IAIPTNFTISVTTEI | 136 |
|  | IAIPTNFTI | HLA-DRB1*13:02 | 708-722 | SNNSIAIPTNFTISV | 186 |
|  | IAIPTNFTI | HLA-DRB1*07:01 | 710-724 | NSIAIPTNFTISVTT | 213 |
|  | IAIPTNFTI | HLA-DRB1*01:01 | 710-724 | NSIAIPTNFTISVTT | 402 |
|  | IAIPTNFTI | HLA-DRB1*07:01 | 708-722 | SNNSIAIPTNFTISV | 431 |
|  | IAIPTNFTI | HLA-DRB1*07:01 | 707-721 | YSNNSIAIPTNFTIS | 453 |
|  | IAIPTNFTI | HLA-DRB1*07:01 | 709-723 | NNSIAIPTNFTISVT | 498 |
|  | IAIPTNFTI | HLA-DPA1*02:01/DPB1*01:01 | 710-724 | NSIAIPTNFTISVTT | 626 |
|  | IAIPTNFTI | HLA-DPA1*02:01/DPB1*01:01 | 709-723 | NNSIAIPTNFTISVT | 630 |
|  | IAIPTNFTI | HLA-DPA1*02:01/DPB1*01:01 | 708-722 | SNNSIAIPTNFTISV | 643 |
|  | IAIPTNFTI | HLA-DPA1*02:01/DPB1*01:01 | 707-721 | YSNNSIAIPTNFTIS | 715 |
|  | IAIPTNFTI | HLA-DRB5*01:01 | 712-726 | IAIPTNFTISVTTEI | 833 |
|  | IAIPTNFTI | HLA-DPA1*02:01/DPB1*01:01 | 712-726 | IAIPTNFTISVTTEI | 900 |
|  | IAIPTNFTI | HLA-DRB1*04:01 | 710-724 | NSIAIPTNFTISVTT | 946 |
|  | IAIPTNFTI | HLA-DRB1*13:02 | 707-721 | YSNNSIAIPTNFTIS | 957 |
|  | IAIPTNFTI | HLA-DRB1*04:01 | 707-721 | YSNNSIAIPTNFTIS | 971 |
|  | IAIPTNFTI | HLA-DRB1*04:01 | 708-722 | SNNSIAIPTNFTISV | 978 |
|  | IAIPTNFTI | HLA-DRB1*04:01 | 709-723 | NNSIAIPTNFTISVT | 979 |
|  | IAIPTNFTI | HLA-DRB1*15:01 | 710-724 | NSIAIPTNFTISVTT | 1030 |
|  | IAIPTNFTI | HLA-DRB1*04:04 | 710-724 | NSIAIPTNFTISVTT | 1141 |
|  | IAIPTNFTI | HLA-DPA1*03:01/DPB1*04:02 | 710-724 | NSIAIPTNFTISVTT | 1197 |
|  | IAIPTNFTI | HLA-DPA1*02:01/DPB1*01:01 | 711-725 | SIAIPTNFTISVTTE | 1201 |
|  | IAIPTNFTI | HLA-DPA1*03:01/DPB1*04:02 | 709-723 | NNSIAIPTNFTISVT | 1209 |
|  | IAIPTNFTI | HLA-DPA1*03:01/DPB1*04:02 | 708-722 | SNNSIAIPTNFTISV | 1228 |
|  | IAIPTNFTI | HLA-DPA1*01:03/DPB1*02:01 | 710-724 | NSIAIPTNFTISVTT | 1377 |
|  | IAIPTNFTI | HLA-DPA1*01:03/DPB1*02:01 | 709-723 | NNSIAIPTNFTISVT | 1379 |
|  | IAIPTNFTI | HLA-DPA1*01:03/DPB1*02:01 | 708-722 | SNNSIAIPTNFTISV | 1384 |
|  | IAIPTNFTI | HLA-DPA1*01:03/DPB1*02:01 | 712-726 | IAIPTNFTISVTTEI | 1411 |
|  | IAIPTNFTI | HLA-DPA1*01:03/DPB1*02:01 | 707-721 | YSNNSIAIPTNFTIS | 1426 |
|  | IAIPTNFTI | HLA-DPA1*03:01/DPB1*04:02 | 707-721 | YSNNSIAIPTNFTIS | 1629 |
|  | IAIPTNFTI | HLA-DRB3*01:01 | 708-722 | SNNSIAIPTNFTISV | 1766 |
|  | IAIPTNFTI | HLA-DRB5*01:01 | 708-722 | SNNSIAIPTNFTISV | 2011 |
|  | IAIPTNFTI | HLA-DRB5*01:01 | 709-723 | NNSIAIPTNFTISVT | 2034 |
|  | IAIPTNFTI | HLA-DRB5*01:01 | 707-721 | YSNNSIAIPTNFTIS | 2097 |
|  | IAIPTNFTI | HLA-DRB5*01:01 | 710-724 | NSIAIPTNFTISVTT | 2239 |
|  | IAIPTNFTI | HLA-DRB1*12:01 | 708-722 | SNNSIAIPTNFTISV | 2746 |
|  | IAIPTNFTI | HLA-DQA1*04:01/DQB1*04:02 | 709-723 | NNSIAIPTNFTISVT | 2769 |
|  | IAIPTNFTI | HLA-DRB1*12:01 | 709-723 | NNSIAIPTNFTISVT | 2781 |
|  | IPTNFTISV | HLA-DRB1*13:02 | 709-723 | NNSIAIPTNFTISVT | 185 |
|  | IPTNFTISV | HLA-DRB1*13:02 | 710-724 | NSIAIPTNFTISVTT | 185 |
|  | IPTNFTISV | HLA-DRB1*13:02 | 711-725 | SIAIPTNFTISVTTE | 188 |
|  | IPTNFTISV | HLA-DRB1*13:02 | 712-726 | IAIPTNFTISVTTEI | 188 |
|  | IPTNFTISV | HLA-DRB1*01:01 | 712-726 | IAIPTNFTISVTTEI | 226 |
|  | IPTNFTISV | HLA-DRB1*13:02 | 713-727 | AIPTNFTISVTTEIL | 470 |
|  | IPTNFTISV | HLA-DRB1*13:02 | 714-728 | IPTNFTISVTTEILP | 481 |
|  | IPTNFTISV | HLA-DRB1*08:02 | 709-723 | NNSIAIPTNFTISVT | 721 |
|  | IPTNFTISV | HLA-DRB1*08:02 | 710-724 | NSIAIPTNFTISVTT | 880 |
|  | IPTNFTISV | HLA-DRB3*01:01 | 712-726 | IAIPTNFTISVTTEI | 951 |
|  | IPTNFTISV | HLA-DRB1*08:02 | 711-725 | SIAIPTNFTISVTTE | 967 |
|  | IPTNFTISV | HLA-DRB1*08:02 | 712-726 | IAIPTNFTISVTTEI | 971 |
|  | IPTNFTISV | HLA-DRB1*03:01 | 712-726 | IAIPTNFTISVTTEI | 1052 |
|  | IPTNFTISV | HLA-DPA1*03:01/DPB1*04:02 | 712-726 | IAIPTNFTISVTTEI | 1342 |
|  | IPTNFTISV | HLA-DRB1*08:02 | 714-728 | IPTNFTISVTTEILP | 1733 |
|  | IPTNFTISV | HLA-DRB3*01:01 | 709-723 | NNSIAIPTNFTISVT | 1769 |
|  | IPTNFTISV | HLA-DRB3*01:01 | 710-724 | NSIAIPTNFTISVTT | 1771 |
|  | IPTNFTISV | HLA-DRB3*01:01 | 711-725 | SIAIPTNFTISVTTE | 1788 |
|  | IPTNFTISV | HLA-DRB1*08:02 | 713-727 | AIPTNFTISVTTEIL | 1818 |
|  | IPTNFTISV | HLA-DPA1*01/DPB1*04:01 1 | 712-726 | IAIPTNFTISVTTEI | 1951 |
|  | IPTNFTISV | HLA-DRB1*09:01 | 710-724 | NSIAIPTNFTISVTT | 2531 |
|  | IPTNFTISV | HLA-DRB1*09:01 | 709-723 | NNSIAIPTNFTISVT | 2668 |
|  | IPTNFTISV | HLA-DRB1*09:01 | 711-725 | SIAIPTNFTISVTTE | 2762 |
|  | IPTNFTISV | HLA-DRB4*01:01 | 712-726 | IAIPTNFTISVTTEI | 2851 |
|  | LAGTITSGW | HLA-DRB1*07:01 | 874-888 | TSALLAGTITSGWTF | 384 |
|  | LAGTITSGW | HLA-DQA1*01:02/DQB1*06:02 | 876-890 | ALLAGTITSGWTFGA | 385 |
|  | LAGTITSGW | HLA-DRB1*04:01 | 876-890 | ALLAGTITSGWTFGA | 616 |
|  | LAGTITSGW | HLA-DRB1*04:05 | 874-888 | TSALLAGTITSGWTF | 1135 |
|  | LAGTITSGW | HLA-DRB1*04:05 | 875-889 | SALLAGTITSGWTFG | 1201 |
|  | LAGTITSGW | HLA-DRB1*07:01 | 873-887 | YTSALLAGTITSGWT | 1206 |
|  | LAGTITSGW | HLA-DRB1*04:05 | 876-890 | ALLAGTITSGWTFGA | 1409 |
|  | LAGTITSGW | HLA-DRB1*04:04 | 875-889 | SALLAGTITSGWTFG | 1908 |
|  | LAGTITSGW | HLA-DRB1*04:04 | 876-890 | ALLAGTITSGWTFGA | 1919 |
|  | LAGTITSGW | HLA-DRB1*11:01 | 874-888 | TSALLAGTITSGWTF | 2769 |
|  | LGAENSVAY | HLA-DQA1*05:01/DQB1*03:01 | 697-711 | MSLGAENSVAYSNNS | 120 |
|  | LGAENSVAY | HLA-DRB1*04:01 | 694-708 | AYTMSLGAENSVAYS | 362 |
|  | LGAENSVAY | HLA-DRB1*04:01 | 695-709 | YTMSLGAENSVAYSN | 401 |
|  | LGAENSVAY | HLA-DRB1*04:01 | 696-710 | TMSLGAENSVAYSNN | 403 |
|  | LGAENSVAY | HLA-DRB1*04:01 | 697-711 | MSLGAENSVAYSNNS | 436 |
|  | LGAENSVAY | HLA-DRB1*15:01 | 698-712 | SLGAENSVAYSNNSI | 629 |
|  | LGAENSVAY | HLA-DRB1*01:01 | 697-711 | MSLGAENSVAYSNNS | 747 |
|  | LGAENSVAY | HLA-DRB1*04:01 | 698-712 | SLGAENSVAYSNNSI | 834 |
|  | LGAENSVAY | HLA-DRB1*01:01 | 698-712 | SLGAENSVAYSNNSI | 915 |
|  | LGAENSVAY | HLA-DRB1*09:01 | 697-711 | MSLGAENSVAYSNNS | 943 |
|  | LGAENSVAY | HLA-DRB1*09:01 | 696-710 | TMSLGAENSVAYSNN | 959 |
|  | LGAENSVAY | HLA-DRB1*09:01 | 699-713 | LGAENSVAYSNNSIA | 1330 |
|  | LGAENSVAY | HLA-DRB1*04:04 | 697-711 | MSLGAENSVAYSNNS | 2152 |
|  | LGAENSVAY | HLA-DRB1*03:01 | 696-710 | TMSLGAENSVAYSNN | 2475 |
|  | LGAENSVAY | HLA-DRB1*03:01 | 695-709 | YTMSLGAENSVAYSN | 2476 |
|  | LGAENSVAY | HLA-DRB1*03:01 | 694-708 | AYTMSLGAENSVAYS | 2485 |
|  | LGAENSVAY | HLA-DRB1*03:01 | 697-711 | MSLGAENSVAYSNNS | 2487 |
|  | LGAENSVAY | HLA-DRB1*15:01 | 694-708 | AYTMSLGAENSVAYS | 2698 |
|  | LGAENSVAY | HLA-DRB3*01:01 | 694-708 | AYTMSLGAENSVAYS | 2710 |
|  | LGAENSVAY | HLA-DRB3*01:01 | 695-709 | YTMSLGAENSVAYSN | 2714 |
|  | LGAENSVAY | HLA-DRB3*01:01 | 696-710 | TMSLGAENSVAYSNN | 2776 |
|  | LGAENSVAY | HLA-DRB1*09:01 | 698-712 | SLGAENSVAYSNNSI | 2790 |
|  | LGAENSVAY | HLA-DRB3*01:01 | 697-711 | MSLGAENSVAYSNNS | 2817 |
|  | LGAENSVAY | HLA-DRB1*15:01 | 697-711 | MSLGAENSVAYSNNS | 2842 |
|  | LTDEMIAQY | HLA-DRB4*01:01 | 860-874 | VLPPLLTDEMIAQYT | 512 |
|  | LTDEMIAQY | HLA-DRB4*01:01 | 861-875 | LPPLLTDEMIAQYTS | 585 |
|  | LTDEMIAQY | HLA-DQA1*01:02/DQB1*06:02 | 865-879 | LTDEMIAQYTSALLA | 648 |
|  | LTDEMIAQY | HLA-DQA1*01:02/DQB1*06:02 | 863-877 | PLLTDEMIAQYTSAL | 722 |
|  | LTDEMIAQY | HLA-DQA1*01:02/DQB1*06:02 | 862-876 | PPLLTDEMIAQYTSA | 747 |
|  | LTDEMIAQY | HLA-DQA1*01:02/DQB1*06:02 | 861-875 | LPPLLTDEMIAQYTS | 786 |
|  | LTDEMIAQY | HLA-DQA1*01:02/DQB1*06:02 | 860-874 | VLPPLLTDEMIAQYT | 885 |
|  | LTDEMIAQY | HLA-DPA1*03:01/DPB1*04:02 | 863-877 | PLLTDEMIAQYTSAL | 913 |
|  | LTDEMIAQY | HLA-DQA1*01:02/DQB1*06:02 | 864-878 | LLTDEMIAQYTSALL | 943 |
|  | LTDEMIAQY | HLA-DPA1*02:01/DPB1*01:01 | 863-877 | PLLTDEMIAQYTSAL | 1495 |
|  | LTDEMIAQY | HLA-DQA1*05:01/DQB1*02:01 | 860-874 | VLPPLLTDEMIAQYT | 1534 |
|  | LTDEMIAQY | HLA-DRB1*12:01 | 863-877 | PLLTDEMIAQYTSAL | 1730 |
|  | LTDEMIAQY | HLA-DQA1*05:01/DQB1*02:01 | 863-877 | PLLTDEMIAQYTSAL | 1810 |
|  | LTDEMIAQY | HLA-DQA1*04:01/DQB1*04:02 | 863-877 | PLLTDEMIAQYTSAL | 2092 |
|  | LTDEMIAQY | HLA-DQA1*04:01/DQB1*04:02 | 862-876 | PPLLTDEMIAQYTSA | 2147 |
|  | LTDEMIAQY | HLA-DQA1*04:01/DQB1*04:02 | 861-875 | LPPLLTDEMIAQYTS | 2238 |
|  | LTDEMIAQY | HLA-DRB1*08:02 | 862-876 | PPLLTDEMIAQYTSA | 2312 |
|  | LTDEMIAQY | HLA-DQA1*03:01/DQB1*03:02 | 862-876 | PPLLTDEMIAQYTSA | 2354 |
|  | LTDEMIAQY | HLA-DQA1*03:01/DQB1*03:02 | 863-877 | PLLTDEMIAQYTSAL | 2429 |
|  | LTDEMIAQY | HLA-DRB1*08:02 | 861-875 | LPPLLTDEMIAQYTS | 2446 |
|  | LTDEMIAQY | HLA-DQA1*04:01/DQB1*04:02 | 860-874 | VLPPLLTDEMIAQYT | 2451 |
|  | LTDEMIAQY | HLA-DQA1*05:01/DQB1*02:01 | 862-876 | PPLLTDEMIAQYTSA | 2620 |
|  | NGVEGFNCY | HLA-DPA1*01:03/DPB1*02:01 | 478-492 | TPCNGVEGFNCYFPL | 2556 |
|  | NSIAIPTNF | HLA-DRB1*07:01 | 706-720 | AYSNNSIAIPTNFTI | 450 |
|  | NSIAIPTNF | HLA-DRB1*07:01 | 705-719 | VAYSNNSIAIPTNFT | 1039 |
|  | NSIAIPTNF | HLA-DRB1*09:01 | 705-719 | VAYSNNSIAIPTNFT | 1902 |
|  | NSIAIPTNF | HLA-DRB1*09:01 | 708-722 | SNNSIAIPTNFTISV | 2025 |
|  | NSIAIPTNF | HLA-DRB1*09:01 | 706-720 | AYSNNSIAIPTNFTI | 2592 |
|  | NSIAIPTNF | HLA-DRB1*09:01 | 707-721 | YSNNSIAIPTNFTIS | 2893 |
|  | PFFSNVTWF | HLA-DRB3*01:01 | 52-66 | QDLFLPFFSNVTWFH | 1452 |
|  | PFFSNVTWF | HLA-DQA1*01:02/DQB1*06:02 | 53-67 | DLFLPFFSNVTWFHA | 2245 |
|  | PYRVVVLSF | HLA-DRB1*04:05 | 502-516 | GVGYQPYRVVVLSFE | 149 |
|  | PYRVVVLSF | HLA-DRB1*08:02 | 502-516 | GVGYQPYRVVVLSFE | 1589 |
|  | PYRVVVLSF | HLA-DQA1*05:01/DQB1*02:01 | 502-516 | GVGYQPYRVVVLSFE | 1596 |
|  | PYRVVVLSF | HLA-DPA1*02:01/DPB1*05:01 | 502-516 | GVGYQPYRVVVLSFE | 1880 |
|  | PYRVVVLSF | HLA-DRB4*01:01 | 503-517 | VGYQPYRVVVLSFEL | 1881 |
|  | PYRVVVLSF | HLA-DRB4*01:01 | 502-516 | GVGYQPYRVVVLSFE | 2093 |
|  | PYRVVVLSF | HLA-DQA1*03:01/DQB1*03:02 | 502-516 | GVGYQPYRVVVLSFE | 2135 |
|  | QLTPTWRVY | HLA-DRB1*13:02 | 628-642 | QLTPTWRVYSTGSNV | 469 |
|  | QSAPHGVVF | HLA-DRB1*07:01 | 1054-1068 | QSAPHGVVFLHVTYV | 244 |
|  | QSAPHGVVF | HLA-DRB1*09:01 | 1054-1068 | QSAPHGVVFLHVTYV | 710 |
|  | QSAPHGVVF | HLA-DRB1*13:02 | 1054-1068 | QSAPHGVVFLHVTYV | 822 |
|  | QYIKWPWYI | HLA-DRB1*15:01 | 1204-1218 | GKYEQYIKWPWYIWL | 405 |
|  | QYIKWPWYI | HLA-DRB1*15:01 | 1203-1217 | LGKYEQYIKWPWYIW | 413 |
|  | QYIKWPWYI | HLA-DRB1*15:01 | 1208-1222 | QYIKWPWYIWLGFIA | 457 |
|  | QYIKWPWYI | HLA-DRB1*15:01 | 1205-1219 | KYEQYIKWPWYIWLG | 525 |
|  | QYIKWPWYI | HLA-DRB1*15:01 | 1206-1220 | YEQYIKWPWYIWLGF | 643 |
|  | QYIKWPWYI | HLA-DRB1*15:01 | 1207-1221 | EQYIKWPWYIWLGFI | 1144 |
|  | QYIKWPWYI | HLA-DRB3*01:01 | 1206-1220 | YEQYIKWPWYIWLGF | 2162 |
|  | QYIKWPWYI | HLA-DRB3*01:01 | 1204-1218 | GKYEQYIKWPWYIWL | 2423 |
|  | QYIKWPWYI | HLA-DRB3*01:01 | 1205-1219 | KYEQYIKWPWYIWLG | 2868 |
|  | TLKSFTVEK | HLA-DPA1*02:01/DPB1*01:01 | 297-311 | SETKCTLKSFTVEKG | 922 |
|  | TLLALHRSY | HLA-DQA1*05:01/DQB1*03:01 | 235-249 | ITRFQTLLALHRSYL | 2540 |
|  | VTYVPAQEK | HLA-DRB1*04:04 | 1060-1074 | VVFLHVTYVPAQEKN | 169 |
|  | VTYVPAQEK | HLA-DRB5*01:01 | 1062-1076 | FLHVTYVPAQEKNFT | 205 |
|  | VTYVPAQEK | HLA-DRB5*01:01 | 1061-1075 | VFLHVTYVPAQEKNF | 206 |
|  | VTYVPAQEK | HLA-DRB5*01:01 | 1063-1077 | LHVTYVPAQEKNFTT | 211 |
|  | VTYVPAQEK | HLA-DRB5*01:01 | 1060-1074 | VVFLHVTYVPAQEKN | 230 |
|  | VTYVPAQEK | HLA-DRB1*04:04 | 1061-1075 | VFLHVTYVPAQEKNF | 268 |
|  | VTYVPAQEK | HLA-DRB1*04:04 | 1062-1076 | FLHVTYVPAQEKNFT | 285 |
|  | VTYVPAQEK | HLA-DRB1*01:01 | 1063-1077 | LHVTYVPAQEKNFTT | 345 |
|  | VTYVPAQEK | HLA-DRB1*04:04 | 1063-1077 | LHVTYVPAQEKNFTT | 398 |
|  | VTYVPAQEK | HLA-DRB5*01:01 | 1064-1078 | HVTYVPAQEKNFTTA | 512 |
|  | VTYVPAQEK | HLA-DRB5*01:01 | 1065-1079 | VTYVPAQEKNFTTAP | 512 |
|  | VTYVPAQEK | HLA-DRB4*01:01 | 1060-1074 | VVFLHVTYVPAQEKN | 610 |
|  | VTYVPAQEK | HLA-DRB4*01:01 | 1062-1076 | FLHVTYVPAQEKNFT | 669 |
|  | VTYVPAQEK | HLA-DRB4*01:01 | 1063-1077 | LHVTYVPAQEKNFTT | 675 |
|  | VTYVPAQEK | HLA-DRB1*01:01 | 1065-1079 | VTYVPAQEKNFTTAP | 711 |
|  | VTYVPAQEK | HLA-DRB1*01:01 | 1064-1078 | HVTYVPAQEKNFTTA | 715 |
|  | VTYVPAQEK | HLA-DRB4*01:01 | 1061-1075 | VFLHVTYVPAQEKNF | 716 |
|  | VTYVPAQEK | HLA-DRB1*07:01 | 1061-1075 | VFLHVTYVPAQEKNF | 805 |
|  | VTYVPAQEK | HLA-DRB1*07:01 | 1062-1076 | FLHVTYVPAQEKNFT | 813 |
|  | VTYVPAQEK | HLA-DRB1*07:01 | 1063-1077 | LHVTYVPAQEKNFTT | 980 |
|  | VTYVPAQEK | HLA-DRB1*04:04 | 1064-1078 | HVTYVPAQEKNFTTA | 1029 |
|  | VTYVPAQEK | HLA-DRB1*04:04 | 1065-1079 | VTYVPAQEKNFTTAP | 1106 |
|  | VTYVPAQEK | HLA-DQA1*04:01/DQB1*04:02 | 1061-1075 | VFLHVTYVPAQEKNF | 1166 |
|  | VTYVPAQEK | HLA-DQA1*04:01/DQB1*04:02 | 1060-1074 | VVFLHVTYVPAQEKN | 1185 |
|  | VTYVPAQEK | HLA-DQA1*04:01/DQB1*04:02 | 1062-1076 | FLHVTYVPAQEKNFT | 1197 |
|  | VTYVPAQEK | HLA-DQA1*03:01/DQB1*03:02 | 1060-1074 | VVFLHVTYVPAQEKN | 1284 |
|  | VTYVPAQEK | HLA-DQA1*03:01/DQB1*03:02 | 1061-1075 | VFLHVTYVPAQEKNF | 1364 |
|  | VTYVPAQEK | HLA-DQA1*03:01/DQB1*03:02 | 1062-1076 | FLHVTYVPAQEKNFT | 1397 |
|  | VTYVPAQEK | HLA-DQA1*04:01/DQB1*04:02 | 1063-1077 | LHVTYVPAQEKNFTT | 1485 |
|  | VTYVPAQEK | HLA-DRB4*01:01 | 1064-1078 | HVTYVPAQEKNFTTA | 1692 |
|  | VTYVPAQEK | HLA-DRB4*01:01 | 1065-1079 | VTYVPAQEKNFTTAP | 1697 |
|  | VTYVPAQEK | HLA-DQA1*05:01/DQB1*03:01 | 1063-1077 | LHVTYVPAQEKNFTT | 1822 |
|  | VTYVPAQEK | HLA-DRB1*11:01 | 1063-1077 | LHVTYVPAQEKNFTT | 1862 |
|  | VTYVPAQEK | HLA-DQA1*03:01/DQB1*03:02 | 1063-1077 | LHVTYVPAQEKNFTT | 2044 |
|  | VTYVPAQEK | HLA-DQA1*01:02/DQB1*06:02 | 1062-1076 | FLHVTYVPAQEKNFT | 2093 |
|  | VTYVPAQEK | HLA-DRB1*04:05 | 1063-1077 | LHVTYVPAQEKNFTT | 2400 |
|  | VTYVPAQEK | HLA-DRB1*15:01 | 1062-1076 | FLHVTYVPAQEKNFT | 2506 |
|  | VTYVPAQEK | HLA-DRB1*15:01 | 1061-1075 | VFLHVTYVPAQEKNF | 2553 |
|  | VTYVPAQEK | HLA-DRB1*15:01 | 1063-1077 | LHVTYVPAQEKNFTT | 2559 |
|  | VYAWNRKRI | HLA-DRB1*15:01 | 348-362 | ASVYAWNRKRISNCV | 1677 |
|  | VYAWNRKRI | HLA-DPA1*02:01/DPB1*01:01 | 345-359 | TRFASVYAWNRKRIS | 1710 |
|  | VYAWNRKRI | HLA-DPA1*02:01/DPB1*01:01 | 346-360 | RFASVYAWNRKRISN | 1965 |
|  | VYAWNRKRI | HLA-DRB1*13:02 | 348-362 | ASVYAWNRKRISNCV | 2304 |
|  | VYAWNRKRI | HLA-DPA1*02:01/DPB1*01:01 | 347-361 | FASVYAWNRKRISNC | 2608 |
|  | WMESEFRVY | HLA-DPA1*01:03/DPB1*02:01 | 149-163 | NKSWMESEFRVYSSA | 511 |
|  | WMESEFRVY | HLA-DPA1*01:03/DPB1*02:01 | 148-162 | NNKSWMESEFRVYSS | 516 |
|  | WMESEFRVY | HLA-DPA1*01:03/DPB1*02:01 | 147-161 | KNNKSWMESEFRVYS | 517 |
|  | WMESEFRVY | HLA-DPA1*01:03/DPB1*02:01 | 150-164 | KSWMESEFRVYSSAN | 544 |
|  | WMESEFRVY | HLA-DPA1*01/DPB1*04:01 1 | 149-163 | NKSWMESEFRVYSSA | 813 |
|  | WMESEFRVY | HLA-DPA1*01/DPB1*04:01 1 | 147-161 | KNNKSWMESEFRVYS | 818 |
|  | WMESEFRVY | HLA-DPA1*01/DPB1*04:01 1 | 148-162 | NNKSWMESEFRVYSS | 819 |
|  | WMESEFRVY | HLA-DPA1*01/DPB1*04:01 | 150-164 | KSWMESEFRVYSSAN | 874 |
|  | WMESEFRVY | HLA-DPA1*02:01/DPB1*01:01 | 148-162 | NNKSWMESEFRVYSS | 1000 |
|  | WMESEFRVY | HLA-DPA1*02:01/DPB1*01:01 | 147-161 | KNNKSWMESEFRVYS | 1006 |
|  | WMESEFRVY | HLA-DPA1*02:01/DPB1*01:01 | 149-163 | NKSWMESEFRVYSSA | 1007 |
|  | WMESEFRVY | HLA-DPA1*02:01/DPB1*01:01 | 150-164 | KSWMESEFRVYSSAN | 1087 |
|  | WMESEFRVY | HLA-DPA1*01:03/DPB1*02:01 | 151-165 | SWMESEFRVYSSANN | 1475 |
|  | WMESEFRVY | HLA-DPA1*01:03/DPB1*02:01 | 152-166 | WMESEFRVYSSANNC | 1643 |
|  | WMESEFRVY | HLA-DPA1*02:01/DPB1*05:01 | 148-162 | NNKSWMESEFRVYSS | 1714 |
|  | WMESEFRVY | HLA-DPA1*02:01/DPB1*05:01 | 147-161 | KNNKSWMESEFRVYS | 1720 |
|  | WMESEFRVY | HLA-DPA1*02:01/DPB1*05:01 | 149-163 | NKSWMESEFRVYSSA | 1735 |
|  | WMESEFRVY | HLA-DRB1*11:01 | 147-161 | KNNKSWMESEFRVYS | 1816 |
|  | WMESEFRVY | HLA-DPA1*02:01/DPB1*05:01 | 150-164 | KSWMESEFRVYSSAN | 2090 |
|  | WMESEFRVY | HLA-DPA1*03:01/DPB1*04:02 | 149-163 | NKSWMESEFRVYSSA | 2292 |
|  | WMESEFRVY | HLA-DPA1*01/DPB1*04:01 | 151-165 | SWMESEFRVYSSANN | 2311 |
|  | WMESEFRVY | HLA-DPA1*03:01/DPB1*04:02 | 150-164 | KSWMESEFRVYSSAN | 2329 |
|  | WMESEFRVY | HLA-DPA1*03:01/DPB1*04:02 | 148-162 | NNKSWMESEFRVYSS | 2352 |
|  | WMESEFRVY | HLA-DQA1*01:01/DQB1*05:01 | 147-161 | KNNKSWMESEFRVYS | 2364 |
|  | WMESEFRVY | HLA-DPA1*02:01/DPB1*01:01 | 151-165 | SWMESEFRVYSSANN | 2364 |
|  | WMESEFRVY | HLA-DPA1*03:01/DPB1*04:02 | 147-161 | KNNKSWMESEFRVYS | 2377 |
|  | WMESEFRVY | HLA-DQA1*01:01/DQB1*05:01 | 148-162 | NNKSWMESEFRVYSS | 2393 |
|  | WMESEFRVY | HLA-DQA1*05:01/DQB1*02:01 | 147-161 | KNNKSWMESEFRVYS | 2482 |
|  | WMESEFRVY | HLA-DQA1*01:01/DQB1*05:01 | 149-163 | NKSWMESEFRVYSSA | 2652 |
|  | WMESEFRVY | HLA-DPA1*01/DPB1*04:01 1 | 152-166 | WMESEFRVYSSANNC | 2782 |
|  | WMESEFRVY | HLA-DPA1*02:01/DPB1*01:01 | 152-166 | WMESEFRVYSSANNC | 2834 |
|  | WMESEFRVY | HLA-DQA1*05:01/DQB1*02:01 | 148-162 | NNKSWMESEFRVYSS | 2853 |
|  | WMESEFRVY | HLA-DRB1*03:01 | 150-164 | KSWMESEFRVYSSAN | 2898 |
|  | WMESEFRVY | HLA-DRB1*03:01 | 147-161 | KNNKSWMESEFRVYS | 2906 |
|  | WPWYIWLGF | HLA-DRB1*04:04 | 1210-1224 | IKWPWYIWLGFIAGL | 1322 |
|  | WPWYIWLGF | HLA-DQA1*05:01/DQB1*02:01 | 1210-1224 | IKWPWYIWLGFIAGL | 1341 |
|  | WPWYIWLGF | HLA-DRB1*04:04 | 1209-1223 | YIKWPWYIWLGFIAG | 1427 |
|  | WPWYIWLGF | HLA-DQA1*05:01/DQB1*02:01 | 1209-1223 | YIKWPWYIWLGFIAG | 1778 |
|  | WPWYIWLGF | HLA-DQA1*05:01/DQB1*02:01 | 1208-1222 | QYIKWPWYIWLGFIA | 1906 |
|  | WPWYIWLGF | HLA-DRB1*04:05 | 1209-1223 | YIKWPWYIWLGFIAG | 2487 |
|  | WPWYIWLGF | HLA-DQA1*05:01/DQB1*02:01 | 1207-1221 | EQYIKWPWYIWLGFI | 2599 |
|  | WPWYIWLGF | HLA-DRB3*01:01 | 1207-1221 | EQYIKWPWYIWLGFI | 2710 |
|  | WPWYIWLGF | HLA-DRB3*01:01 | 1208-1222 | QYIKWPWYIWLGFIA | 2881 |
|  | WPWYIWLGF | HLA-DRB1*04:04 | 1207-1221 | EQYIKWPWYIWLGFI | 2929 |
|  | WPWYIWLGF | HLA-DRB4*01:01 | 1209-1223 | YIKWPWYIWLGFIAG | 2954 |
|  | WTAGAAAYY | HLA-DRB1*09:01 | 253-267 | DSSSGWTAGAAAYYV | 146 |
|  | WTAGAAAYY | HLA-DRB1*09:01 | 255-269 | SSGWTAGAAAYYVGY | 147 |
|  | WTAGAAAYY | HLA-DRB1*09:01 | 254-268 | SSSGWTAGAAAYYVG | 148 |
|  | WTAGAAAYY | HLA-DRB1*09:01 | 256-270 | SGWTAGAAAYYVGYL | 149 |
|  | WTAGAAAYY | HLA-DRB1*01:01 | 254-268 | SSSGWTAGAAAYYVG | 188 |
|  | WTAGAAAYY | HLA-DRB1*01:01 | 255-269 | SSGWTAGAAAYYVGY | 192 |
|  | WTAGAAAYY | HLA-DRB1*01:01 | 253-267 | DSSSGWTAGAAAYYV | 193 |
|  | WTAGAAAYY | HLA-DRB1*12:01 | 253-267 | DSSSGWTAGAAAYYV | 205 |
|  | WTAGAAAYY | HLA-DRB1*12:01 | 254-268 | SSSGWTAGAAAYYVG | 206 |
|  | WTAGAAAYY | HLA-DRB1*12:01 | 255-269 | SSGWTAGAAAYYVGY | 206 |
|  | WTAGAAAYY | HLA-DRB1*12:01 | 256-270 | SGWTAGAAAYYVGYL | 219 |
|  | WTAGAAAYY | HLA-DQA1*01:02/DQB1*06:02 | 253-267 | DSSSGWTAGAAAYYV | 358 |
|  | WTAGAAAYY | HLA-DRB1*09:01 | 257-271 | GWTAGAAAYYVGYLQ | 419 |
|  | WTAGAAAYY | HLA-DRB1*09:01 | 258-272 | WTAGAAAYYVGYLQP | 437 |
|  | WTAGAAAYY | HLA-DRB1*01:01 | 258-272 | WTAGAAAYYVGYLQP | 545 |
|  | WTAGAAAYY | HLA-DRB1*12:01 | 257-271 | GWTAGAAAYYVGYLQ | 576 |
|  | WTAGAAAYY | HLA-DRB1*12:01 | 258-272 | WTAGAAAYYVGYLQP | 673 |
|  | WTAGAAAYY | HLA-DRB1*07:01 | 253-267 | DSSSGWTAGAAAYYV | 822 |
|  | WTAGAAAYY | HLA-DRB5*01:01 | 253-267 | DSSSGWTAGAAAYYV | 1001 |
|  | WTAGAAAYY | HLA-DQA1*01:02/DQB1*06:02 | 258-272 | WTAGAAAYYVGYLQP | 1042 |
|  | WTAGAAAYY | HLA-DRB1*15:01 | 253-267 | DSSSGWTAGAAAYYV | 1455 |
|  | WTAGAAAYY | HLA-DRB3*01:01 | 253-267 | DSSSGWTAGAAAYYV | 1700 |
|  | WTAGAAAYY | HLA-DRB3*01:01 | 255-269 | SSGWTAGAAAYYVGY | 1716 |
|  | WTAGAAAYY | HLA-DRB3*01:01 | 256-270 | SGWTAGAAAYYVGYL | 1739 |
|  | WTAGAAAYY | HLA-DRB3*01:01 | 254-268 | SSSGWTAGAAAYYVG | 1740 |
|  | WTAGAAAYY | HLA-DQA1*05:01/DQB1*02:01 | 256-270 | SGWTAGAAAYYVGYL | 1809 |
|  | WTAGAAAYY | HLA-DQA1*05:01/DQB1*02:01 | 255-269 | SSGWTAGAAAYYVGY | 1891 |
|  | WTAGAAAYY | HLA-DQA1*05:01/DQB1*02:01 | 253-267 | DSSSGWTAGAAAYYV | 1912 |
|  | WTAGAAAYY | HLA-DQA1*05:01/DQB1*02:01 | 254-268 | SSSGWTAGAAAYYVG | 1975 |
|  | WTAGAAAYY | HLA-DRB1*13:02 | 253-267 | DSSSGWTAGAAAYYV | 2757 |
|  | WTAGAAAYY | HLA-DRB1*13:02 | 255-269 | SSGWTAGAAAYYVGY | 2803 |
|  | WTAGAAAYY | HLA-DRB1*13:02 | 254-268 | SSSGWTAGAAAYYVG | 2821 |
|  | WTAGAAAYY | HLA-DQA1*05:01/DQB1*02:01 | 258-272 | WTAGAAAYYVGYLQP | 2857 |
|  | WTAGAAAYY | HLA-DRB1*13:02 | 256-270 | SGWTAGAAAYYVGYL | 2959 |
|  | YLQPRTFLL | HLA-DRB1*01:01 | 264-278 | AYYVGYLQPRTFLLK | 26 |
|  | YLQPRTFLL | HLA-DRB1*01:01 | 265-279 | YYVGYLQPRTFLLKY | 28 |
|  | YLQPRTFLL | HLA-DRB1*01:01 | 266-280 | YVGYLQPRTFLLKYN | 31 |
|  | YLQPRTFLL | HLA-DRB1*01:01 | 267-281 | VGYLQPRTFLLKYNE | 32 |
|  | YLQPRTFLL | HLA-DPA1*02:01/DPB1*01:01 | 264-278 | AYYVGYLQPRTFLLK | 70 |
|  | YLQPRTFLL | HLA-DRB1*01:01 | 268-282 | GYLQPRTFLLKYNEN | 91 |
|  | YLQPRTFLL | HLA-DRB1*01:01 | 269-283 | YLQPRTFLLKYNENG | 93 |
|  | YLQPRTFLL | HLA-DPA1*01:03/DPB1*02:01 | 264-278 | AYYVGYLQPRTFLLK | 97 |
|  | YLQPRTFLL | HLA-DRB1*07:01 | 265-279 | YYVGYLQPRTFLLKY | 131 |
|  | YLQPRTFLL | HLA-DRB1*07:01 | 264-278 | AYYVGYLQPRTFLLK | 133 |
|  | YLQPRTFLL | HLA-DPA1*01/DPB1*04:01 1 | 264-278 | AYYVGYLQPRTFLLK | 133 |
|  | YLQPRTFLL | HLA-DRB1*07:01 | 266-280 | YVGYLQPRTFLLKYN | 160 |
|  | YLQPRTFLL | HLA-DRB1*07:01 | 267-281 | VGYLQPRTFLLKYNE | 162 |
|  | YLQPRTFLL | HLA-DPA1*03:01/DPB1*04:02 | 264-278 | AYYVGYLQPRTFLLK | 179 |
|  | YLQPRTFLL | HLA-DRB1*04:01 | 264-278 | AYYVGYLQPRTFLLK | 263 |
|  | YLQPRTFLL | HLA-DRB1*04:01 | 265-279 | YYVGYLQPRTFLLKY | 362 |
|  | YLQPRTFLL | HLA-DRB1*04:01 | 266-280 | YVGYLQPRTFLLKYN | 395 |
|  | YLQPRTFLL | HLA-DRB5*01:01 | 266-280 | YVGYLQPRTFLLKYN | 397 |
|  | YLQPRTFLL | HLA-DRB5*01:01 | 267-281 | VGYLQPRTFLLKYNE | 403 |
|  | YLQPRTFLL | HLA-DPA1*02:01/DPB1*05:01 | 266-280 | YVGYLQPRTFLLKYN | 476 |
|  | YLQPRTFLL | HLA-DPA1*02:01/DPB1*05:01 | 267-281 | VGYLQPRTFLLKYNE | 483 |
|  | YLQPRTFLL | HLA-DPA1*02:01/DPB1*05:01 | 265-279 | YYVGYLQPRTFLLKY | 506 |
|  | YLQPRTFLL | HLA-DPA1*02:01/DPB1*05:01 | 264-278 | AYYVGYLQPRTFLLK | 512 |
|  | YLQPRTFLL | HLA-DRB1*07:01 | 269-283 | YLQPRTFLLKYNENG | 513 |
|  | YLQPRTFLL | HLA-DRB1*07:01 | 268-282 | GYLQPRTFLLKYNEN | 516 |
|  | YLQPRTFLL | HLA-DRB1*04:01 | 269-283 | YLQPRTFLLKYNENG | 557 |
|  | YLQPRTFLL | HLA-DRB1*04:01 | 267-281 | VGYLQPRTFLLKYNE | 560 |
|  | YLQPRTFLL | HLA-DRB1*15:01 | 267-281 | VGYLQPRTFLLKYNE | 958 |
|  | YLQPRTFLL | HLA-DPA1*02:01/DPB1*05:01 | 269-283 | YLQPRTFLLKYNENG | 966 |
|  | YLQPRTFLL | HLA-DRB5*01:01 | 269-283 | YLQPRTFLLKYNENG | 1002 |
|  | YLQPRTFLL | HLA-DRB1*04:05 | 269-283 | YLQPRTFLLKYNENG | 1022 |
|  | YLQPRTFLL | HLA-DRB1*12:01 | 264-278 | AYYVGYLQPRTFLLK | 1304 |
|  | YLQPRTFLL | HLA-DRB1*04:01 | 268-282 | GYLQPRTFLLKYNEN | 1488 |
|  | YLQPRTFLL | HLA-DRB5*01:01 | 268-282 | GYLQPRTFLLKYNEN | 1530 |
|  | YLQPRTFLL | HLA-DRB1*04:05 | 267-281 | VGYLQPRTFLLKYNE | 1702 |
|  | YLQPRTFLL | HLA-DRB1*03:01 | 265-279 | YYVGYLQPRTFLLKY | 2362 |
|  | YLQPRTFLL | HLA-DRB1*03:01 | 264-278 | AYYVGYLQPRTFLLK | 2369 |
|  | YLQPRTFLL | HLA-DRB1*03:01 | 266-280 | YVGYLQPRTFLLKYN | 2494 |
|  | YLQPRTFLL | HLA-DRB1*03:01 | 267-281 | VGYLQPRTFLLKYNE | 2521 |
|  | YQPYRVVVL | HLA-DRB1*03:01 | 505-519 | YQPYRVVVLSFELLH | 106 |
|  | YQPYRVVVL | HLA-DRB1*01:01 | 503-517 | VGYQPYRVVVLSFEL | 152 |
|  | YQPYRVVVL | HLA-DRB1*01:01 | 500-514 | TNGVGYQPYRVVVLS | 176 |
|  | YQPYRVVVL | HLA-DRB1*01:01 | 502-516 | GVGYQPYRVVVLSFE | 178 |
|  | YQPYRVVVL | HLA-DRB1*01:01 | 501-515 | NGVGYQPYRVVVLSF | 182 |
|  | YQPYRVVVL | HLA-DRB1*07:01 | 503-517 | VGYQPYRVVVLSFEL | 206 |
|  | YQPYRVVVL | HLA-DRB1*15:01 | 503-517 | VGYQPYRVVVLSFEL | 238 |
|  | YQPYRVVVL | HLA-DRB1*15:01 | 501-515 | NGVGYQPYRVVVLSF | 322 |
|  | YQPYRVVVL | HLA-DRB1*15:01 | 502-516 | GVGYQPYRVVVLSFE | 370 |
|  | YQPYRVVVL | HLA-DRB1*07:01 | 501-515 | NGVGYQPYRVVVLSF | 422 |
|  | YQPYRVVVL | HLA-DRB1*07:01 | 502-516 | GVGYQPYRVVVLSFE | 430 |
|  | YQPYRVVVL | HLA-DRB1*07:01 | 500-514 | TNGVGYQPYRVVVLS | 451 |
|  | YQPYRVVVL | HLA-DPA1*02:01/DPB1*01:01 | 503-517 | VGYQPYRVVVLSFEL | 577 |
|  | YQPYRVVVL | HLA-DPA1*03:01/DPB1*04:02 | 503-517 | VGYQPYRVVVLSFEL | 683 |
|  | YQPYRVVVL | HLA-DPA1*03:01/DPB1*04:02 | 501-515 | NGVGYQPYRVVVLSF | 767 |
|  | YQPYRVVVL | HLA-DRB1*04:01 | 502-516 | GVGYQPYRVVVLSFE | 774 |
|  | YQPYRVVVL | HLA-DRB1*04:01 | 503-517 | VGYQPYRVVVLSFEL | 775 |
|  | YQPYRVVVL | HLA-DPA1*03:01/DPB1*04:02 | 500-514 | TNGVGYQPYRVVVLS | 776 |
|  | YQPYRVVVL | HLA-DPA1*03:01/DPB1*04:02 | 502-516 | GVGYQPYRVVVLSFE | 786 |
|  | YQPYRVVVL | HLA-DRB1*09:01 | 503-517 | VGYQPYRVVVLSFEL | 798 |
|  | YQPYRVVVL | HLA-DRB1*09:01 | 500-514 | TNGVGYQPYRVVVLS | 821 |
|  | YQPYRVVVL | HLA-DRB1*04:01 | 501-515 | NGVGYQPYRVVVLSF | 825 |
|  | YQPYRVVVL | HLA-DRB1*09:01 | 501-515 | NGVGYQPYRVVVLSF | 828 |
|  | YQPYRVVVL | HLA-DRB1*04:01 | 500-514 | TNGVGYQPYRVVVLS | 842 |
|  | YQPYRVVVL | HLA-DRB1*09:01 | 502-516 | GVGYQPYRVVVLSFE | 846 |
|  | YQPYRVVVL | HLA-DQA1*05:01/DQB1*03:01 | 502-516 | GVGYQPYRVVVLSFE | 999 |
|  | YQPYRVVVL | HLA-DPA1*01:03/DPB1*02:01 | 503-517 | VGYQPYRVVVLSFEL | 1033 |
|  | YQPYRVVVL | HLA-DPA1*02:01/DPB1*01:01 | 502-516 | GVGYQPYRVVVLSFE | 1036 |
|  | YQPYRVVVL | HLA-DQA1*05:01/DQB1*03:01 | 503-517 | VGYQPYRVVVLSFEL | 1144 |
|  | YQPYRVVVL | HLA-DPA1*02:01/DPB1*01:01 | 501-515 | NGVGYQPYRVVVLSF | 1274 |
|  | YQPYRVVVL | HLA-DPA1*01:03/DPB1*02:01 | 502-516 | GVGYQPYRVVVLSFE | 1311 |
|  | YQPYRVVVL | HLA-DPA1*02:01/DPB1*01:01 | 500-514 | TNGVGYQPYRVVVLS | 1334 |
|  | YQPYRVVVL | HLA-DRB1*04:01 | 504-518 | GYQPYRVVVLSFELL | 1345 |
|  | YQPYRVVVL | HLA-DRB3*01:01 | 502-516 | GVGYQPYRVVVLSFE | 1363 |
|  | YQPYRVVVL | HLA-DRB3*01:01 | 501-515 | NGVGYQPYRVVVLSF | 1373 |
|  | YQPYRVVVL | HLA-DRB3*01:01 | 500-514 | TNGVGYQPYRVVVLS | 1406 |
|  | YQPYRVVVL | HLA-DRB3*01:01 | 503-517 | VGYQPYRVVVLSFEL | 1450 |
|  | YQPYRVVVL | HLA-DRB1*04:05 | 501-515 | NGVGYQPYRVVVLSF | 1481 |
|  | YQPYRVVVL | HLA-DPA1*01/DPB1*04:01 1 | 500-514 | TNGVGYQPYRVVVLS | 1557 |
|  | YQPYRVVVL | HLA-DPA1*01/DPB1*04:01 1 | 501-515 | NGVGYQPYRVVVLSF | 1641 |
|  | YQPYRVVVL | HLA-DPA1*01/DPB1*04:01 1 | 503-517 | VGYQPYRVVVLSFEL | 1664 |
|  | YQPYRVVVL | HLA-DRB1*04:04 | 503-517 | VGYQPYRVVVLSFEL | 1804 |
|  | YQPYRVVVL | HLA-DPA1*01/DPB1*04:01 1 | 502-516 | GVGYQPYRVVVLSFE | 1822 |
|  | YQPYRVVVL | HLA-DRB1*04:04 | 502-516 | GVGYQPYRVVVLSFE | 1992 |
|  | YQPYRVVVL | HLA-DRB5*01:01 | 502-516 | GVGYQPYRVVVLSFE | 2042 |
|  | YQPYRVVVL | HLA-DQA1*05:01/DQB1*03:01 | 504-518 | GYQPYRVVVLSFELL | 2401 |
|  | YQPYRVVVL | HLA-DRB1*08:02 | 501-515 | NGVGYQPYRVVVLSF | 2408 |
|  | YQPYRVVVL | HLA-DRB1*04:05 | 500-514 | TNGVGYQPYRVVVLS | 2600 |
|  | YQPYRVVVL | HLA-DQA1*05:01/DQB1*03:01 | 505-519 | YQPYRVVVLSFELLH | 2714 |
|  | YQPYRVVVL | HLA-DQA1*01:02/DQB1*06:02 | 502-516 | GVGYQPYRVVVLSFE | 2891 |
|  | YQPYRVVVL | HLA-DRB1*08:02 | 500-514 | TNGVGYQPYRVVVLS | 2978 |
|  | YQPYRVVVL | HLA-DQA1*05:01/DQB1*02:01 | 501-515 | NGVGYQPYRVVVLSF | 2986 |
| **ORF3a protein** | | | | | |
| **No** | **Core sequence** | **MHC II alleles** | **position** | **Peptide** | **IC50 value** |
|  | EEHVQIHTI | HLA-DRB4*01:01 | 238-252 | DEPEEHVQIHTIDGS | 470 |
|  | EEHVQIHTI | HLA-DQA1*01:02/DQB1*06:02 | 238-252 | DEPEEHVQIHTIDGS | 763 |
|  | EEHVQIHTI | HLA-DQA1*01:02/DQB1*06:02 | 239-253 | EPEEHVQIHTIDGSS | 781 |
|  | EEHVQIHTI | HLA-DQA1*01:02/DQB1*06:02 | 237-251 | VDEPEEHVQIHTIDG | 1031 |
|  | EEHVQIHTI | HLA-DRB3*01:01 | 241-255 | EEHVQIHTIDGSSGV | 1086 |
|  | EEHVQIHTI | HLA-DQA1*01:02/DQB1*06:02 | 236-250 | IVDEPEEHVQIHTID | 1138 |
|  | EEHVQIHTI | HLA-DQA1*01:02/DQB1*06:02 | 241-255 | EEHVQIHTIDGSSGV | 1427 |
|  | EEHVQIHTI | HLA-DQA1*01:02/DQB1*06:02 | 240-254 | PEEHVQIHTIDGSSG | 1440 |
|  | EEHVQIHTI | HLA-DRB4*01:01 | 237-251 | VDEPEEHVQIHTIDG | 1615 |
|  | EEHVQIHTI | HLA-DRB4*01:01 | 236-250 | IVDEPEEHVQIHTID | 1877 |
|  | EEHVQIHTI | HLA-DRB1*07:01 | 239-253 | EPEEHVQIHTIDGSS | 2319 |
|  | EEHVQIHTI | HLA-DRB1*07:01 | 237-251 | VDEPEEHVQIHTIDG | 2466 |
|  | EEHVQIHTI | HLA-DRB1*07:01 | 238-252 | DEPEEHVQIHTIDGS | 2478 |
|  | EEHVQIHTI | HLA-DRB1*07:01 | 240-254 | PEEHVQIHTIDGSSG | 2520 |
|  | FLCWHTNCY | HLA-DQA1*01:01/DQB1*05:01 | 141-155 | YDANYFLCWHTNCYD | 1189 |
|  | FLCWHTNCY | HLA-DQA1*01:01/DQB1*05:01 | 143-157 | ANYFLCWHTNCYDYC | 1211 |
|  | FLCWHTNCY | HLA-DQA1*01:01/DQB1*05:01 | 146-160 | FLCWHTNCYDYCIPY | 1235 |
|  | FLCWHTNCY | HLA-DQA1*01:01/DQB1*05:01 | 144-158 | NYFLCWHTNCYDYCI | 1466 |
|  | FLCWHTNCY | HLA-DQA1*01:01/DQB1*05:01 | 142-156 | DANYFLCWHTNCYDY | 1811 |
|  | FLCWHTNCY | HLA-DQA1*01:01/DQB1*05:01 | 145-159 | YFLCWHTNCYDYCIP | 2358 |
|  | FLCWHTNCY | HLA-DRB1*12:01 | 143-157 | ANYFLCWHTNCYDYC | 2920 |
|  | FLCWHTNCY | HLA-DRB1*01:01 | 144-158 | NYFLCWHTNCYDYCI | 2942 |
|  | FLYLYALVY | HLA-DRB1*01:01 | 101-115 | LEAPFLYLYALVYFL | 7 |
|  | FLYLYALVY | HLA-DRB1*01:01 | 102-116 | EAPFLYLYALVYFLQ | 7 |
|  | FLYLYALVY | HLA-DRB1*01:01 | 103-117 | APFLYLYALVYFLQS | 7 |
|  | FLYLYALVY | HLA-DRB1*01:01 | 100-114 | GLEAPFLYLYALVYF | 9 |
|  | FLYLYALVY | HLA-DRB5*01:01 | 101-115 | LEAPFLYLYALVYFL | 13 |
|  | FLYLYALVY | HLA-DRB5*01:01 | 102-116 | EAPFLYLYALVYFLQ | 13 |
|  | FLYLYALVY | HLA-DRB5*01:01 | 103-117 | APFLYLYALVYFLQS | 13 |
|  | FLYLYALVY | HLA-DRB5*01:01 | 100-114 | GLEAPFLYLYALVYF | 15 |
|  | FLYLYALVY | HLA-DRB1*01:01 | 104-118 | PFLYLYALVYFLQSI | 15 |
|  | FLYLYALVY | HLA-DRB1*01:01 | 105-119 | FLYLYALVYFLQSIN | 15 |
|  | FLYLYALVY | HLA-DRB1*04:04 | 101-115 | LEAPFLYLYALVYFL | 17 |
|  | FLYLYALVY | HLA-DRB1*04:04 | 102-116 | EAPFLYLYALVYFLQ | 17 |
|  | FLYLYALVY | HLA-DRB1*04:04 | 103-117 | APFLYLYALVYFLQS | 17 |
|  | FLYLYALVY | HLA-DRB1*04:04 | 100-114 | GLEAPFLYLYALVYF | 18 |
|  | FLYLYALVY | HLA-DRB5*01:01 | 105-119 | FLYLYALVYFLQSIN | 29 |
|  | FLYLYALVY | HLA-DRB5*01:01 | 104-118 | PFLYLYALVYFLQSI | 31 |
|  | FLYLYALVY | HLA-DRB1*04:04 | 105-119 | FLYLYALVYFLQSIN | 40 |
|  | FLYLYALVY | HLA-DRB1*11:01 | 103-117 | APFLYLYALVYFLQS | 42 |
|  | FLYLYALVY | HLA-DRB1*15:01 | 101-115 | LEAPFLYLYALVYFL | 43 |
|  | FLYLYALVY | HLA-DRB1*15:01 | 102-116 | EAPFLYLYALVYFLQ | 43 |
|  | FLYLYALVY | HLA-DRB1*11:01 | 101-115 | LEAPFLYLYALVYFL | 43 |
|  | FLYLYALVY | HLA-DRB1*11:01 | 102-116 | EAPFLYLYALVYFLQ | 43 |
|  | FLYLYALVY | HLA-DRB1*15:01 | 103-117 | APFLYLYALVYFLQS | 44 |
|  | FLYLYALVY | HLA-DRB1*04:05 | 103-117 | APFLYLYALVYFLQS | 45 |
|  | FLYLYALVY | HLA-DRB1*04:05 | 102-116 | EAPFLYLYALVYFLQ | 46 |
|  | FLYLYALVY | HLA-DRB1*04:05 | 101-115 | LEAPFLYLYALVYFL | 47 |
|  | FLYLYALVY | HLA-DRB1*15:01 | 100-114 | GLEAPFLYLYALVYF | 48 |
|  | FLYLYALVY | HLA-DRB1*11:01 | 100-114 | GLEAPFLYLYALVYF | 48 |
|  | FLYLYALVY | HLA-DRB1*04:04 | 104-118 | PFLYLYALVYFLQSI | 49 |
|  | FLYLYALVY | HLA-DPA1*01:03/DPB1*02:01 | 102-116 | EAPFLYLYALVYFLQ | 53 |
|  | FLYLYALVY | HLA-DRB1*07:01 | 101-115 | LEAPFLYLYALVYFL | 62 |
|  | FLYLYALVY | HLA-DPA1*03:01/DPB1*04:02 | 103-117 | APFLYLYALVYFLQS | 71 |
|  | FLYLYALVY | HLA-DRB1*04:05 | 100-114 | GLEAPFLYLYALVYF | 74 |
|  | FLYLYALVY | HLA-DPA1*02:01/DPB1*01:01 | 101-115 | LEAPFLYLYALVYFL | 74 |
|  | FLYLYALVY | HLA-DPA1*01:03/DPB1*02:01 | 101-115 | LEAPFLYLYALVYFL | 81 |
|  | FLYLYALVY | HLA-DRB1*15:01 | 104-118 | PFLYLYALVYFLQSI | 82 |
|  | FLYLYALVY | HLA-DRB1*12:01 | 101-115 | LEAPFLYLYALVYFL | 89 |
|  | FLYLYALVY | HLA-DRB1*11:01 | 105-119 | FLYLYALVYFLQSIN | 92 |
|  | FLYLYALVY | HLA-DPA1*01:03/DPB1*02:01 | 100-114 | GLEAPFLYLYALVYF | 92 |
|  | FLYLYALVY | HLA-DRB1*11:01 | 104-118 | PFLYLYALVYFLQSI | 93 |
|  | FLYLYALVY | HLA-DRB1*15:01 | 105-119 | FLYLYALVYFLQSIN | 100 |
|  | FLYLYALVY | HLA-DRB1*12:01 | 102-116 | EAPFLYLYALVYFLQ | 103 |
|  | FLYLYALVY | HLA-DRB1*12:01 | 103-117 | APFLYLYALVYFLQS | 103 |
|  | FLYLYALVY | HLA-DPA1*02:01/DPB1*01:01 | 100-114 | GLEAPFLYLYALVYF | 114 |
|  | FLYLYALVY | HLA-DRB1*12:01 | 100-114 | GLEAPFLYLYALVYF | 117 |
|  | FLYLYALVY | HLA-DRB1*07:01 | 100-114 | GLEAPFLYLYALVYF | 119 |
|  | FLYLYALVY | HLA-DPA1*03:01/DPB1*04:02 | 102-116 | EAPFLYLYALVYFLQ | 125 |
|  | FLYLYALVY | HLA-DPA1*03:01/DPB1*04:02 | 101-115 | LEAPFLYLYALVYFL | 128 |
|  | FLYLYALVY | HLA-DPA1*03:01/DPB1*04:02 | 100-114 | GLEAPFLYLYALVYF | 131 |
|  | FLYLYALVY | HLA-DRB1*04:01 | 102-116 | EAPFLYLYALVYFLQ | 144 |
|  | FLYLYALVY | HLA-DRB1*04:01 | 103-117 | APFLYLYALVYFLQS | 144 |
|  | FLYLYALVY | HLA-DRB1*04:01 | 101-115 | LEAPFLYLYALVYFL | 162 |
|  | FLYLYALVY | HLA-DRB1*04:01 | 100-114 | GLEAPFLYLYALVYF | 211 |
|  | FLYLYALVY | HLA-DPA1*01/DPB1*04:01 | 102-116 | EAPFLYLYALVYFLQ | 212 |
|  | FLYLYALVY | HLA-DRB1*09:01 | 103-117 | APFLYLYALVYFLQS | 285 |
|  | FLYLYALVY | HLA-DPA1*01/DPB1*04:01 | 101-115 | LEAPFLYLYALVYFL | 309 |
|  | FLYLYALVY | HLA-DPA1*01/DPB1*04:01 | 100-114 | GLEAPFLYLYALVYF | 317 |
|  | FLYLYALVY | HLA-DRB1*09:01 | 102-116 | EAPFLYLYALVYFLQ | 352 |
|  | FLYLYALVY | HLA-DRB1*09:01 | 101-115 | LEAPFLYLYALVYFL | 355 |
|  | FLYLYALVY | HLA-DRB1*09:01 | 100-114 | GLEAPFLYLYALVYF | 430 |
|  | FLYLYALVY | HLA-DQA1*05:01/DQB1*02:01 | 101-115 | LEAPFLYLYALVYFL | 434 |
|  | FLYLYALVY | HLA-DPA1*02:01/DPB1*05:01 | 100-114 | GLEAPFLYLYALVYF | 460 |
|  | FLYLYALVY | HLA-DQA1*05:01/DQB1*03:01 | 101-115 | LEAPFLYLYALVYFL | 490 |
|  | FLYLYALVY | HLA-DRB4*01:01 | 103-117 | APFLYLYALVYFLQS | 557 |
|  | FLYLYALVY | HLA-DQA1*01:01/DQB1*05:01 | 103-117 | APFLYLYALVYFLQS | 750 |
|  | FLYLYALVY | HLA-DQA1*01:01/DQB1*05:01 | 101-115 | LEAPFLYLYALVYFL | 785 |
|  | FLYLYALVY | HLA-DQA1*01:01/DQB1*05:01 | 100-114 | GLEAPFLYLYALVYF | 787 |
|  | FLYLYALVY | HLA-DQA1*05:01/DQB1*02:01 | 100-114 | GLEAPFLYLYALVYF | 824 |
|  | FLYLYALVY | HLA-DQA1*01:01/DQB1*05:01 | 102-116 | EAPFLYLYALVYFLQ | 837 |
|  | FLYLYALVY | HLA-DRB3*01:01 | 100-114 | GLEAPFLYLYALVYF | 848 |
|  | FLYLYALVY | HLA-DRB4*01:01 | 100-114 | GLEAPFLYLYALVYF | 868 |
|  | FLYLYALVY | HLA-DRB4*01:01 | 101-115 | LEAPFLYLYALVYFL | 894 |
|  | FLYLYALVY | HLA-DRB4*01:01 | 102-116 | EAPFLYLYALVYFLQ | 942 |
|  | FLYLYALVY | HLA-DRB1*03:01 | 102-116 | EAPFLYLYALVYFLQ | 983 |
|  | FLYLYALVY | HLA-DRB1*03:01 | 103-117 | APFLYLYALVYFLQS | 985 |
|  | FLYLYALVY | HLA-DRB1*03:01 | 101-115 | LEAPFLYLYALVYFL | 990 |
|  | FLYLYALVY | HLA-DQA1*05:01/DQB1*03:01 | 100-114 | GLEAPFLYLYALVYF | 1769 |
|  | FLYLYALVY | HLA-DRB1*03:01 | 100-114 | GLEAPFLYLYALVYF | 1859 |
|  | FLYLYALVY | HLA-DRB1*13:02 | 102-116 | EAPFLYLYALVYFLQ | 1928 |
|  | FLYLYALVY | HLA-DRB1*13:02 | 101-115 | LEAPFLYLYALVYFL | 1935 |
|  | FLYLYALVY | HLA-DRB1*13:02 | 103-117 | APFLYLYALVYFLQS | 1956 |
|  | FLYLYALVY | HLA-DQA1*03:01/DQB1*03:02 | 101-115 | LEAPFLYLYALVYFL | 2038 |
|  | FLYLYALVY | HLA-DRB1*13:02 | 100-114 | GLEAPFLYLYALVYF | 2137 |
|  | FLYLYALVY | HLA-DQA1*03:01/DQB1*03:02 | 100-114 | GLEAPFLYLYALVYF | 2485 |
|  | FTIGTVTLK | HLA-DRB5*01:01 | 3-17 | LFMRIFTIGTVTLKQ | 252 |
|  | FTIGTVTLK | HLA-DQA1*05:01/DQB1*03:01 | 3-17 | LFMRIFTIGTVTLKQ | 385 |
|  | FTIGTVTLK | HLA-DQA1*05:01/DQB1*03:01 | 4-18 | FMRIFTIGTVTLKQG | 387 |
|  | FTIGTVTLK | HLA-DRB5*01:01 | 5-19 | MRIFTIGTVTLKQGE | 424 |
|  | FTIGTVTLK | HLA-DRB5*01:01 | 6-20 | RIFTIGTVTLKQGEI | 432 |
|  | FTIGTVTLK | HLA-DQA1*05:01/DQB1*03:01 | 5-19 | MRIFTIGTVTLKQGE | 457 |
|  | FTIGTVTLK | HLA-DQA1*05:01/DQB1*03:01 | 6-20 | RIFTIGTVTLKQGEI | 528 |
|  | FTIGTVTLK | HLA-DRB5*01:01 | 7-21 | IFTIGTVTLKQGEIK | 626 |
|  | FTIGTVTLK | HLA-DRB1*01:01 | 8-22 | FTIGTVTLKQGEIKD | 664 |
|  | FTIGTVTLK | HLA-DRB1*12:01 | 3-17 | LFMRIFTIGTVTLKQ | 723 |
|  | FTIGTVTLK | HLA-DRB1*12:01 | 4-18 | FMRIFTIGTVTLKQG | 953 |
|  | FTIGTVTLK | HLA-DQA1*05:01/DQB1*03:01 | 7-21 | IFTIGTVTLKQGEIK | 955 |
|  | FTIGTVTLK | HLA-DQA1*05:01/DQB1*03:01 | 8-22 | FTIGTVTLKQGEIKD | 1229 |
|  | FTIGTVTLK | HLA-DRB1*04:05 | 5-19 | MRIFTIGTVTLKQGE | 1524 |
|  | FTIGTVTLK | HLA-DRB1*09:01 | 5-19 | MRIFTIGTVTLKQGE | 1584 |
|  | FTIGTVTLK | HLA-DRB1*11:01 | 6-20 | RIFTIGTVTLKQGEI | 1640 |
|  | FTIGTVTLK | HLA-DRB1*04:05 | 6-20 | RIFTIGTVTLKQGEI | 1786 |
|  | FTIGTVTLK | HLA-DRB1*09:01 | 6-20 | RIFTIGTVTLKQGEI | 2048 |
|  | FTIGTVTLK | HLA-DRB1*12:01 | 6-20 | RIFTIGTVTLKQGEI | 2152 |
|  | FTIGTVTLK | HLA-DRB1*04:01 | 8-22 | FTIGTVTLKQGEIKD | 2343 |
|  | FTIGTVTLK | HLA-DRB1*03:01 | 3-17 | LFMRIFTIGTVTLKQ | 2694 |
|  | FTIGTVTLK | HLA-DRB1*03:01 | 5-19 | MRIFTIGTVTLKQGE | 2832 |
|  | FTIGTVTLK | HLA-DRB1*03:01 | 4-18 | FMRIFTIGTVTLKQG | 2841 |
|  | FTIGTVTLK | HLA-DRB1*03:01 | 6-20 | RIFTIGTVTLKQGEI | 2951 |
|  | GLEAPFLYL | HLA-DPA1*01:03/DPB1*02:01 | 99-113 | AGLEAPFLYLYALVY | 93 |
|  | GLEAPFLYL | HLA-DPA1*02:01/DPB1*01:01 | 98-112 | AAGLEAPFLYLYALV | 93 |
|  | GLEAPFLYL | HLA-DPA1*01:03/DPB1*02:01 | 98-112 | AAGLEAPFLYLYALV | 111 |
|  | GLEAPFLYL | HLA-DPA1*01:03/DPB1*02:01 | 97-111 | VAAGLEAPFLYLYAL | 132 |
|  | GLEAPFLYL | HLA-DPA1*01:03/DPB1*02:01 | 96-110 | LVAAGLEAPFLYLYA | 140 |
|  | GLEAPFLYL | HLA-DPA1*01:03/DPB1*02:01 | 95-109 | LLVAAGLEAPFLYLY | 142 |
|  | GLEAPFLYL | HLA-DPA1*02:01/DPB1*01:01 | 97-111 | VAAGLEAPFLYLYAL | 151 |
|  | GLEAPFLYL | HLA-DPA1*02:01/DPB1*01:01 | 96-110 | LVAAGLEAPFLYLYA | 161 |
|  | GLEAPFLYL | HLA-DPA1*03:01/DPB1*04:02 | 98-112 | AAGLEAPFLYLYALV | 165 |
|  | GLEAPFLYL | HLA-DPA1*02:01/DPB1*01:01 | 95-109 | LLVAAGLEAPFLYLY | 165 |
|  | GLEAPFLYL | HLA-DPA1*03:01/DPB1*04:02 | 97-111 | VAAGLEAPFLYLYAL | 170 |
|  | GLEAPFLYL | HLA-DPA1*03:01/DPB1*04:02 | 95-109 | LLVAAGLEAPFLYLY | 179 |
|  | GLEAPFLYL | HLA-DPA1*03:01/DPB1*04:02 | 96-110 | LVAAGLEAPFLYLYA | 180 |
|  | GLEAPFLYL | HLA-DPA1*01/DPB1*04:01 | 98-112 | AAGLEAPFLYLYALV | 286 |
|  | GLEAPFLYL | HLA-DPA1*01/DPB1*04:01 | 97-111 | VAAGLEAPFLYLYAL | 344 |
|  | GLEAPFLYL | HLA-DPA1*01/DPB1*04:01 | 96-110 | LVAAGLEAPFLYLYA | 356 |
|  | GLEAPFLYL | HLA-DPA1*01/DPB1*04:01 | 95-109 | LLVAAGLEAPFLYLY | 360 |
|  | GLEAPFLYL | HLA-DPA1*02:01/DPB1*05:01 | 98-112 | AAGLEAPFLYLYALV | 532 |
|  | GLEAPFLYL | HLA-DPA1*02:01/DPB1*05:01 | 96-110 | LVAAGLEAPFLYLYA | 673 |
|  | GLEAPFLYL | HLA-DPA1*02:01/DPB1*05:01 | 95-109 | LLVAAGLEAPFLYLY | 680 |
|  | GLEAPFLYL | HLA-DPA1*02:01/DPB1*05:01 | 97-111 | VAAGLEAPFLYLYAL | 680 |
|  | HSYFTSDYY | HLA-DRB1*15:01 | 201-215 | VVLHSYFTSDYYQLY | 676 |
|  | HSYFTSDYY | HLA-DRB1*15:01 | 202-216 | VLHSYFTSDYYQLYS | 936 |
|  | HSYFTSDYY | HLA-DQA1*01:01/DQB1*05:01 | 201-215 | VVLHSYFTSDYYQLY | 1017 |
|  | HSYFTSDYY | HLA-DQA1*01:01/DQB1*05:01 | 202-216 | VLHSYFTSDYYQLYS | 1244 |
|  | HSYFTSDYY | HLA-DPA1*02:01/DPB1*05:01 | 200-214 | CVVLHSYFTSDYYQL | 1280 |
|  | HSYFTSDYY | HLA-DRB1*15:01 | 203-217 | LHSYFTSDYYQLYST | 1564 |
|  | HSYFTSDYY | HLA-DQA1*01:01/DQB1*05:01 | 200-214 | CVVLHSYFTSDYYQL | 1592 |
|  | HSYFTSDYY | HLA-DQA1*01:01/DQB1*05:01 | 199-213 | DCVVLHSYFTSDYYQ | 1664 |
|  | HSYFTSDYY | HLA-DRB5*01:01 | 201-215 | VVLHSYFTSDYYQLY | 1841 |
|  | HSYFTSDYY | HLA-DRB1*15:01 | 204-218 | HSYFTSDYYQLYSTQ | 1923 |
|  | HSYFTSDYY | HLA-DRB4*01:01 | 201-215 | VVLHSYFTSDYYQLY | 2817 |
|  | HVTFFIYNK | HLA-DRB1*15:01 | 222-236 | DTGVEHVTFFIYNKI | 553 |
|  | HVTFFIYNK | HLA-DRB1*04:05 | 224-238 | GVEHVTFFIYNKIVD | 1121 |
|  | HVTFFIYNK | HLA-DRB1*04:01 | 225-239 | VEHVTFFIYNKIVDE | 1667 |
|  | HVTFFIYNK | HLA-DQA1*01:01/DQB1*05:01 | 223-237 | TGVEHVTFFIYNKIV | 2033 |
|  | HVTFFIYNK | HLA-DQA1*01:01/DQB1*05:01 | 222-236 | DTGVEHVTFFIYNKI | 2119 |
|  | HVTFFIYNK | HLA-DRB1*04:05 | 223-237 | TGVEHVTFFIYNKIV | 2217 |
|  | HVTFFIYNK | HLA-DRB1*04:05 | 222-236 | DTGVEHVTFFIYNKI | 2356 |
|  | HVTFFIYNK | HLA-DRB1*04:01 | 224-238 | GVEHVTFFIYNKIVD | 2886 |
|  | HVTFFIYNK | HLA-DRB5*01:01 | 222-236 | DTGVEHVTFFIYNKI | 2995 |
|  | LKKRWQLAL | HLA-DRB1*01:01 | 63-77 | ITLKKRWQLALSKGV | 39 |
|  | LKKRWQLAL | HLA-DRB1*11:01 | 63-77 | ITLKKRWQLALSKGV | 121 |
|  | LKKRWQLAL | HLA-DRB1*15:01 | 63-77 | ITLKKRWQLALSKGV | 290 |
|  | LKKRWQLAL | HLA-DPA1*02:01/DPB1*01:01 | 61-75 | KIITLKKRWQLALSK | 304 |
|  | LKKRWQLAL | HLA-DPA1*02:01/DPB1*01:01 | 60-74 | SKIITLKKRWQLALS | 307 |
|  | LKKRWQLAL | HLA-DPA1*02:01/DPB1*01:01 | 63-77 | ITLKKRWQLALSKGV | 328 |
|  | LKKRWQLAL | HLA-DPA1*02:01/DPB1*01:01 | 62-76 | IITLKKRWQLALSKG | 330 |
|  | LKKRWQLAL | HLA-DPA1*01:03/DPB1*02:01 | 61-75 | KIITLKKRWQLALSK | 399 |
|  | LKKRWQLAL | HLA-DPA1*01:03/DPB1*02:01 | 60-74 | SKIITLKKRWQLALS | 419 |
|  | LKKRWQLAL | HLA-DPA1*03:01/DPB1*04:02 | 61-75 | KIITLKKRWQLALSK | 420 |
|  | LKKRWQLAL | HLA-DPA1*01:03/DPB1*02:01 | 62-76 | IITLKKRWQLALSKG | 426 |
|  | LKKRWQLAL | HLA-DPA1*03:01/DPB1*04:02 | 62-76 | IITLKKRWQLALSKG | 428 |
|  | LKKRWQLAL | HLA-DPA1*03:01/DPB1*04:02 | 60-74 | SKIITLKKRWQLALS | 429 |
|  | LKKRWQLAL | HLA-DPA1*03:01/DPB1*04:02 | 63-77 | ITLKKRWQLALSKGV | 434 |
|  | LKKRWQLAL | HLA-DPA1*01:03/DPB1*02:01 | 63-77 | ITLKKRWQLALSKGV | 454 |
|  | LKKRWQLAL | HLA-DRB1*01:01 | 61-75 | KIITLKKRWQLALSK | 634 |
|  | LKKRWQLAL | HLA-DPA1*02:01/DPB1*01:01 | 64-78 | TLKKRWQLALSKGVH | 676 |
|  | LKKRWQLAL | HLA-DRB4*01:01 | 63-77 | ITLKKRWQLALSKGV | 707 |
|  | LKKRWQLAL | HLA-DRB1*13:02 | 63-77 | ITLKKRWQLALSKGV | 759 |
|  | LKKRWQLAL | HLA-DRB1*12:01 | 63-77 | ITLKKRWQLALSKGV | 799 |
|  | LKKRWQLAL | HLA-DPA1*02:01/DPB1*01:01 | 65-79 | LKKRWQLALSKGVHF | 840 |
|  | LKKRWQLAL | HLA-DRB1*08:02 | 60-74 | SKIITLKKRWQLALS | 846 |
|  | LKKRWQLAL | HLA-DPA1*01:03/DPB1*02:01 | 65-79 | LKKRWQLALSKGVHF | 857 |
|  | LKKRWQLAL | HLA-DRB1*08:02 | 62-76 | IITLKKRWQLALSKG | 876 |
|  | LKKRWQLAL | HLA-DRB1*08:02 | 61-75 | KIITLKKRWQLALSK | 895 |
|  | LKKRWQLAL | HLA-DRB1*08:02 | 63-77 | ITLKKRWQLALSKGV | 904 |
|  | LKKRWQLAL | HLA-DPA1*01/DPB1*04:01 | 61-75 | KIITLKKRWQLALSK | 925 |
|  | LKKRWQLAL | HLA-DPA1*01/DPB1*04:01 | 60-74 | SKIITLKKRWQLALS | 936 |
|  | LKKRWQLAL | HLA-DRB1*09:01 | 60-74 | SKIITLKKRWQLALS | 946 |
|  | LKKRWQLAL | HLA-DRB1*09:01 | 61-75 | KIITLKKRWQLALSK | 982 |
|  | LKKRWQLAL | HLA-DPA1*01/DPB1*04:01 | 62-76 | IITLKKRWQLALSKG | 998 |
|  | LKKRWQLAL | HLA-DPA1*03:01/DPB1*04:02 | 64-78 | TLKKRWQLALSKGVH | 1037 |
|  | LKKRWQLAL | HLA-DPA1*01/DPB1*04:01 | 63-77 | ITLKKRWQLALSKGV | 1054 |
|  | LKKRWQLAL | HLA-DPA1*03:01/DPB1*04:02 | 65-79 | LKKRWQLALSKGVHF | 1072 |
|  | LKKRWQLAL | HLA-DRB1*09:01 | 62-76 | IITLKKRWQLALSKG | 1144 |
|  | LKKRWQLAL | HLA-DPA1*01:03/DPB1*02:01 | 64-78 | TLKKRWQLALSKGVH | 1202 |
|  | LKKRWQLAL | HLA-DPA1*02:01/DPB1*05:01 | 60-74 | SKIITLKKRWQLALS | 1332 |
|  | LKKRWQLAL | HLA-DPA1*02:01/DPB1*05:01 | 61-75 | KIITLKKRWQLALSK | 1495 |
|  | LKKRWQLAL | HLA-DPA1*02:01/DPB1*05:01 | 62-76 | IITLKKRWQLALSKG | 1512 |
|  | LKKRWQLAL | HLA-DPA1*02:01/DPB1*05:01 | 63-77 | ITLKKRWQLALSKGV | 1579 |
|  | LKKRWQLAL | HLA-DPA1*01/DPB1*04:01 | 65-79 | LKKRWQLALSKGVHF | 1837 |
|  | LKKRWQLAL | HLA-DRB3*01:01 | 63-77 | ITLKKRWQLALSKGV | 1838 |
|  | LKKRWQLAL | HLA-DRB1*08:02 | 65-79 | LKKRWQLALSKGVHF | 1968 |
|  | LKKRWQLAL | HLA-DPA1*01/DPB1*04:01 | 64-78 | TLKKRWQLALSKGVH | 2762 |
|  | LLLLFVTVY | HLA-DRB1*01:01 | 81-95 | CNLLLLFVTVYSHLL | 4 |
|  | LLLLFVTVY | HLA-DRB1*04:04 | 78-92 | HFVCNLLLLFVTVYS | 39 |
|  | LLLLFVTVY | HLA-DRB1*01:01 | 78-92 | HFVCNLLLLFVTVYS | 59 |
|  | LLLLFVTVY | HLA-DRB1*01:01 | 80-94 | VCNLLLLFVTVYSHL | 67 |
|  | LLLLFVTVY | HLA-DRB1*01:01 | 79-93 | FVCNLLLLFVTVYSH | 68 |
|  | LLLLFVTVY | HLA-DRB1*15:01 | 78-92 | HFVCNLLLLFVTVYS | 86 |
|  | LLLLFVTVY | HLA-DRB1*12:01 | 78-92 | HFVCNLLLLFVTVYS | 107 |
|  | LLLLFVTVY | HLA-DRB1*12:01 | 79-93 | FVCNLLLLFVTVYSH | 116 |
|  | LLLLFVTVY | HLA-DRB1*04:05 | 78-92 | HFVCNLLLLFVTVYS | 120 |
|  | LLLLFVTVY | HLA-DRB1*12:01 | 80-94 | VCNLLLLFVTVYSHL | 128 |
|  | LLLLFVTVY | HLA-DRB1*04:01 | 78-92 | HFVCNLLLLFVTVYS | 131 |
|  | LLLLFVTVY | HLA-DRB1*12:01 | 81-95 | CNLLLLFVTVYSHLL | 134 |
|  | LLLLFVTVY | HLA-DRB1*12:01 | 82-96 | NLLLLFVTVYSHLLL | 250 |
|  | LLLLFVTVY | HLA-DRB1*12:01 | 83-97 | LLLLFVTVYSHLLLV | 324 |
|  | LLLLFVTVY | HLA-DRB4*01:01 | 80-94 | VCNLLLLFVTVYSHL | 451 |
|  | LLLLFVTVY | HLA-DRB4*01:01 | 81-95 | CNLLLLFVTVYSHLL | 466 |
|  | LLLLFVTVY | HLA-DRB4*01:01 | 79-93 | FVCNLLLLFVTVYSH | 515 |
|  | LLLLFVTVY | HLA-DRB4*01:01 | 82-96 | NLLLLFVTVYSHLLL | 548 |
|  | LLLLFVTVY | HLA-DRB5*01:01 | 78-92 | HFVCNLLLLFVTVYS | 971 |
|  | LLLLFVTVY | HLA-DQA1*01:01/DQB1*05:01 | 78-92 | HFVCNLLLLFVTVYS | 1251 |
|  | LLLLFVTVY | HLA-DQA1*05:01/DQB1*02:01 | 81-95 | CNLLLLFVTVYSHLL | 1320 |
|  | LLLLFVTVY | HLA-DQA1*01:01/DQB1*05:01 | 79-93 | FVCNLLLLFVTVYSH | 1433 |
|  | LLLLFVTVY | HLA-DQA1*01:01/DQB1*05:01 | 80-94 | VCNLLLLFVTVYSHL | 1508 |
|  | LLLLFVTVY | HLA-DQA1*05:01/DQB1*02:01 | 80-94 | VCNLLLLFVTVYSHL | 1513 |
|  | LLLLFVTVY | HLA-DQA1*05:01/DQB1*02:01 | 78-92 | HFVCNLLLLFVTVYS | 1602 |
|  | LLLLFVTVY | HLA-DQA1*01:01/DQB1*05:01 | 81-95 | CNLLLLFVTVYSHLL | 1709 |
|  | LLLLFVTVY | HLA-DQA1*05:01/DQB1*02:01 | 79-93 | FVCNLLLLFVTVYSH | 1775 |
|  | LLLLFVTVY | HLA-DRB1*03:01 | 81-95 | CNLLLLFVTVYSHLL | 1792 |
|  | LLLLFVTVY | HLA-DRB1*03:01 | 80-94 | VCNLLLLFVTVYSHL | 2020 |
|  | LLLLFVTVY | HLA-DQA1*04:01/DQB1*04:02 | 79-93 | FVCNLLLLFVTVYSH | 2504 |
|  | LLLLFVTVY | HLA-DQA1*03:01/DQB1*03:02 | 79-93 | FVCNLLLLFVTVYSH | 2806 |
|  | LYALVYFLQ | HLA-DPA1*01:03/DPB1*02:01 | 103-117 | APFLYLYALVYFLQS | 16 |
|  | LYALVYFLQ | HLA-DPA1*01/DPB1*04:01 | 103-117 | APFLYLYALVYFLQS | 27 |
|  | LYALVYFLQ | HLA-DPA1*02:01/DPB1*01:01 | 104-118 | PFLYLYALVYFLQSI | 28 |
|  | LYALVYFLQ | HLA-DPA1*02:01/DPB1*01:01 | 105-119 | FLYLYALVYFLQSIN | 28 |
|  | LYALVYFLQ | HLA-DPA1*02:01/DPB1*01:01 | 103-117 | APFLYLYALVYFLQS | 29 |
|  | LYALVYFLQ | HLA-DPA1*02:01/DPB1*01:01 | 106-120 | LYLYALVYFLQSINF | 35 |
|  | LYALVYFLQ | HLA-DPA1*02:01/DPB1*05:01 | 103-117 | APFLYLYALVYFLQS | 122 |
|  | LYALVYFLQ | HLA-DPA1*02:01/DPB1*05:01 | 104-118 | PFLYLYALVYFLQSI | 123 |
|  | LYALVYFLQ | HLA-DPA1*02:01/DPB1*05:01 | 105-119 | FLYLYALVYFLQSIN | 130 |
|  | LYALVYFLQ | HLA-DPA1*02:01/DPB1*05:01 | 106-120 | LYLYALVYFLQSINF | 161 |
|  | LYALVYFLQ | HLA-DRB1*03:01 | 108-122 | LYALVYFLQSINFVR | 247 |
|  | LYALVYFLQ | HLA-DPA1*02:01/DPB1*05:01 | 107-121 | YLYALVYFLQSINFV | 272 |
|  | LYALVYFLQ | HLA-DPA1*02:01/DPB1*05:01 | 108-122 | LYALVYFLQSINFVR | 315 |
|  | LYALVYFLQ | HLA-DQA1*01:02/DQB1*06:02 | 104-118 | PFLYLYALVYFLQSI | 1000 |
|  | LYALVYFLQ | HLA-DQA1*01:02/DQB1*06:02 | 103-117 | APFLYLYALVYFLQS | 1002 |
|  | LYALVYFLQ | HLA-DQA1*01:02/DQB1*06:02 | 105-119 | FLYLYALVYFLQSIN | 1100 |
|  | LYALVYFLQ | HLA-DQA1*01:02/DQB1*06:02 | 106-120 | LYLYALVYFLQSINF | 1221 |
|  | LYALVYFLQ | HLA-DQA1*04:01/DQB1*04:02 | 106-120 | LYLYALVYFLQSINF | 1428 |
|  | LYALVYFLQ | HLA-DQA1*01:02/DQB1*06:02 | 107-121 | YLYALVYFLQSINFV | 2137 |
|  | LYALVYFLQ | HLA-DQA1*01:02/DQB1*06:02 | 108-122 | LYALVYFLQSINFVR | 2792 |
|  | LYLYALVYF | HLA-DPA1*02:01/DPB1*05:01 | 101-115 | LEAPFLYLYALVYFL | 297 |
|  | LYLYALVYF | HLA-DRB3*01:01 | 101-115 | LEAPFLYLYALVYFL | 431 |
|  | LYLYALVYF | HLA-DQA1*01:01/DQB1*05:01 | 104-118 | PFLYLYALVYFLQSI | 517 |
|  | LYLYALVYF | HLA-DQA1*04:01/DQB1*04:02 | 101-115 | LEAPFLYLYALVYFL | 1292 |
|  | LYLYALVYF | HLA-DRB1*08:02 | 105-119 | FLYLYALVYFLQSIN | 1618 |
|  | LYLYALVYF | HLA-DRB1*08:02 | 103-117 | APFLYLYALVYFLQS | 2611 |
|  | LYLYALVYF | HLA-DRB1*08:02 | 102-116 | EAPFLYLYALVYFLQ | 2692 |
|  | LYLYALVYF | HLA-DRB1*08:02 | 104-118 | PFLYLYALVYFLQSI | 2875 |
|  | LYLYALVYF | HLA-DRB1*08:02 | 101-115 | LEAPFLYLYALVYFL | 2886 |
|  | STDTGVEHV | HLA-DPA1*02:01/DPB1*01:01 | 219-233 | LSTDTGVEHVTFFIY | 105 |
|  | STDTGVEHV | HLA-DPA1*03:01/DPB1*04:02 | 219-233 | LSTDTGVEHVTFFIY | 1214 |
|  | STDTGVEHV | HLA-DQA1*03:01/DQB1*03:02 | 216-230 | STQLSTDTGVEHVTF | 1427 |
|  | STDTGVEHV | HLA-DQA1*03:01/DQB1*03:02 | 217-231 | TQLSTDTGVEHVTFF | 1538 |
|  | STDTGVEHV | HLA-DQA1*03:01/DQB1*03:02 | 218-232 | QLSTDTGVEHVTFFI | 1548 |
|  | STDTGVEHV | HLA-DQA1*03:01/DQB1*03:02 | 215-229 | YSTQLSTDTGVEHVT | 1555 |
|  | STDTGVEHV | HLA-DRB1*07:01 | 218-232 | QLSTDTGVEHVTFFI | 1873 |
|  | STDTGVEHV | HLA-DQA1*01:02/DQB1*06:02 | 216-230 | STQLSTDTGVEHVTF | 2202 |
|  | STDTGVEHV | HLA-DQA1*01:02/DQB1*06:02 | 217-231 | TQLSTDTGVEHVTFF | 2421 |
|  | STDTGVEHV | HLA-DQA1*03:01/DQB1*03:02 | 220-234 | STDTGVEHVTFFIYN | 2780 |
|  | STDTGVEHV | HLA-DQA1*03:01/DQB1*03:02 | 219-233 | LSTDTGVEHVTFFIY | 2914 |
|  | VHFVCNLLL | HLA-DRB1*04:04 | 74-88 | SKGVHFVCNLLLLFV | 16 |
|  | VHFVCNLLL | HLA-DRB1*04:04 | 73-87 | LSKGVHFVCNLLLLF | 17 |
|  | VHFVCNLLL | HLA-DRB1*04:04 | 75-89 | KGVHFVCNLLLLFVT | 17 |
|  | VHFVCNLLL | HLA-DRB1*04:04 | 72-86 | ALSKGVHFVCNLLLL | 18 |
|  | VHFVCNLLL | HLA-DRB1*07:01 | 74-88 | SKGVHFVCNLLLLFV | 32 |
|  | VHFVCNLLL | HLA-DRB1*07:01 | 75-89 | KGVHFVCNLLLLFVT | 32 |
|  | VHFVCNLLL | HLA-DRB1*07:01 | 73-87 | LSKGVHFVCNLLLLF | 34 |
|  | VHFVCNLLL | HLA-DRB1*07:01 | 72-86 | ALSKGVHFVCNLLLL | 35 |
|  | VHFVCNLLL | HLA-DRB1*04:04 | 77-91 | VHFVCNLLLLFVTVY | 44 |
|  | VHFVCNLLL | HLA-DRB1*04:04 | 76-90 | GVHFVCNLLLLFVTV | 48 |
|  | VHFVCNLLL | HLA-DRB1*01:01 | 75-89 | KGVHFVCNLLLLFVT | 48 |
|  | VHFVCNLLL | HLA-DRB1*01:01 | 74-88 | SKGVHFVCNLLLLFV | 49 |
|  | VHFVCNLLL | HLA-DPA1*03:01/DPB1*04:02 | 72-86 | ALSKGVHFVCNLLLL | 55 |
|  | VHFVCNLLL | HLA-DRB1*01:01 | 73-87 | LSKGVHFVCNLLLLF | 67 |
|  | VHFVCNLLL | HLA-DPA1*02:01/DPB1*01:01 | 72-86 | ALSKGVHFVCNLLLL | 91 |
|  | VHFVCNLLL | HLA-DRB1*07:01 | 76-90 | GVHFVCNLLLLFVTV | 98 |
|  | VHFVCNLLL | HLA-DRB1*01:01 | 72-86 | ALSKGVHFVCNLLLL | 98 |
|  | VHFVCNLLL | HLA-DRB1*07:01 | 77-91 | VHFVCNLLLLFVTVY | 99 |
|  | VHFVCNLLL | HLA-DRB1*15:01 | 74-88 | SKGVHFVCNLLLLFV | 141 |
|  | VHFVCNLLL | HLA-DRB1*15:01 | 75-89 | KGVHFVCNLLLLFVT | 150 |
|  | VHFVCNLLL | HLA-DRB1*15:01 | 73-87 | LSKGVHFVCNLLLLF | 160 |
|  | VHFVCNLLL | HLA-DRB1*04:05 | 74-88 | SKGVHFVCNLLLLFV | 167 |
|  | VHFVCNLLL | HLA-DRB1*04:05 | 75-89 | KGVHFVCNLLLLFVT | 176 |
|  | VHFVCNLLL | HLA-DRB1*15:01 | 72-86 | ALSKGVHFVCNLLLL | 185 |
|  | VHFVCNLLL | HLA-DRB1*04:05 | 73-87 | LSKGVHFVCNLLLLF | 189 |
|  | VHFVCNLLL | HLA-DRB1*11:01 | 74-88 | SKGVHFVCNLLLLFV | 197 |
|  | VHFVCNLLL | HLA-DRB1*11:01 | 75-89 | KGVHFVCNLLLLFVT | 200 |
|  | VHFVCNLLL | HLA-DRB1*11:01 | 73-87 | LSKGVHFVCNLLLLF | 204 |
|  | VHFVCNLLL | HLA-DRB1*04:05 | 72-86 | ALSKGVHFVCNLLLL | 242 |
|  | VHFVCNLLL | HLA-DRB1*15:01 | 77-91 | VHFVCNLLLLFVTVY | 245 |
|  | VHFVCNLLL | HLA-DRB1*15:01 | 76-90 | GVHFVCNLLLLFVTV | 294 |
|  | VHFVCNLLL | HLA-DRB1*11:01 | 72-86 | ALSKGVHFVCNLLLL | 304 |
|  | VHFVCNLLL | HLA-DRB4*01:01 | 74-88 | SKGVHFVCNLLLLFV | 344 |
|  | VHFVCNLLL | HLA-DRB4*01:01 | 75-89 | KGVHFVCNLLLLFVT | 344 |
|  | VHFVCNLLL | HLA-DRB1*13:02 | 74-88 | SKGVHFVCNLLLLFV | 387 |
|  | VHFVCNLLL | HLA-DRB1*13:02 | 75-89 | KGVHFVCNLLLLFVT | 390 |
|  | VHFVCNLLL | HLA-DRB1*13:02 | 73-87 | LSKGVHFVCNLLLLF | 401 |
|  | VHFVCNLLL | HLA-DRB5*01:01 | 74-88 | SKGVHFVCNLLLLFV | 454 |
|  | VHFVCNLLL | HLA-DRB5*01:01 | 75-89 | KGVHFVCNLLLLFVT | 458 |
|  | VHFVCNLLL | HLA-DRB1*04:01 | 74-88 | SKGVHFVCNLLLLFV | 467 |
|  | VHFVCNLLL | HLA-DRB5*01:01 | 73-87 | LSKGVHFVCNLLLLF | 474 |
|  | VHFVCNLLL | HLA-DRB1*13:02 | 72-86 | ALSKGVHFVCNLLLL | 490 |
|  | VHFVCNLLL | HLA-DRB1*04:01 | 75-89 | KGVHFVCNLLLLFVT | 491 |
|  | VHFVCNLLL | HLA-DRB1*04:01 | 73-87 | LSKGVHFVCNLLLLF | 507 |
|  | VHFVCNLLL | HLA-DRB1*03:01 | 73-87 | LSKGVHFVCNLLLLF | 510 |
|  | VHFVCNLLL | HLA-DRB4*01:01 | 73-87 | LSKGVHFVCNLLLLF | 537 |
|  | VHFVCNLLL | HLA-DPA1*02:01/DPB1*05:01 | 72-86 | ALSKGVHFVCNLLLL | 661 |
|  | VHFVCNLLL | HLA-DRB5*01:01 | 72-86 | ALSKGVHFVCNLLLL | 709 |
|  | VHFVCNLLL | HLA-DRB1*04:01 | 72-86 | ALSKGVHFVCNLLLL | 761 |
|  | VHFVCNLLL | HLA-DRB4*01:01 | 72-86 | ALSKGVHFVCNLLLL | 770 |
|  | VHFVCNLLL | HLA-DRB1*09:01 | 74-88 | SKGVHFVCNLLLLFV | 925 |
|  | VHFVCNLLL | HLA-DRB1*09:01 | 75-89 | KGVHFVCNLLLLFVT | 936 |
|  | VHFVCNLLL | HLA-DRB1*09:01 | 73-87 | LSKGVHFVCNLLLLF | 1223 |
|  | VHFVCNLLL | HLA-DRB1*12:01 | 73-87 | LSKGVHFVCNLLLLF | 1261 |
|  | VHFVCNLLL | HLA-DRB1*09:01 | 72-86 | ALSKGVHFVCNLLLL | 1415 |
|  | VHFVCNLLL | HLA-DRB1*08:02 | 74-88 | SKGVHFVCNLLLLFV | 1847 |
|  | YLYALVYFL | HLA-DRB1*04:05 | 105-119 | FLYLYALVYFLQSIN | 31 |
|  | YLYALVYFL | HLA-DPA1*02:01/DPB1*01:01 | 102-116 | EAPFLYLYALVYFLQ | 37 |
|  | YLYALVYFL | HLA-DRB1*01:01 | 107-121 | YLYALVYFLQSINFV | 42 |
|  | YLYALVYFL | HLA-DRB1*01:01 | 106-120 | LYLYALVYFLQSINF | 49 |
|  | YLYALVYFL | HLA-DRB1*07:01 | 103-117 | APFLYLYALVYFLQS | 62 |
|  | YLYALVYFL | HLA-DRB1*07:01 | 102-116 | EAPFLYLYALVYFLQ | 63 |
|  | YLYALVYFL | HLA-DRB1*04:05 | 104-118 | PFLYLYALVYFLQSI | 66 |
|  | YLYALVYFL | HLA-DRB1*07:01 | 105-119 | FLYLYALVYFLQSIN | 81 |
|  | YLYALVYFL | HLA-DRB1*07:01 | 104-118 | PFLYLYALVYFLQSI | 82 |
|  | YLYALVYFL | HLA-DRB1*12:01 | 107-121 | YLYALVYFLQSINFV | 121 |
|  | YLYALVYFL | HLA-DPA1*02:01/DPB1*05:01 | 102-116 | EAPFLYLYALVYFLQ | 128 |
|  | YLYALVYFL | HLA-DRB1*07:01 | 106-120 | LYLYALVYFLQSINF | 146 |
|  | YLYALVYFL | HLA-DRB1*12:01 | 104-118 | PFLYLYALVYFLQSI | 156 |
|  | YLYALVYFL | HLA-DRB5*01:01 | 106-120 | LYLYALVYFLQSINF | 174 |
|  | YLYALVYFL | HLA-DRB5*01:01 | 107-121 | YLYALVYFLQSINFV | 180 |
|  | YLYALVYFL | HLA-DRB1*12:01 | 105-119 | FLYLYALVYFLQSIN | 193 |
|  | YLYALVYFL | HLA-DRB1*04:01 | 105-119 | FLYLYALVYFLQSIN | 205 |
|  | YLYALVYFL | HLA-DRB1*04:01 | 104-118 | PFLYLYALVYFLQSI | 225 |
|  | YLYALVYFL | HLA-DRB1*04:01 | 106-120 | LYLYALVYFLQSINF | 286 |
|  | YLYALVYFL | HLA-DRB1*09:01 | 105-119 | FLYLYALVYFLQSIN | 422 |
|  | YLYALVYFL | HLA-DQA1*05:01/DQB1*03:01 | 102-116 | EAPFLYLYALVYFLQ | 433 |
|  | YLYALVYFL | HLA-DRB3*01:01 | 102-116 | EAPFLYLYALVYFLQ | 434 |
|  | YLYALVYFL | HLA-DRB1*09:01 | 104-118 | PFLYLYALVYFLQSI | 434 |
|  | YLYALVYFL | HLA-DRB3*01:01 | 103-117 | APFLYLYALVYFLQS | 437 |
|  | YLYALVYFL | HLA-DRB3*01:01 | 104-118 | PFLYLYALVYFLQSI | 437 |
|  | YLYALVYFL | HLA-DQA1*05:01/DQB1*02:01 | 102-116 | EAPFLYLYALVYFLQ | 439 |
|  | YLYALVYFL | HLA-DQA1*05:01/DQB1*02:01 | 103-117 | APFLYLYALVYFLQS | 449 |
|  | YLYALVYFL | HLA-DQA1*05:01/DQB1*03:01 | 103-117 | APFLYLYALVYFLQS | 457 |
|  | YLYALVYFL | HLA-DRB1*12:01 | 106-120 | LYLYALVYFLQSINF | 476 |
|  | YLYALVYFL | HLA-DQA1*05:01/DQB1*02:01 | 107-121 | YLYALVYFLQSINFV | 519 |
|  | YLYALVYFL | HLA-DQA1*05:01/DQB1*02:01 | 104-118 | PFLYLYALVYFLQSI | 522 |
|  | YLYALVYFL | HLA-DQA1*05:01/DQB1*03:01 | 104-118 | PFLYLYALVYFLQSI | 539 |
|  | YLYALVYFL | HLA-DQA1*05:01/DQB1*03:01 | 105-119 | FLYLYALVYFLQSIN | 544 |
|  | YLYALVYFL | HLA-DRB3*01:01 | 105-119 | FLYLYALVYFLQSIN | 557 |
|  | YLYALVYFL | HLA-DQA1*05:01/DQB1*02:01 | 105-119 | FLYLYALVYFLQSIN | 567 |
|  | YLYALVYFL | HLA-DQA1*04:01/DQB1*04:02 | 102-116 | EAPFLYLYALVYFLQ | 843 |
|  | YLYALVYFL | HLA-DQA1*04:01/DQB1*04:02 | 104-118 | PFLYLYALVYFLQSI | 858 |
|  | YLYALVYFL | HLA-DQA1*04:01/DQB1*04:02 | 103-117 | APFLYLYALVYFLQS | 867 |
|  | YLYALVYFL | HLA-DQA1*04:01/DQB1*04:02 | 105-119 | FLYLYALVYFLQSIN | 1018 |
|  | YLYALVYFL | HLA-DQA1*05:01/DQB1*03:01 | 107-121 | YLYALVYFLQSINFV | 1023 |
|  | YLYALVYFL | HLA-DQA1*05:01/DQB1*03:01 | 106-120 | LYLYALVYFLQSINF | 1051 |
|  | YLYALVYFL | HLA-DQA1*05:01/DQB1*02:01 | 106-120 | LYLYALVYFLQSINF | 1056 |
|  | YLYALVYFL | HLA-DRB3*01:01 | 107-121 | YLYALVYFLQSINFV | 1079 |
|  | YLYALVYFL | HLA-DQA1*01:02/DQB1*06:02 | 102-116 | EAPFLYLYALVYFLQ | 1175 |
|  | YLYALVYFL | HLA-DRB1*03:01 | 105-119 | FLYLYALVYFLQSIN | 1284 |
|  | YLYALVYFL | HLA-DRB3*01:01 | 106-120 | LYLYALVYFLQSINF | 1307 |
|  | YLYALVYFL | HLA-DRB1*03:01 | 104-118 | PFLYLYALVYFLQSI | 1325 |
|  | YLYALVYFL | HLA-DQA1*03:01/DQB1*03:02 | 104-118 | PFLYLYALVYFLQSI | 1407 |
|  | YLYALVYFL | HLA-DQA1*03:01/DQB1*03:02 | 103-117 | APFLYLYALVYFLQS | 1849 |
|  | YLYALVYFL | HLA-DQA1*03:01/DQB1*03:02 | 102-116 | EAPFLYLYALVYFLQ | 2247 |
|  | YLYALVYFL | HLA-DQA1*04:01/DQB1*04:02 | 107-121 | YLYALVYFLQSINFV | 2398 |
|  | YLYALVYFL | HLA-DRB1*13:02 | 106-120 | LYLYALVYFLQSINF | 2528 |
|  | YQIGGYTEK | HLA-DQA1*05:01/DQB1*03:01 | 181-195 | EHDYQIGGYTEKWES | 1161 |
|  | YQIGGYTEK | HLA-DQA1*05:01/DQB1*03:01 | 182-196 | HDYQIGGYTEKWESG | 1214 |
|  | YQIGGYTEK | HLA-DPA1*01:03/DPB1*02:01 | 182-196 | HDYQIGGYTEKWESG | 2303 |
|  | YQIGGYTEK | HLA-DRB1*01:01 | 182-196 | HDYQIGGYTEKWESG | 2329 |
|  | YQIGGYTEK | HLA-DPA1*01:03/DPB1*02:01 | 181-195 | EHDYQIGGYTEKWES | 2833 |
|  | YQIGGYTEK | HLA-DPA1*01:03/DPB1*02:01 | 179-193 | ISEHDYQIGGYTEKW | 2892 |
| **Envelope protein** | | | | | |
| **No** | **Core sequence** | **MHC II alleles** | **position** | **Peptide** | **IC50 value** |
|  | FLAFVVFLL | HLA-DPA1*02:01/DPB1*01:01 | 17-31 | VLLFLAFVVFLLVTL | 5 |
|  | FLAFVVFLL | HLA-DPA1*02:01/DPB1*01:01 | 18-32 | LLFLAFVVFLLVTLA | 5 |
|  | FLAFVVFLL | HLA-DPA1*01:03/DPB1*02:01 | 15-29 | NSVLLFLAFVVFLLV | 5 |
|  | FLAFVVFLL | HLA-DPA1*02:01/DPB1*01:01 | 15-29 | NSVLLFLAFVVFLLV | 6 |
|  | FLAFVVFLL | HLA-DPA1*02:01/DPB1*01:01 | 16-30 | SVLLFLAFVVFLLVT | 6 |
|  | FLAFVVFLL | HLA-DPA1*03:01/DPB1*04:02 | 15-29 | NSVLLFLAFVVFLLV | 10 |
|  | FLAFVVFLL | HLA-DPA1*01/DPB1*04:01 | 15-29 | NSVLLFLAFVVFLLV | 14 |
|  | FLAFVVFLL | HLA-DPA1*02:01/DPB1*01:01 | 20-34 | FLAFVVFLLVTLAIL | 14 |
|  | FLAFVVFLL | HLA-DRB1*01:01 | 18-32 | LLFLAFVVFLLVTLA | 20 |
|  | FLAFVVFLL | HLA-DRB1*04:04 | 15-29 | NSVLLFLAFVVFLLV | 21 |
|  | FLAFVVFLL | HLA-DRB1*01:01 | 15-29 | NSVLLFLAFVVFLLV | 22 |
|  | FLAFVVFLL | HLA-DRB1*04:04 | 17-31 | VLLFLAFVVFLLVTL | 23 |
|  | FLAFVVFLL | HLA-DRB1*04:04 | 18-32 | LLFLAFVVFLLVTLA | 23 |
|  | FLAFVVFLL | HLA-DRB1*01:01 | 17-31 | VLLFLAFVVFLLVTL | 24 |
|  | FLAFVVFLL | HLA-DRB1*04:04 | 16-30 | SVLLFLAFVVFLLVT | 25 |
|  | FLAFVVFLL | HLA-DRB1*01:01 | 16-30 | SVLLFLAFVVFLLVT | 27 |
|  | FLAFVVFLL | HLA-DRB1*04:05 | 17-31 | VLLFLAFVVFLLVTL | 30 |
|  | FLAFVVFLL | HLA-DRB1*04:05 | 18-32 | LLFLAFVVFLLVTLA | 31 |
|  | FLAFVVFLL | HLA-DRB1*04:05 | 16-30 | SVLLFLAFVVFLLVT | 37 |
|  | FLAFVVFLL | HLA-DRB1*04:05 | 15-29 | NSVLLFLAFVVFLLV | 38 |
|  | FLAFVVFLL | HLA-DRB1*04:04 | 20-34 | FLAFVVFLLVTLAIL | 47 |
|  | FLAFVVFLL | HLA-DRB1*04:04 | 19-33 | LFLAFVVFLLVTLAI | 48 |
|  | FLAFVVFLL | HLA-DPA1*02:01/DPB1*05:01 | 17-31 | VLLFLAFVVFLLVTL | 55 |
|  | FLAFVVFLL | HLA-DPA1*02:01/DPB1*05:01 | 16-30 | SVLLFLAFVVFLLVT | 57 |
|  | FLAFVVFLL | HLA-DPA1*02:01/DPB1*05:01 | 18-32 | LLFLAFVVFLLVTLA | 57 |
|  | FLAFVVFLL | HLA-DPA1*02:01/DPB1*05:01 | 15-29 | NSVLLFLAFVVFLLV | 58 |
|  | FLAFVVFLL | HLA-DRB1*07:01 | 17-31 | VLLFLAFVVFLLVTL | 59 |
|  | FLAFVVFLL | HLA-DRB1*07:01 | 15-29 | NSVLLFLAFVVFLLV | 62 |
|  | FLAFVVFLL | HLA-DRB1*07:01 | 18-32 | LLFLAFVVFLLVTLA | 62 |
|  | FLAFVVFLL | HLA-DRB1*07:01 | 16-30 | SVLLFLAFVVFLLVT | 65 |
|  | FLAFVVFLL | HLA-DRB1*15:01 | 18-32 | LLFLAFVVFLLVTLA | 69 |
|  | FLAFVVFLL | HLA-DPA1*02:01/DPB1*05:01 | 19-33 | LFLAFVVFLLVTLAI | 97 |
|  | FLAFVVFLL | HLA-DRB1*04:01 | 18-32 | LLFLAFVVFLLVTLA | 98 |
|  | FLAFVVFLL | HLA-DRB1*04:01 | 17-31 | VLLFLAFVVFLLVTL | 99 |
|  | FLAFVVFLL | HLA-DRB1*04:01 | 16-30 | SVLLFLAFVVFLLVT | 118 |
|  | FLAFVVFLL | HLA-DRB1*04:01 | 15-29 | NSVLLFLAFVVFLLV | 119 |
|  | FLAFVVFLL | HLA-DPA1*02:01/DPB1*05:01 | 20-34 | FLAFVVFLLVTLAIL | 139 |
|  | FLAFVVFLL | HLA-DRB5*01:01 | 15-29 | NSVLLFLAFVVFLLV | 160 |
|  | FLAFVVFLL | HLA-DRB5*01:01 | 17-31 | VLLFLAFVVFLLVTL | 197 |
|  | FLAFVVFLL | HLA-DRB1*09:01 | 18-32 | LLFLAFVVFLLVTLA | 198 |
|  | FLAFVVFLL | HLA-DRB1*09:01 | 17-31 | VLLFLAFVVFLLVTL | 207 |
|  | FLAFVVFLL | HLA-DRB5*01:01 | 16-30 | SVLLFLAFVVFLLVT | 226 |
|  | FLAFVVFLL | HLA-DRB5*01:01 | 18-32 | LLFLAFVVFLLVTLA | 237 |
|  | FLAFVVFLL | HLA-DRB1*09:01 | 15-29 | NSVLLFLAFVVFLLV | 238 |
|  | FLAFVVFLL | HLA-DRB1*09:01 | 16-30 | SVLLFLAFVVFLLVT | 252 |
|  | FLAFVVFLL | HLA-DRB1*12:01 | 18-32 | LLFLAFVVFLLVTLA | 303 |
|  | FLAFVVFLL | HLA-DRB1*11:01 | 17-31 | VLLFLAFVVFLLVTL | 303 |
|  | FLAFVVFLL | HLA-DQA1*05:01/DQB1*02:01 | 18-32 | LLFLAFVVFLLVTLA | 362 |
|  | FLAFVVFLL | HLA-DRB1*11:01 | 15-29 | NSVLLFLAFVVFLLV | 421 |
|  | FLAFVVFLL | HLA-DQA1*05:01/DQB1*03:01 | 15-29 | NSVLLFLAFVVFLLV | 454 |
|  | FLAFVVFLL | HLA-DRB1*11:01 | 16-30 | SVLLFLAFVVFLLVT | 502 |
|  | FLAFVVFLL | HLA-DQA1*05:01/DQB1*03:01 | 16-30 | SVLLFLAFVVFLLVT | 511 |
|  | FLAFVVFLL | HLA-DQA1*05:01/DQB1*03:01 | 17-31 | VLLFLAFVVFLLVTL | 513 |
|  | FLAFVVFLL | HLA-DQA1*01:01/DQB1*05:01 | 17-31 | VLLFLAFVVFLLVTL | 546 |
|  | FLAFVVFLL | HLA-DQA1*01:01/DQB1*05:01 | 18-32 | LLFLAFVVFLLVTLA | 556 |
|  | FLAFVVFLL | HLA-DQA1*01:01/DQB1*05:01 | 15-29 | NSVLLFLAFVVFLLV | 652 |
|  | FLAFVVFLL | HLA-DQA1*05:01/DQB1*03:01 | 18-32 | LLFLAFVVFLLVTLA | 666 |
|  | FLAFVVFLL | HLA-DQA1*01:01/DQB1*05:01 | 16-30 | SVLLFLAFVVFLLVT | 731 |
|  | FLAFVVFLL | HLA-DQA1*05:01/DQB1*03:01 | 19-33 | LFLAFVVFLLVTLAI | 901 |
|  | FLAFVVFLL | HLA-DRB3*01:01 | 17-31 | VLLFLAFVVFLLVTL | 1059 |
|  | FLAFVVFLL | HLA-DRB3*01:01 | 15-29 | NSVLLFLAFVVFLLV | 1070 |
|  | FLAFVVFLL | HLA-DRB3*01:01 | 16-30 | SVLLFLAFVVFLLVT | 1071 |
|  | FLAFVVFLL | HLA-DRB3*01:01 | 18-32 | LLFLAFVVFLLVTLA | 1242 |
|  | FLAFVVFLL | HLA-DRB1*03:01 | 18-32 | LLFLAFVVFLLVTLA | 1257 |
|  | FLAFVVFLL | HLA-DQA1*04:01/DQB1*04:02 | 18-32 | LLFLAFVVFLLVTLA | 1599 |
|  | FLAFVVFLL | HLA-DRB1*08:02 | 16-30 | SVLLFLAFVVFLLVT | 1929 |
|  | FLAFVVFLL | HLA-DRB3*01:01 | 20-34 | FLAFVVFLLVTLAIL | 1935 |
|  | FLAFVVFLL | HLA-DRB1*08:02 | 15-29 | NSVLLFLAFVVFLLV | 1971 |
|  | FLLVTLAIL | HLA-DRB1*01:01 | 23-37 | FVVFLLVTLAILTAL | 12 |
|  | FLLVTLAIL | HLA-DRB1*01:01 | 22-36 | AFVVFLLVTLAILTA | 13 |
|  | FLLVTLAIL | HLA-DRB1*01:01 | 24-38 | VVFLLVTLAILTALR | 13 |
|  | FLLVTLAIL | HLA-DRB1*01:01 | 21-35 | LAFVVFLLVTLAILT | 15 |
|  | FLLVTLAIL | HLA-DPA1*03:01/DPB1*04:02 | 23-37 | FVVFLLVTLAILTAL | 18 |
|  | FLLVTLAIL | HLA-DPA1*03:01/DPB1*04:02 | 22-36 | AFVVFLLVTLAILTA | 19 |
|  | FLLVTLAIL | HLA-DPA1*03:01/DPB1*04:02 | 24-38 | VVFLLVTLAILTALR | 20 |
|  | FLLVTLAIL | HLA-DPA1*02:01/DPB1*01:01 | 23-37 | FVVFLLVTLAILTAL | 41 |
|  | FLLVTLAIL | HLA-DPA1*02:01/DPB1*01:01 | 22-36 | AFVVFLLVTLAILTA | 43 |
|  | FLLVTLAIL | HLA-DPA1*03:01/DPB1*04:02 | 25-39 | VFLLVTLAILTALRL | 44 |
|  | FLLVTLAIL | HLA-DRB1*07:01 | 21-35 | LAFVVFLLVTLAILT | 48 |
|  | FLLVTLAIL | HLA-DPA1*03:01/DPB1*04:02 | 26-40 | FLLVTLAILTALRLC | 49 |
|  | FLLVTLAIL | HLA-DPA1*02:01/DPB1*01:01 | 24-38 | VVFLLVTLAILTALR | 52 |
|  | FLLVTLAIL | HLA-DPA1*01:03/DPB1*02:01 | 23-37 | FVVFLLVTLAILTAL | 55 |
|  | FLLVTLAIL | HLA-DPA1*01:03/DPB1*02:01 | 22-36 | AFVVFLLVTLAILTA | 56 |
|  | FLLVTLAIL | HLA-DPA1*01:03/DPB1*02:01 | 24-38 | VVFLLVTLAILTALR | 63 |
|  | FLLVTLAIL | HLA-DRB1*04:05 | 22-36 | AFVVFLLVTLAILTA | 76 |
|  | FLLVTLAIL | HLA-DRB1*04:05 | 23-37 | FVVFLLVTLAILTAL | 78 |
|  | FLLVTLAIL | HLA-DPA1*02:01/DPB1*01:01 | 25-39 | VFLLVTLAILTALRL | 82 |
|  | FLLVTLAIL | HLA-DRB1*04:05 | 24-38 | VVFLLVTLAILTALR | 90 |
|  | FLLVTLAIL | HLA-DPA1*02:01/DPB1*01:01 | 26-40 | FLLVTLAILTALRLC | 102 |
|  | FLLVTLAIL | HLA-DPA1*01:03/DPB1*02:01 | 25-39 | VFLLVTLAILTALRL | 114 |
|  | FLLVTLAIL | HLA-DPA1*01:03/DPB1*02:01 | 26-40 | FLLVTLAILTALRLC | 124 |
|  | FLLVTLAIL | HLA-DRB4*01:01 | 22-36 | AFVVFLLVTLAILTA | 125 |
|  | FLLVTLAIL | HLA-DPA1*01/DPB1*04:01 | 23-37 | FVVFLLVTLAILTAL | 155 |
|  | FLLVTLAIL | HLA-DPA1*01/DPB1*04:01 | 22-36 | AFVVFLLVTLAILTA | 158 |
|  | FLLVTLAIL | HLA-DRB4*01:01 | 21-35 | LAFVVFLLVTLAILT | 161 |
|  | FLLVTLAIL | HLA-DPA1*01/DPB1*04:01 | 24-38 | VVFLLVTLAILTALR | 173 |
|  | FLLVTLAIL | HLA-DRB5*01:01 | 24-38 | VVFLLVTLAILTALR | 218 |
|  | FLLVTLAIL | HLA-DRB5*01:01 | 21-35 | LAFVVFLLVTLAILT | 235 |
|  | FLLVTLAIL | HLA-DRB5*01:01 | 23-37 | FVVFLLVTLAILTAL | 264 |
|  | FLLVTLAIL | HLA-DRB5*01:01 | 22-36 | AFVVFLLVTLAILTA | 273 |
|  | FLLVTLAIL | HLA-DPA1*01/DPB1*04:01 | 25-39 | VFLLVTLAILTALRL | 322 |
|  | FLLVTLAIL | HLA-DRB1*09:01 | 22-36 | AFVVFLLVTLAILTA | 326 |
|  | FLLVTLAIL | HLA-DPA1*01/DPB1*04:01 | 26-40 | FLLVTLAILTALRLC | 344 |
|  | FLLVTLAIL | HLA-DPA1*02:01/DPB1*05:01 | 23-37 | FVVFLLVTLAILTAL | 456 |
|  | FLLVTLAIL | HLA-DPA1*02:01/DPB1*05:01 | 22-36 | AFVVFLLVTLAILTA | 485 |
|  | FLLVTLAIL | HLA-DPA1*02:01/DPB1*05:01 | 24-38 | VVFLLVTLAILTALR | 518 |
|  | FLLVTLAIL | HLA-DQA1*05:01/DQB1*03:01 | 21-35 | LAFVVFLLVTLAILT | 632 |
|  | FLLVTLAIL | HLA-DPA1*02:01/DPB1*05:01 | 25-39 | VFLLVTLAILTALRL | 690 |
|  | FLLVTLAIL | HLA-DQA1*05:01/DQB1*02:01 | 23-37 | FVVFLLVTLAILTAL | 754 |
|  | FLLVTLAIL | HLA-DQA1*05:01/DQB1*02:01 | 22-36 | AFVVFLLVTLAILTA | 759 |
|  | FLLVTLAIL | HLA-DQA1*05:01/DQB1*02:01 | 24-38 | VVFLLVTLAILTALR | 916 |
|  | FLLVTLAIL | HLA-DQA1*01:01/DQB1*05:01 | 23-37 | FVVFLLVTLAILTAL | 1539 |
|  | FLLVTLAIL | HLA-DQA1*04:01/DQB1*04:02 | 22-36 | AFVVFLLVTLAILTA | 1552 |
|  | FLLVTLAIL | HLA-DQA1*01:01/DQB1*05:01 | 22-36 | AFVVFLLVTLAILTA | 1554 |
|  | FLLVTLAIL | HLA-DQA1*03:01/DQB1*03:02 | 23-37 | FVVFLLVTLAILTAL | 1868 |
|  | FLLVTLAIL | HLA-DQA1*03:01/DQB1*03:02 | 22-36 | AFVVFLLVTLAILTA | 1957 |
|  | FLLVTLAIL | HLA-DRB3*01:01 | 21-35 | LAFVVFLLVTLAILT | 2044 |
|  | FLLVTLAIL | HLA-DQA1*01:01/DQB1*05:01 | 24-38 | VVFLLVTLAILTALR | 2134 |
|  | FLLVTLAIL | HLA-DQA1*03:01/DQB1*03:02 | 21-35 | LAFVVFLLVTLAILT | 2147 |
|  | FLLVTLAIL | HLA-DRB3*01:01 | 23-37 | FVVFLLVTLAILTAL | 2158 |
|  | FLLVTLAIL | HLA-DRB3*01:01 | 22-36 | AFVVFLLVTLAILTA | 2213 |
|  | FLLVTLAIL | HLA-DRB3*01:01 | 24-38 | VVFLLVTLAILTALR | 2213 |
|  | LLFLAFVVF | HLA-DPA1*01:03/DPB1*02:01 | 14-28 | VNSVLLFLAFVVFLL | 38 |
|  | LLFLAFVVF | HLA-DPA1*01:03/DPB1*02:01 | 13-27 | IVNSVLLFLAFVVFL | 57 |
|  | LLFLAFVVF | HLA-DPA1*02:01/DPB1*05:01 | 14-28 | VNSVLLFLAFVVFLL | 94 |
|  | LLFLAFVVF | HLA-DRB1*04:01 | 14-28 | VNSVLLFLAFVVFLL | 127 |
|  | LLFLAFVVF | HLA-DPA1*01/DPB1*04:01 | 14-28 | VNSVLLFLAFVVFLL | 201 |
|  | LLFLAFVVF | HLA-DRB4*01:01 | 15-29 | NSVLLFLAFVVFLLV | 256 |
|  | LLFLAFVVF | HLA-DPA1*02:01/DPB1*05:01 | 13-27 | IVNSVLLFLAFVVFL | 273 |
|  | LLFLAFVVF | HLA-DPA1*01/DPB1*04:01 | 13-27 | IVNSVLLFLAFVVFL | 302 |
|  | LLFLAFVVF | HLA-DRB4*01:01 | 13-27 | IVNSVLLFLAFVVFL | 942 |
|  | LLFLAFVVF | HLA-DRB4*01:01 | 14-28 | VNSVLLFLAFVVFLL | 1077 |
|  | LLFLAFVVF | HLA-DQA1*03:01/DQB1*03:02 | 16-30 | SVLLFLAFVVFLLVT | 1286 |
|  | LTALRLCAY | HLA-DQA1*01:02/DQB1*06:02 | 29-43 | VTLAILTALRLCAYC | 122 |
|  | LTALRLCAY | HLA-DQA1*01:02/DQB1*06:02 | 30-44 | TLAILTALRLCAYCC | 128 |
|  | LTALRLCAY | HLA-DQA1*01:02/DQB1*06:02 | 31-45 | LAILTALRLCAYCCN | 129 |
|  | LTALRLCAY | HLA-DQA1*01:02/DQB1*06:02 | 32-46 | AILTALRLCAYCCNI | 148 |
|  | LTALRLCAY | HLA-DQA1*01:02/DQB1*06:02 | 33-47 | ILTALRLCAYCCNIV | 385 |
|  | LTALRLCAY | HLA-DRB1*01:01 | 32-46 | AILTALRLCAYCCNI | 405 |
|  | LTALRLCAY | HLA-DQA1*01:02/DQB1*06:02 | 34-48 | LTALRLCAYCCNIVN | 476 |
|  | LTALRLCAY | HLA-DRB1*12:01 | 30-44 | TLAILTALRLCAYCC | 603 |
|  | LTALRLCAY | HLA-DRB1*12:01 | 31-45 | LAILTALRLCAYCCN | 729 |
|  | LTALRLCAY | HLA-DRB1*12:01 | 32-46 | AILTALRLCAYCCNI | 1332 |
|  | LTALRLCAY | HLA-DPA1*03:01/DPB1*04:02 | 32-46 | AILTALRLCAYCCNI | 1726 |
|  | LTALRLCAY | HLA-DPA1*01:03/DPB1*02:01 | 32-46 | AILTALRLCAYCCNI | 1731 |
|  | SEETGTLIV | HLA-DRB1*03:01 | 6-20 | SEETGTLIVNSVLLF | 98 |
|  | SEETGTLIV | HLA-DRB3*01:01 | 6-20 | SEETGTLIVNSVLLF | 156 |
|  | SEETGTLIV | HLA-DRB1*07:01 | 4-18 | FVSEETGTLIVNSVL | 452 |
|  | SEETGTLIV | HLA-DQA1*01:02/DQB1*06:02 | 1-15 | MYSFVSEETGTLIVN | 2453 |
|  | VFLLVTLAI | HLA-DRB1*01:01 | 20-34 | FLAFVVFLLVTLAIL | 15 |
|  | VFLLVTLAI | HLA-DRB1*07:01 | 20-34 | FLAFVVFLLVTLAIL | 79 |
|  | VFLLVTLAI | HLA-DRB1*04:01 | 22-36 | AFVVFLLVTLAILTA | 93 |
|  | VFLLVTLAI | HLA-DRB1*04:01 | 20-34 | FLAFVVFLLVTLAIL | 107 |
|  | VFLLVTLAI | HLA-DRB1*04:01 | 21-35 | LAFVVFLLVTLAILT | 111 |
|  | VFLLVTLAI | HLA-DQA1*01:02/DQB1*06:02 | 22-36 | AFVVFLLVTLAILTA | 250 |
|  | VFLLVTLAI | HLA-DRB1*13:02 | 22-36 | AFVVFLLVTLAILTA | 439 |
|  | VFLLVTLAI | HLA-DRB1*13:02 | 23-37 | FVVFLLVTLAILTAL | 442 |
|  | VFLLVTLAI | HLA-DRB1*13:02 | 21-35 | LAFVVFLLVTLAILT | 443 |
|  | VFLLVTLAI | HLA-DRB1*13:02 | 20-34 | FLAFVVFLLVTLAIL | 466 |
|  | VFLLVTLAI | HLA-DRB1*13:02 | 25-39 | VFLLVTLAILTALRL | 661 |
|  | VFLLVTLAI | HLA-DRB1*13:02 | 24-38 | VVFLLVTLAILTALR | 937 |
|  | VFLLVTLAI | HLA-DQA1*04:01/DQB1*04:02 | 20-34 | FLAFVVFLLVTLAIL | 1946 |
|  | VFLLVTLAI | HLA-DQA1*04:01/DQB1*04:02 | 21-35 | LAFVVFLLVTLAILT | 2020 |
|  | VFLLVTLAI | HLA-DRB1*03:01 | 20-34 | FLAFVVFLLVTLAIL | 2754 |
| **Membrane glycoprotein** | | | | | |
| **No** | **Core sequence** | **MHC II alleles** | **position** | **Peptide** | **IC50 value** |
|  | AYANRNRFL | HLA-DRB1*13:02 | 35-49 | LQFAYANRNRFLYII | 473 |
|  | AYANRNRFL | HLA-DRB1*13:02 | 34-48 | LLQFAYANRNRFLYI | 484 |
|  | AYANRNRFL | HLA-DRB1*01:01 | 36-50 | QFAYANRNRFLYIIK | 573 |
|  | AYANRNRFL | HLA-DRB1*13:02 | 36-50 | QFAYANRNRFLYIIK | 651 |
|  | AYANRNRFL | HLA-DPA1*02:01/DPB1*05:01 | 33-47 | CLLQFAYANRNRFLY | 895 |
|  | AYANRNRFL | HLA-DRB1*15:01 | 36-50 | QFAYANRNRFLYIIK | 1475 |
|  | FAYANRNRF | HLA-DRB5*01:01 | 32-46 | ICLLQFAYANRNRFL | 42 |
|  | FAYANRNRF | HLA-DRB5*01:01 | 33-47 | CLLQFAYANRNRFLY | 43 |
|  | FAYANRNRF | HLA-DRB5*01:01 | 34-48 | LLQFAYANRNRFLYI | 51 |
|  | FAYANRNRF | HLA-DRB5*01:01 | 35-49 | LQFAYANRNRFLYII | 55 |
|  | FAYANRNRF | HLA-DRB1*11:01 | 34-48 | LLQFAYANRNRFLYI | 89 |
|  | FAYANRNRF | HLA-DRB1*11:01 | 33-47 | CLLQFAYANRNRFLY | 93 |
|  | FAYANRNRF | HLA-DRB1*11:01 | 35-49 | LQFAYANRNRFLYII | 93 |
|  | FAYANRNRF | HLA-DRB1*11:01 | 32-46 | ICLLQFAYANRNRFL | 96 |
|  | FAYANRNRF | HLA-DRB1*07:01 | 32-46 | ICLLQFAYANRNRFL | 141 |
|  | FAYANRNRF | HLA-DRB1*07:01 | 33-47 | CLLQFAYANRNRFLY | 142 |
|  | FAYANRNRF | HLA-DRB1*07:01 | 34-48 | LLQFAYANRNRFLYI | 173 |
|  | FAYANRNRF | HLA-DPA1*01:03/DPB1*02:01 | 34-48 | LLQFAYANRNRFLYI | 185 |
|  | FAYANRNRF | HLA-DRB5*01:01 | 36-50 | QFAYANRNRFLYIIK | 186 |
|  | FAYANRNRF | HLA-DRB5*01:01 | 37-51 | FAYANRNRFLYIIKL | 186 |
|  | FAYANRNRF | HLA-DRB1*07:01 | 35-49 | LQFAYANRNRFLYII | 186 |
|  | FAYANRNRF | HLA-DRB1*01:01 | 34-48 | LLQFAYANRNRFLYI | 210 |
|  | FAYANRNRF | HLA-DRB1*01:01 | 35-49 | LQFAYANRNRFLYII | 239 |
|  | FAYANRNRF | HLA-DRB1*11:01 | 36-50 | QFAYANRNRFLYIIK | 247 |
|  | FAYANRNRF | HLA-DRB1*11:01 | 37-51 | FAYANRNRFLYIIKL | 255 |
|  | FAYANRNRF | HLA-DPA1*01/DPB1*04:01 | 34-48 | LLQFAYANRNRFLYI | 365 |
|  | FAYANRNRF | HLA-DRB1*01:01 | 37-51 | FAYANRNRFLYIIKL | 412 |
|  | FAYANRNRF | HLA-DRB1*03:01 | 34-48 | LLQFAYANRNRFLYI | 451 |
|  | FAYANRNRF | HLA-DRB1*03:01 | 33-47 | CLLQFAYANRNRFLY | 473 |
|  | FAYANRNRF | HLA-DRB1*03:01 | 35-49 | LQFAYANRNRFLYII | 475 |
|  | FAYANRNRF | HLA-DRB1*03:01 | 32-46 | ICLLQFAYANRNRFL | 480 |
|  | FAYANRNRF | HLA-DRB1*04:01 | 34-48 | LLQFAYANRNRFLYI | 511 |
|  | FAYANRNRF | HLA-DRB1*07:01 | 36-50 | QFAYANRNRFLYIIK | 556 |
|  | FAYANRNRF | HLA-DPA1*01:03/DPB1*02:01 | 32-46 | ICLLQFAYANRNRFL | 571 |
|  | FAYANRNRF | HLA-DRB1*07:01 | 37-51 | FAYANRNRFLYIIKL | 607 |
|  | FAYANRNRF | HLA-DRB1*04:01 | 35-49 | LQFAYANRNRFLYII | 676 |
|  | FAYANRNRF | HLA-DRB1*09:01 | 34-48 | LLQFAYANRNRFLYI | 714 |
|  | FAYANRNRF | HLA-DRB1*09:01 | 35-49 | LQFAYANRNRFLYII | 765 |
|  | FAYANRNRF | HLA-DPA1*01:03/DPB1*02:01 | 33-47 | CLLQFAYANRNRFLY | 809 |
|  | FAYANRNRF | HLA-DRB3*01:01 | 34-48 | LLQFAYANRNRFLYI | 832 |
|  | FAYANRNRF | HLA-DRB3*01:01 | 35-49 | LQFAYANRNRFLYII | 854 |
|  | FAYANRNRF | HLA-DRB1*04:05 | 34-48 | LLQFAYANRNRFLYI | 855 |
|  | FAYANRNRF | HLA-DRB3*01:01 | 33-47 | CLLQFAYANRNRFLY | 868 |
|  | FAYANRNRF | HLA-DRB3*01:01 | 32-46 | ICLLQFAYANRNRFL | 883 |
|  | FAYANRNRF | HLA-DPA1*03:01/DPB1*04:02 | 34-48 | LLQFAYANRNRFLYI | 939 |
|  | FAYANRNRF | HLA-DRB1*04:05 | 35-49 | LQFAYANRNRFLYII | 946 |
|  | FAYANRNRF | HLA-DRB1*04:05 | 36-50 | QFAYANRNRFLYIIK | 1323 |
|  | FAYANRNRF | HLA-DRB1*03:01 | 36-50 | QFAYANRNRFLYIIK | 1400 |
|  | FAYANRNRF | HLA-DRB1*03:01 | 37-51 | FAYANRNRFLYIIKL | 1487 |
|  | FAYANRNRF | HLA-DPA1*01/DPB1*04:01 | 32-46 | ICLLQFAYANRNRFL | 1814 |
|  | FAYANRNRF | HLA-DRB1*04:01 | 36-50 | QFAYANRNRFLYIIK | 1829 |
|  | FAYANRNRF | HLA-DPA1*03:01/DPB1*04:02 | 32-46 | ICLLQFAYANRNRFL | 1889 |
|  | FAYANRNRF | HLA-DPA1*01/DPB1*04:01 | 33-47 | CLLQFAYANRNRFLY | 1956 |
|  | FAYANRNRF | HLA-DRB3*01:01 | 36-50 | QFAYANRNRFLYIIK | 1965 |
|  | FAYANRNRF | HLA-DRB1*09:01 | 37-51 | FAYANRNRFLYIIKL | 2068 |
|  | FAYANRNRF | HLA-DRB3*01:01 | 37-51 | FAYANRNRFLYIIKL | 2184 |
|  | FAYANRNRF | HLA-DRB1*04:01 | 37-51 | FAYANRNRFLYIIKL | 2225 |
|  | FAYANRNRF | HLA-DRB1*09:01 | 36-50 | QFAYANRNRFLYIIK | 2394 |
|  | FAYANRNRF | HLA-DPA1*03:01/DPB1*04:02 | 33-47 | CLLQFAYANRNRFLY | 2553 |
|  | FLYIIKLIF | HLA-DRB1*11:01 | 42-56 | RNRFLYIIKLIFLWL | 10 |
|  | FLYIIKLIF | HLA-DRB1*11:01 | 40-54 | ANRNRFLYIIKLIFL | 11 |
|  | FLYIIKLIF | HLA-DRB1*11:01 | 41-55 | NRNRFLYIIKLIFLW | 11 |
|  | FLYIIKLIF | HLA-DRB1*11:01 | 43-57 | NRFLYIIKLIFLWLL | 11 |
|  | FLYIIKLIF | HLA-DRB1*11:01 | 44-58 | RFLYIIKLIFLWLLW | 30 |
|  | FLYIIKLIF | HLA-DRB5*01:01 | 42-56 | RNRFLYIIKLIFLWL | 32 |
|  | FLYIIKLIF | HLA-DRB1*11:01 | 45-59 | FLYIIKLIFLWLLWP | 32 |
|  | FLYIIKLIF | HLA-DRB5*01:01 | 43-57 | NRFLYIIKLIFLWLL | 33 |
|  | FLYIIKLIF | HLA-DRB1*07:01 | 42-56 | RNRFLYIIKLIFLWL | 33 |
|  | FLYIIKLIF | HLA-DRB1*07:01 | 43-57 | NRFLYIIKLIFLWLL | 33 |
|  | FLYIIKLIF | HLA-DRB5*01:01 | 41-55 | NRNRFLYIIKLIFLW | 34 |
|  | FLYIIKLIF | HLA-DRB1*07:01 | 40-54 | ANRNRFLYIIKLIFL | 35 |
|  | FLYIIKLIF | HLA-DRB1*07:01 | 41-55 | NRNRFLYIIKLIFLW | 36 |
|  | FLYIIKLIF | HLA-DRB5*01:01 | 40-54 | ANRNRFLYIIKLIFL | 37 |
|  | FLYIIKLIF | HLA-DRB1*04:05 | 43-57 | NRFLYIIKLIFLWLL | 51 |
|  | FLYIIKLIF | HLA-DRB1*04:05 | 42-56 | RNRFLYIIKLIFLWL | 52 |
|  | FLYIIKLIF | HLA-DRB1*04:05 | 40-54 | ANRNRFLYIIKLIFL | 54 |
|  | FLYIIKLIF | HLA-DPA1*02:01/DPB1*01:01 | 40-54 | ANRNRFLYIIKLIFL | 54 |
|  | FLYIIKLIF | HLA-DRB1*04:05 | 41-55 | NRNRFLYIIKLIFLW | 55 |
|  | FLYIIKLIF | HLA-DRB1*01:01 | 40-54 | ANRNRFLYIIKLIFL | 62 |
|  | FLYIIKLIF | HLA-DPA1*03:01/DPB1*04:02 | 40-54 | ANRNRFLYIIKLIFL | 64 |
|  | FLYIIKLIF | HLA-DRB1*15:01 | 40-54 | ANRNRFLYIIKLIFL | 68 |
|  | FLYIIKLIF | HLA-DRB5*01:01 | 44-58 | RFLYIIKLIFLWLLW | 76 |
|  | FLYIIKLIF | HLA-DRB1*07:01 | 44-58 | RFLYIIKLIFLWLLW | 76 |
|  | FLYIIKLIF | HLA-DRB5*01:01 | 45-59 | FLYIIKLIFLWLLWP | 80 |
|  | FLYIIKLIF | HLA-DRB1*04:04 | 43-57 | NRFLYIIKLIFLWLL | 84 |
|  | FLYIIKLIF | HLA-DPA1*01:03/DPB1*02:01 | 42-56 | RNRFLYIIKLIFLWL | 86 |
|  | FLYIIKLIF | HLA-DRB1*07:01 | 45-59 | FLYIIKLIFLWLLWP | 87 |
|  | FLYIIKLIF | HLA-DRB1*04:04 | 45-59 | FLYIIKLIFLWLLWP | 94 |
|  | FLYIIKLIF | HLA-DRB1*04:04 | 42-56 | RNRFLYIIKLIFLWL | 101 |
|  | FLYIIKLIF | HLA-DRB1*04:04 | 41-55 | NRNRFLYIIKLIFLW | 110 |
|  | FLYIIKLIF | HLA-DRB1*04:05 | 44-58 | RFLYIIKLIFLWLLW | 114 |
|  | FLYIIKLIF | HLA-DRB4*01:01 | 40-54 | ANRNRFLYIIKLIFL | 117 |
|  | FLYIIKLIF | HLA-DRB1*04:04 | 40-54 | ANRNRFLYIIKLIFL | 121 |
|  | FLYIIKLIF | HLA-DRB1*04:05 | 45-59 | FLYIIKLIFLWLLWP | 126 |
|  | FLYIIKLIF | HLA-DRB1*12:01 | 42-56 | RNRFLYIIKLIFLWL | 146 |
|  | FLYIIKLIF | HLA-DRB1*04:04 | 44-58 | RFLYIIKLIFLWLLW | 153 |
|  | FLYIIKLIF | HLA-DPA1*01:03/DPB1*02:01 | 41-55 | NRNRFLYIIKLIFLW | 154 |
|  | FLYIIKLIF | HLA-DPA1*01:03/DPB1*02:01 | 40-54 | ANRNRFLYIIKLIFL | 158 |
|  | FLYIIKLIF | HLA-DRB1*12:01 | 41-55 | NRNRFLYIIKLIFLW | 199 |
|  | FLYIIKLIF | HLA-DPA1*02:01/DPB1*05:01 | 40-54 | ANRNRFLYIIKLIFL | 208 |
|  | FLYIIKLIF | HLA-DRB1*12:01 | 40-54 | ANRNRFLYIIKLIFL | 333 |
|  | FLYIIKLIF | HLA-DPA1*01/DPB1*04:01 | 40-54 | ANRNRFLYIIKLIFL | 346 |
|  | FLYIIKLIF | HLA-DRB1*04:01 | 43-57 | NRFLYIIKLIFLWLL | 425 |
|  | FLYIIKLIF | HLA-DRB1*04:01 | 42-56 | RNRFLYIIKLIFLWL | 439 |
|  | FLYIIKLIF | HLA-DRB1*04:01 | 41-55 | NRNRFLYIIKLIFLW | 460 |
|  | FLYIIKLIF | HLA-DRB1*04:01 | 40-54 | ANRNRFLYIIKLIFL | 471 |
|  | FLYIIKLIF | HLA-DRB1*03:01 | 42-56 | RNRFLYIIKLIFLWL | 508 |
|  | FLYIIKLIF | HLA-DRB1*03:01 | 43-57 | NRFLYIIKLIFLWLL | 521 |
|  | FLYIIKLIF | HLA-DRB1*03:01 | 40-54 | ANRNRFLYIIKLIFL | 527 |
|  | FLYIIKLIF | HLA-DRB1*03:01 | 41-55 | NRNRFLYIIKLIFLW | 540 |
|  | FLYIIKLIF | HLA-DRB3*01:01 | 43-57 | NRFLYIIKLIFLWLL | 635 |
|  | FLYIIKLIF | HLA-DRB3*01:01 | 42-56 | RNRFLYIIKLIFLWL | 646 |
|  | FLYIIKLIF | HLA-DRB3*01:01 | 41-55 | NRNRFLYIIKLIFLW | 663 |
|  | FLYIIKLIF | HLA-DRB1*04:01 | 44-58 | RFLYIIKLIFLWLLW | 668 |
|  | FLYIIKLIF | HLA-DQA1*05:01/DQB1*02:01 | 43-57 | NRFLYIIKLIFLWLL | 700 |
|  | FLYIIKLIF | HLA-DQA1*05:01/DQB1*02:01 | 42-56 | RNRFLYIIKLIFLWL | 715 |
|  | FLYIIKLIF | HLA-DRB1*04:01 | 45-59 | FLYIIKLIFLWLLWP | 761 |
|  | FLYIIKLIF | HLA-DRB1*09:01 | 42-56 | RNRFLYIIKLIFLWL | 800 |
|  | FLYIIKLIF | HLA-DQA1*05:01/DQB1*02:01 | 41-55 | NRNRFLYIIKLIFLW | 817 |
|  | FLYIIKLIF | HLA-DRB1*08:02 | 40-54 | ANRNRFLYIIKLIFL | 862 |
|  | FLYIIKLIF | HLA-DRB3*01:01 | 40-54 | ANRNRFLYIIKLIFL | 872 |
|  | FLYIIKLIF | HLA-DQA1*05:01/DQB1*02:01 | 40-54 | ANRNRFLYIIKLIFL | 1020 |
|  | FLYIIKLIF | HLA-DRB1*09:01 | 40-54 | ANRNRFLYIIKLIFL | 1063 |
|  | FLYIIKLIF | HLA-DRB1*09:01 | 41-55 | NRNRFLYIIKLIFLW | 1086 |
|  | FLYIIKLIF | HLA-DRB1*13:02 | 43-57 | NRFLYIIKLIFLWLL | 1237 |
|  | FLYIIKLIF | HLA-DRB1*03:01 | 44-58 | RFLYIIKLIFLWLLW | 1423 |
|  | FLYIIKLIF | HLA-DRB1*03:01 | 45-59 | FLYIIKLIFLWLLWP | 1464 |
|  | FLYIIKLIF | HLA-DRB1*13:02 | 42-56 | RNRFLYIIKLIFLWL | 1726 |
|  | FLYIIKLIF | HLA-DRB1*13:02 | 40-54 | ANRNRFLYIIKLIFL | 1861 |
|  | FLYIIKLIF | HLA-DRB1*13:02 | 41-55 | NRNRFLYIIKLIFLW | 2195 |
|  | GLMWLSYFI | HLA-DRB1*15:01 | 87-101 | LVGLMWLSYFIASFR | 113 |
|  | GLMWLSYFI | HLA-DRB1*15:01 | 86-100 | CLVGLMWLSYFIASF | 132 |
|  | GLMWLSYFI | HLA-DRB1*15:01 | 85-99 | ACLVGLMWLSYFIAS | 137 |
|  | GLMWLSYFI | HLA-DRB1*15:01 | 84-98 | MACLVGLMWLSYFIA | 140 |
|  | GLMWLSYFI | HLA-DQA1*05:01/DQB1*02:01 | 86-100 | CLVGLMWLSYFIASF | 1062 |
|  | GLMWLSYFI | HLA-DRB1*07:01 | 86-100 | CLVGLMWLSYFIASF | 1155 |
|  | GLMWLSYFI | HLA-DRB1*07:01 | 84-98 | MACLVGLMWLSYFIA | 1744 |
|  | GLMWLSYFI | HLA-DQA1*01:01/DQB1*05:01 | 85-99 | ACLVGLMWLSYFIAS | 1766 |
|  | GLMWLSYFI | HLA-DQA1*01:01/DQB1*05:01 | 84-98 | MACLVGLMWLSYFIA | 2150 |
|  | LAAVYRINW | HLA-DRB1*11:01 | 62-76 | LACFVLAAVYRINWI | 166 |
|  | LAAVYRINW | HLA-DRB1*11:01 | 63-77 | ACFVLAAVYRINWIT | 168 |
|  | LAAVYRINW | HLA-DRB1*11:01 | 65-79 | FVLAAVYRINWITGG | 170 |
|  | LAAVYRINW | HLA-DRB1*11:01 | 64-78 | CFVLAAVYRINWITG | 188 |
|  | LAAVYRINW | HLA-DRB1*11:01 | 66-80 | VLAAVYRINWITGGI | 366 |
|  | LAAVYRINW | HLA-DRB1*11:01 | 67-81 | LAAVYRINWITGGIA | 368 |
|  | LAAVYRINW | HLA-DRB1*08:02 | 65-79 | FVLAAVYRINWITGG | 1403 |
|  | LAAVYRINW | HLA-DRB1*08:02 | 64-78 | CFVLAAVYRINWITG | 1454 |
|  | LAAVYRINW | HLA-DQA1*01:02/DQB1*06:02 | 65-79 | FVLAAVYRINWITGG | 1574 |
|  | LAAVYRINW | HLA-DQA1*01:02/DQB1*06:02 | 63-77 | ACFVLAAVYRINWIT | 1613 |
|  | LAAVYRINW | HLA-DQA1*01:02/DQB1*06:02 | 64-78 | CFVLAAVYRINWITG | 1778 |
|  | LAAVYRINW | HLA-DRB1*08:02 | 62-76 | LACFVLAAVYRINWI | 1950 |
|  | LAAVYRINW | HLA-DRB1*08:02 | 63-77 | ACFVLAAVYRINWIT | 2018 |
|  | LSYFIASFR | HLA-DRB5*01:01 | 90-104 | LMWLSYFIASFRLFA | 24 |
|  | LSYFIASFR | HLA-DRB5*01:01 | 91-105 | MWLSYFIASFRLFAR | 25 |
|  | LSYFIASFR | HLA-DRB5*01:01 | 89-103 | GLMWLSYFIASFRLF | 28 |
|  | LSYFIASFR | HLA-DRB5*01:01 | 88-102 | VGLMWLSYFIASFRL | 29 |
|  | LSYFIASFR | HLA-DRB1*04:04 | 90-104 | LMWLSYFIASFRLFA | 32 |
|  | LSYFIASFR | HLA-DRB1*04:04 | 88-102 | VGLMWLSYFIASFRL | 33 |
|  | LSYFIASFR | HLA-DRB1*04:04 | 89-103 | GLMWLSYFIASFRLF | 33 |
|  | LSYFIASFR | HLA-DRB1*04:04 | 91-105 | MWLSYFIASFRLFAR | 34 |
|  | LSYFIASFR | HLA-DRB5*01:01 | 93-107 | LSYFIASFRLFARTR | 60 |
|  | LSYFIASFR | HLA-DRB5*01:01 | 92-106 | WLSYFIASFRLFART | 62 |
|  | LSYFIASFR | HLA-DRB1*04:05 | 90-104 | LMWLSYFIASFRLFA | 67 |
|  | LSYFIASFR | HLA-DRB1*15:01 | 88-102 | VGLMWLSYFIASFRL | 71 |
|  | LSYFIASFR | HLA-DRB1*04:05 | 89-103 | GLMWLSYFIASFRLF | 77 |
|  | LSYFIASFR | HLA-DRB1*04:05 | 88-102 | VGLMWLSYFIASFRL | 78 |
|  | LSYFIASFR | HLA-DRB1*04:05 | 91-105 | MWLSYFIASFRLFAR | 80 |
|  | LSYFIASFR | HLA-DRB1*04:04 | 93-107 | LSYFIASFRLFARTR | 98 |
|  | LSYFIASFR | HLA-DRB1*04:04 | 92-106 | WLSYFIASFRLFART | 99 |
|  | LSYFIASFR | HLA-DRB1*09:01 | 90-104 | LMWLSYFIASFRLFA | 104 |
|  | LSYFIASFR | HLA-DRB1*07:01 | 88-102 | VGLMWLSYFIASFRL | 143 |
|  | LSYFIASFR | HLA-DRB1*12:01 | 90-104 | LMWLSYFIASFRLFA | 235 |
|  | LSYFIASFR | HLA-DRB1*12:01 | 91-105 | MWLSYFIASFRLFAR | 252 |
|  | LSYFIASFR | HLA-DRB1*04:01 | 91-105 | MWLSYFIASFRLFAR | 252 |
|  | LSYFIASFR | HLA-DRB1*12:01 | 89-103 | GLMWLSYFIASFRLF | 254 |
|  | LSYFIASFR | HLA-DRB1*11:01 | 89-103 | GLMWLSYFIASFRLF | 275 |
|  | LSYFIASFR | HLA-DPA1*02:01/DPB1*05:01 | 89-103 | GLMWLSYFIASFRLF | 313 |
|  | LSYFIASFR | HLA-DPA1*02:01/DPB1*05:01 | 88-102 | VGLMWLSYFIASFRL | 362 |
|  | LSYFIASFR | HLA-DRB1*12:01 | 88-102 | VGLMWLSYFIASFRL | 484 |
|  | LSYFIASFR | HLA-DQA1*05:01/DQB1*03:01 | 90-104 | LMWLSYFIASFRLFA | 588 |
|  | LSYFIASFR | HLA-DRB1*09:01 | 88-102 | VGLMWLSYFIASFRL | 676 |
|  | LSYFIASFR | HLA-DRB1*11:01 | 88-102 | VGLMWLSYFIASFRL | 741 |
|  | LSYFIASFR | HLA-DRB1*09:01 | 89-103 | GLMWLSYFIASFRLF | 749 |
|  | LSYFIASFR | HLA-DQA1*05:01/DQB1*03:01 | 89-103 | GLMWLSYFIASFRLF | 767 |
|  | LSYFIASFR | HLA-DRB4*01:01 | 89-103 | GLMWLSYFIASFRLF | 1068 |
|  | LSYFIASFR | HLA-DRB4*01:01 | 90-104 | LMWLSYFIASFRLFA | 1169 |
|  | LSYFIASFR | HLA-DRB1*03:01 | 89-103 | GLMWLSYFIASFRLF | 2621 |
|  | LTWICLLQF | HLA-DRB1*04:04 | 24-38 | IGFLFLTWICLLQFA | 61 |
|  | LTWICLLQF | HLA-DRB1*04:04 | 26-40 | FLFLTWICLLQFAYA | 75 |
|  | LTWICLLQF | HLA-DRB1*04:04 | 25-39 | GFLFLTWICLLQFAY | 79 |
|  | LTWICLLQF | HLA-DRB1*04:04 | 27-41 | LFLTWICLLQFAYAN | 123 |
|  | LTWICLLQF | HLA-DPA1*01/DPB1*04:01 | 26-40 | FLFLTWICLLQFAYA | 169 |
|  | LTWICLLQF | HLA-DRB4*01:01 | 26-40 | FLFLTWICLLQFAYA | 186 |
|  | LTWICLLQF | HLA-DRB1*04:04 | 28-42 | FLTWICLLQFAYANR | 203 |
|  | LTWICLLQF | HLA-DPA1*01/DPB1*04:01 | 27-41 | LFLTWICLLQFAYAN | 207 |
|  | LTWICLLQF | HLA-DRB1*07:01 | 29-43 | LTWICLLQFAYANRN | 252 |
|  | LTWICLLQF | HLA-DRB1*07:01 | 25-39 | GFLFLTWICLLQFAY | 256 |
|  | LTWICLLQF | HLA-DRB1*07:01 | 26-40 | FLFLTWICLLQFAYA | 271 |
|  | LTWICLLQF | HLA-DQA1*01:02/DQB1*06:02 | 26-40 | FLFLTWICLLQFAYA | 283 |
|  | LTWICLLQF | HLA-DQA1*01:02/DQB1*06:02 | 27-41 | LFLTWICLLQFAYAN | 297 |
|  | LTWICLLQF | HLA-DRB4*01:01 | 24-38 | IGFLFLTWICLLQFA | 321 |
|  | LTWICLLQF | HLA-DPA1*03:01/DPB1*04:02 | 28-42 | FLTWICLLQFAYANR | 343 |
|  | LTWICLLQF | HLA-DQA1*01:02/DQB1*06:02 | 24-38 | IGFLFLTWICLLQFA | 360 |
|  | LTWICLLQF | HLA-DRB1*04:05 | 26-40 | FLFLTWICLLQFAYA | 374 |
|  | LTWICLLQF | HLA-DRB1*04:05 | 25-39 | GFLFLTWICLLQFAY | 377 |
|  | LTWICLLQF | HLA-DRB1*04:05 | 24-38 | IGFLFLTWICLLQFA | 382 |
|  | LTWICLLQF | HLA-DRB4*01:01 | 25-39 | GFLFLTWICLLQFAY | 394 |
|  | LTWICLLQF | HLA-DQA1*01:02/DQB1*06:02 | 25-39 | GFLFLTWICLLQFAY | 397 |
|  | LTWICLLQF | HLA-DRB1*04:05 | 27-41 | LFLTWICLLQFAYAN | 408 |
|  | LTWICLLQF | HLA-DPA1*03:01/DPB1*04:02 | 29-43 | LTWICLLQFAYANRN | 412 |
|  | LTWICLLQF | HLA-DRB1*07:01 | 27-41 | LFLTWICLLQFAYAN | 438 |
|  | LTWICLLQF | HLA-DQA1*05:01/DQB1*02:01 | 25-39 | GFLFLTWICLLQFAY | 617 |
|  | LTWICLLQF | HLA-DQA1*01:01/DQB1*05:01 | 24-38 | IGFLFLTWICLLQFA | 667 |
|  | LTWICLLQF | HLA-DQA1*01:01/DQB1*05:01 | 25-39 | GFLFLTWICLLQFAY | 691 |
|  | LTWICLLQF | HLA-DPA1*01/DPB1*04:01 | 28-42 | FLTWICLLQFAYANR | 707 |
|  | LTWICLLQF | HLA-DPA1*01/DPB1*04:01 | 29-43 | LTWICLLQFAYANRN | 759 |
|  | LTWICLLQF | HLA-DQA1*01:01/DQB1*05:01 | 26-40 | FLFLTWICLLQFAYA | 842 |
|  | LTWICLLQF | HLA-DRB1*09:01 | 26-40 | FLFLTWICLLQFAYA | 846 |
|  | LTWICLLQF | HLA-DRB1*09:01 | 27-41 | LFLTWICLLQFAYAN | 872 |
|  | LTWICLLQF | HLA-DQA1*05:01/DQB1*02:01 | 24-38 | IGFLFLTWICLLQFA | 909 |
|  | LTWICLLQF | HLA-DRB1*09:01 | 24-38 | IGFLFLTWICLLQFA | 1050 |
|  | LTWICLLQF | HLA-DQA1*01:01/DQB1*05:01 | 27-41 | LFLTWICLLQFAYAN | 1132 |
|  | LTWICLLQF | HLA-DRB1*04:01 | 27-41 | LFLTWICLLQFAYAN | 1202 |
|  | LTWICLLQF | HLA-DRB1*09:01 | 25-39 | GFLFLTWICLLQFAY | 1203 |
|  | LTWICLLQF | HLA-DRB1*07:01 | 28-42 | FLTWICLLQFAYANR | 1368 |
|  | LTWICLLQF | HLA-DQA1*01:01/DQB1*05:01 | 28-42 | FLTWICLLQFAYANR | 1771 |
|  | LTWICLLQF | HLA-DRB3*01:01 | 27-41 | LFLTWICLLQFAYAN | 2656 |
|  | LTWICLLQF | HLA-DRB3*01:01 | 26-40 | FLFLTWICLLQFAYA | 2682 |
|  | LTWICLLQF | HLA-DRB1*08:02 | 26-40 | FLFLTWICLLQFAYA | 2801 |
|  | LWLLWPVTL | HLA-DRB1*01:01 | 52-66 | IFLWLLWPVTLACFV | 41 |
|  | LWLLWPVTL | HLA-DRB1*04:04 | 52-66 | IFLWLLWPVTLACFV | 42 |
|  | LWLLWPVTL | HLA-DRB1*04:04 | 54-68 | LWLLWPVTLACFVLA | 140 |
|  | LWLLWPVTL | HLA-DRB1*01:01 | 54-68 | LWLLWPVTLACFVLA | 141 |
|  | LWLLWPVTL | HLA-DRB1*15:01 | 52-66 | IFLWLLWPVTLACFV | 228 |
|  | LWLLWPVTL | HLA-DRB1*07:01 | 49-63 | IKLIFLWLLWPVTLA | 243 |
|  | LWLLWPVTL | HLA-DRB1*07:01 | 51-65 | LIFLWLLWPVTLACF | 297 |
|  | LWLLWPVTL | HLA-DRB1*07:01 | 52-66 | IFLWLLWPVTLACFV | 297 |
|  | LWLLWPVTL | HLA-DRB1*09:01 | 49-63 | IKLIFLWLLWPVTLA | 336 |
|  | LWLLWPVTL | HLA-DRB1*07:01 | 50-64 | KLIFLWLLWPVTLAC | 338 |
|  | LWLLWPVTL | HLA-DRB1*09:01 | 51-65 | LIFLWLLWPVTLACF | 364 |
|  | LWLLWPVTL | HLA-DRB1*09:01 | 50-64 | KLIFLWLLWPVTLAC | 370 |
|  | LWLLWPVTL | HLA-DRB1*09:01 | 52-66 | IFLWLLWPVTLACFV | 422 |
|  | LWLLWPVTL | HLA-DRB1*07:01 | 53-67 | FLWLLWPVTLACFVL | 491 |
|  | LWLLWPVTL | HLA-DRB1*07:01 | 54-68 | LWLLWPVTLACFVLA | 502 |
|  | LWLLWPVTL | HLA-DRB1*11:01 | 52-66 | IFLWLLWPVTLACFV | 520 |
|  | LWLLWPVTL | HLA-DRB1*15:01 | 54-68 | LWLLWPVTLACFVLA | 689 |
|  | LWLLWPVTL | HLA-DRB5*01:01 | 52-66 | IFLWLLWPVTLACFV | 713 |
|  | LWLLWPVTL | HLA-DRB4*01:01 | 51-65 | LIFLWLLWPVTLACF | 759 |
|  | LWLLWPVTL | HLA-DRB1*09:01 | 53-67 | FLWLLWPVTLACFVL | 773 |
|  | LWLLWPVTL | HLA-DRB1*09:01 | 54-68 | LWLLWPVTLACFVLA | 855 |
|  | LWLLWPVTL | HLA-DRB4*01:01 | 52-66 | IFLWLLWPVTLACFV | 904 |
|  | LWLLWPVTL | HLA-DRB1*08:02 | 51-65 | LIFLWLLWPVTLACF | 925 |
|  | LWLLWPVTL | HLA-DRB1*13:02 | 49-63 | IKLIFLWLLWPVTLA | 1325 |
|  | LWLLWPVTL | HLA-DRB1*13:02 | 50-64 | KLIFLWLLWPVTLAC | 1340 |
|  | LWLLWPVTL | HLA-DRB1*13:02 | 51-65 | LIFLWLLWPVTLACF | 1381 |
|  | LWLLWPVTL | HLA-DRB1*13:02 | 52-66 | IFLWLLWPVTLACFV | 1395 |
|  | LWLLWPVTL | HLA-DRB1*11:01 | 54-68 | LWLLWPVTLACFVLA | 1878 |
|  | LWLLWPVTL | HLA-DRB1*04:05 | 54-68 | LWLLWPVTLACFVLA | 2032 |
|  | LWLLWPVTL | HLA-DRB5*01:01 | 54-68 | LWLLWPVTLACFVLA | 2126 |
|  | LWLLWPVTL | HLA-DRB1*08:02 | 49-63 | IKLIFLWLLWPVTLA | 2164 |
|  | LWLLWPVTL | HLA-DRB1*08:02 | 50-64 | KLIFLWLLWPVTLAC | 2628 |
|  | LWPVTLACF | HLA-DQA1*01:02/DQB1*06:02 | 55-69 | WLLWPVTLACFVLAA | 220 |
|  | LWPVTLACF | HLA-DQA1*01:02/DQB1*06:02 | 54-68 | LWLLWPVTLACFVLA | 463 |
|  | LWPVTLACF | HLA-DRB1*08:02 | 52-66 | IFLWLLWPVTLACFV | 637 |
|  | LWPVTLACF | HLA-DRB1*08:02 | 53-67 | FLWLLWPVTLACFVL | 698 |
|  | LWPVTLACF | HLA-DQA1*01:02/DQB1*06:02 | 53-67 | FLWLLWPVTLACFVL | 738 |
|  | LWPVTLACF | HLA-DRB1*08:02 | 54-68 | LWLLWPVTLACFVLA | 743 |
|  | LWPVTLACF | HLA-DQA1*01:02/DQB1*06:02 | 52-66 | IFLWLLWPVTLACFV | 749 |
|  | LWPVTLACF | HLA-DRB1*08:02 | 55-69 | WLLWPVTLACFVLAA | 764 |
|  | LWPVTLACF | HLA-DRB1*12:01 | 54-68 | LWLLWPVTLACFVLA | 976 |
|  | LWPVTLACF | HLA-DRB1*08:02 | 56-70 | LLWPVTLACFVLAAV | 1065 |
|  | LWPVTLACF | HLA-DRB1*08:02 | 57-71 | LWPVTLACFVLAAVY | 1240 |
|  | LWPVTLACF | HLA-DQA1*03:01/DQB1*03:02 | 55-69 | WLLWPVTLACFVLAA | 1266 |
|  | LWPVTLACF | HLA-DQA1*05:01/DQB1*03:01 | 54-68 | LWLLWPVTLACFVLA | 1412 |
|  | LWPVTLACF | HLA-DRB4*01:01 | 53-67 | FLWLLWPVTLACFVL | 1561 |
|  | LWPVTLACF | HLA-DRB4*01:01 | 54-68 | LWLLWPVTLACFVLA | 1850 |
|  | LWPVTLACF | HLA-DQA1*03:01/DQB1*03:02 | 52-66 | IFLWLLWPVTLACFV | 2376 |
|  | LWPVTLACF | HLA-DRB4*01:01 | 55-69 | WLLWPVTLACFVLAA | 2398 |
|  | LWPVTLACF | HLA-DQA1*03:01/DQB1*03:02 | 53-67 | FLWLLWPVTLACFVL | 2407 |
|  | LWPVTLACF | HLA-DQA1*03:01/DQB1*03:02 | 54-68 | LWLLWPVTLACFVLA | 2636 |
|  | LYIIKLIFL | HLA-DPA1*02:01/DPB1*01:01 | 42-56 | RNRFLYIIKLIFLWL | 24 |
|  | LYIIKLIFL | HLA-DPA1*03:01/DPB1*04:02 | 42-56 | RNRFLYIIKLIFLWL | 30 |
|  | LYIIKLIFL | HLA-DRB4*01:01 | 43-57 | NRFLYIIKLIFLWLL | 31 |
|  | LYIIKLIFL | HLA-DRB1*15:01 | 43-57 | NRFLYIIKLIFLWLL | 37 |
|  | LYIIKLIFL | HLA-DRB1*01:01 | 43-57 | NRFLYIIKLIFLWLL | 41 |
|  | LYIIKLIFL | HLA-DPA1*02:01/DPB1*01:01 | 41-55 | NRNRFLYIIKLIFLW | 45 |
|  | LYIIKLIFL | HLA-DRB1*01:01 | 44-58 | RFLYIIKLIFLWLLW | 49 |
|  | LYIIKLIFL | HLA-DRB1*01:01 | 42-56 | RNRFLYIIKLIFLWL | 56 |
|  | LYIIKLIFL | HLA-DRB1*15:01 | 42-56 | RNRFLYIIKLIFLWL | 63 |
|  | LYIIKLIFL | HLA-DRB1*01:01 | 41-55 | NRNRFLYIIKLIFLW | 64 |
|  | LYIIKLIFL | HLA-DPA1*03:01/DPB1*04:02 | 41-55 | NRNRFLYIIKLIFLW | 64 |
|  | LYIIKLIFL | HLA-DRB1*15:01 | 41-55 | NRNRFLYIIKLIFLW | 69 |
|  | LYIIKLIFL | HLA-DRB4*01:01 | 42-56 | RNRFLYIIKLIFLWL | 98 |
|  | LYIIKLIFL | HLA-DRB4*01:01 | 41-55 | NRNRFLYIIKLIFLW | 118 |
|  | LYIIKLIFL | HLA-DPA1*01/DPB1*04:01 | 42-56 | RNRFLYIIKLIFLWL | 151 |
|  | LYIIKLIFL | HLA-DPA1*02:01/DPB1*05:01 | 41-55 | NRNRFLYIIKLIFLW | 155 |
|  | LYIIKLIFL | HLA-DPA1*01/DPB1*04:01 | 41-55 | NRNRFLYIIKLIFLW | 404 |
|  | LYIIKLIFL | HLA-DRB1*11:01 | 46-60 | LYIIKLIFLWLLWPV | 427 |
|  | LYIIKLIFL | HLA-DRB1*08:02 | 43-57 | NRFLYIIKLIFLWLL | 618 |
|  | LYIIKLIFL | HLA-DRB1*08:02 | 42-56 | RNRFLYIIKLIFLWL | 697 |
|  | LYIIKLIFL | HLA-DRB1*08:02 | 44-58 | RFLYIIKLIFLWLLW | 734 |
|  | LYIIKLIFL | HLA-DQA1*05:01/DQB1*02:01 | 44-58 | RFLYIIKLIFLWLLW | 823 |
|  | LYIIKLIFL | HLA-DRB1*08:02 | 41-55 | NRNRFLYIIKLIFLW | 849 |
|  | LYIIKLIFL | HLA-DQA1*01:01/DQB1*05:01 | 44-58 | RFLYIIKLIFLWLLW | 1172 |
|  | LYIIKLIFL | HLA-DQA1*01:01/DQB1*05:01 | 43-57 | NRFLYIIKLIFLWLL | 1299 |
|  | LYIIKLIFL | HLA-DQA1*01:01/DQB1*05:01 | 41-55 | NRNRFLYIIKLIFLW | 1548 |
|  | LYIIKLIFL | HLA-DQA1*01:01/DQB1*05:01 | 42-56 | RNRFLYIIKLIFLWL | 1586 |
|  | NRFLYIIKL | HLA-DRB1*01:01 | 39-53 | YANRNRFLYIIKLIF | 105 |
|  | NRFLYIIKL | HLA-DRB1*04:04 | 39-53 | YANRNRFLYIIKLIF | 131 |
|  | NRFLYIIKL | HLA-DPA1*03:01/DPB1*04:02 | 39-53 | YANRNRFLYIIKLIF | 167 |
|  | NRFLYIIKL | HLA-DRB4*01:01 | 39-53 | YANRNRFLYIIKLIF | 271 |
|  | NRFLYIIKL | HLA-DRB1*15:01 | 39-53 | YANRNRFLYIIKLIF | 275 |
|  | NRFLYIIKL | HLA-DRB4*01:01 | 38-52 | AYANRNRFLYIIKLI | 427 |
|  | NRFLYIIKL | HLA-DRB1*15:01 | 38-52 | AYANRNRFLYIIKLI | 643 |
|  | NRFLYIIKL | HLA-DRB1*01:01 | 38-52 | AYANRNRFLYIIKLI | 717 |
|  | NRFLYIIKL | HLA-DQA1*01:02/DQB1*06:02 | 38-52 | AYANRNRFLYIIKLI | 769 |
|  | NRFLYIIKL | HLA-DQA1*01:02/DQB1*06:02 | 39-53 | YANRNRFLYIIKLIF | 786 |
|  | NRFLYIIKL | HLA-DQA1*01:02/DQB1*06:02 | 40-54 | ANRNRFLYIIKLIFL | 789 |
|  | NRFLYIIKL | HLA-DQA1*01:02/DQB1*06:02 | 41-55 | NRNRFLYIIKLIFLW | 809 |
|  | NRFLYIIKL | HLA-DQA1*01:02/DQB1*06:02 | 43-57 | NRFLYIIKLIFLWLL | 1128 |
|  | NRFLYIIKL | HLA-DRB1*11:01 | 38-52 | AYANRNRFLYIIKLI | 1158 |
|  | NRFLYIIKL | HLA-DRB1*09:01 | 39-53 | YANRNRFLYIIKLIF | 1327 |
|  | NRFLYIIKL | HLA-DQA1*01:02/DQB1*06:02 | 42-56 | RNRFLYIIKLIFLWL | 1484 |
|  | NRFLYIIKL | HLA-DQA1*01:01/DQB1*05:01 | 40-54 | ANRNRFLYIIKLIFL | 1489 |
|  | NRFLYIIKL | HLA-DQA1*01:01/DQB1*05:01 | 39-53 | YANRNRFLYIIKLIF | 1947 |
|  | NRFLYIIKL | HLA-DQA1*01:01/DQB1*05:01 | 38-52 | AYANRNRFLYIIKLI | 2453 |
|  | NRFLYIIKL | HLA-DRB1*07:01 | 38-52 | AYANRNRFLYIIKLI | 2508 |
|  | RFLYIIKLI | HLA-DPA1*02:01/DPB1*01:01 | 39-53 | YANRNRFLYIIKLIF | 125 |
|  | RFLYIIKLI | HLA-DPA1*02:01/DPB1*05:01 | 39-53 | YANRNRFLYIIKLIF | 369 |
|  | RFLYIIKLI | HLA-DRB1*12:01 | 39-53 | YANRNRFLYIIKLIF | 505 |
|  | RFLYIIKLI | HLA-DRB1*04:01 | 39-53 | YANRNRFLYIIKLIF | 586 |
|  | RFLYIIKLI | HLA-DRB3*01:01 | 39-53 | YANRNRFLYIIKLIF | 898 |
|  | RFLYIIKLI | HLA-DQA1*05:01/DQB1*02:01 | 39-53 | YANRNRFLYIIKLIF | 2105 |
|  | RLFARTRSM | HLA-DRB1*07:01 | 97-111 | IASFRLFARTRSMWS | 95 |
|  | RLFARTRSM | HLA-DQA1*05:01/DQB1*03:01 | 96-110 | FIASFRLFARTRSMW | 1364 |
|  | RLFARTRSM | HLA-DQA1*05:01/DQB1*03:01 | 97-111 | IASFRLFARTRSMWS | 1451 |
|  | SSDNIALLV | HLA-DRB1*13:02 | 208-222 | TDHSSSSDNIALLVQ | 518 |
|  | SSDNIALLV | HLA-DRB1*01:01 | 208-222 | TDHSSSSDNIALLVQ | 1840 |
|  | SSDNIALLV | HLA-DQA1*03:01/DQB1*03:02 | 208-222 | TDHSSSSDNIALLVQ | 2065 |
|  | SYFIASFRL | HLA-DRB1*01:01 | 90-104 | LMWLSYFIASFRLFA | 61 |
|  | SYFIASFRL | HLA-DRB1*01:01 | 91-105 | MWLSYFIASFRLFAR | 65 |
|  | SYFIASFRL | HLA-DRB1*15:01 | 90-104 | LMWLSYFIASFRLFA | 68 |
|  | SYFIASFRL | HLA-DRB1*01:01 | 89-103 | GLMWLSYFIASFRLF | 69 |
|  | SYFIASFRL | HLA-DRB1*01:01 | 92-106 | WLSYFIASFRLFART | 71 |
|  | SYFIASFRL | HLA-DRB1*15:01 | 89-103 | GLMWLSYFIASFRLF | 72 |
|  | SYFIASFRL | HLA-DRB1*15:01 | 91-105 | MWLSYFIASFRLFAR | 72 |
|  | SYFIASFRL | HLA-DRB1*15:01 | 92-106 | WLSYFIASFRLFART | 92 |
|  | SYFIASFRL | HLA-DRB1*07:01 | 90-104 | LMWLSYFIASFRLFA | 114 |
|  | SYFIASFRL | HLA-DRB1*07:01 | 91-105 | MWLSYFIASFRLFAR | 116 |
|  | SYFIASFRL | HLA-DRB1*07:01 | 92-106 | WLSYFIASFRLFART | 128 |
|  | SYFIASFRL | HLA-DRB1*07:01 | 89-103 | GLMWLSYFIASFRLF | 133 |
|  | SYFIASFRL | HLA-DRB1*15:01 | 93-107 | LSYFIASFRLFARTR | 162 |
|  | SYFIASFRL | HLA-DRB1*15:01 | 94-108 | SYFIASFRLFARTRS | 180 |
|  | SYFIASFRL | HLA-DRB1*07:01 | 93-107 | LSYFIASFRLFARTR | 223 |
|  | SYFIASFRL | HLA-DRB1*07:01 | 94-108 | SYFIASFRLFARTRS | 245 |
|  | SYFIASFRL | HLA-DQA1*04:01/DQB1*04:02 | 89-103 | GLMWLSYFIASFRLF | 1848 |
|  | SYFIASFRL | HLA-DQA1*04:01/DQB1*04:02 | 90-104 | LMWLSYFIASFRLFA | 2007 |
|  | SYFIASFRL | HLA-DQA1*04:01/DQB1*04:02 | 91-105 | MWLSYFIASFRLFAR | 2376 |
|  | SYFIASFRL | HLA-DRB1*13:02 | 90-104 | LMWLSYFIASFRLFA | 2491 |
|  | SYFIASFRL | HLA-DQA1*04:01/DQB1*04:02 | 92-106 | WLSYFIASFRLFART | 2543 |
|  | YANRNRFLY | HLA-DRB1*11:01 | 39-53 | YANRNRFLYIIKLIF | 11 |
|  | YANRNRFLY | HLA-DRB1*07:01 | 39-53 | YANRNRFLYIIKLIF | 39 |
|  | YANRNRFLY | HLA-DRB5*01:01 | 39-53 | YANRNRFLYIIKLIF | 40 |
|  | YANRNRFLY | HLA-DRB1*04:05 | 39-53 | YANRNRFLYIIKLIF | 59 |
|  | YANRNRFLY | HLA-DPA1*02:01/DPB1*01:01 | 38-52 | AYANRNRFLYIIKLI | 315 |
|  | YANRNRFLY | HLA-DPA1*02:01/DPB1*05:01 | 36-50 | QFAYANRNRFLYIIK | 436 |
|  | YANRNRFLY | HLA-DPA1*02:01/DPB1*05:01 | 37-51 | FAYANRNRFLYIIKL | 449 |
|  | YANRNRFLY | HLA-DPA1*02:01/DPB1*05:01 | 35-49 | LQFAYANRNRFLYII | 452 |
|  | YANRNRFLY | HLA-DPA1*02:01/DPB1*05:01 | 34-48 | LLQFAYANRNRFLYI | 462 |
|  | YANRNRFLY | HLA-DPA1*02:01/DPB1*01:01 | 37-51 | FAYANRNRFLYIIKL | 473 |
|  | YANRNRFLY | HLA-DPA1*02:01/DPB1*01:01 | 34-48 | LLQFAYANRNRFLYI | 505 |
|  | YANRNRFLY | HLA-DPA1*02:01/DPB1*01:01 | 36-50 | QFAYANRNRFLYIIK | 520 |
|  | YANRNRFLY | HLA-DPA1*02:01/DPB1*01:01 | 35-49 | LQFAYANRNRFLYII | 527 |
|  | YANRNRFLY | HLA-DRB1*03:01 | 39-53 | YANRNRFLYIIKLIF | 556 |
|  | YANRNRFLY | HLA-DRB1*12:01 | 34-48 | LLQFAYANRNRFLYI | 1789 |
|  | YIIKLIFLW | HLA-DPA1*02:01/DPB1*05:01 | 42-56 | RNRFLYIIKLIFLWL | 83 |
|  | YIIKLIFLW | HLA-DRB1*12:01 | 43-57 | NRFLYIIKLIFLWLL | 132 |
|  | YIIKLIFLW | HLA-DRB1*12:01 | 44-58 | RFLYIIKLIFLWLLW | 173 |
|  | YIIKLIFLW | HLA-DRB1*12:01 | 45-59 | FLYIIKLIFLWLLWP | 173 |
|  | YIIKLIFLW | HLA-DRB1*11:01 | 47-61 | YIIKLIFLWLLWPVT | 296 |
|  | YIIKLIFLW | HLA-DRB3*01:01 | 44-58 | RFLYIIKLIFLWLLW | 885 |
|  | YIIKLIFLW | HLA-DRB3*01:01 | 45-59 | FLYIIKLIFLWLLWP | 901 |
|  | YIIKLIFLW | HLA-DQA1*05:01/DQB1*02:01 | 45-59 | FLYIIKLIFLWLLWP | 980 |
|  | YSRYRIGNY | HLA-DRB1*04:01 | 193-207 | FAAYSRYRIGNYKLN | 964 |
|  | YSRYRIGNY | HLA-DRB1*04:01 | 194-208 | AAYSRYRIGNYKLNT | 1077 |
|  | YSRYRIGNY | HLA-DRB1*04:01 | 192-206 | GFAAYSRYRIGNYKL | 1377 |
|  | YSRYRIGNY | HLA-DRB1*04:05 | 194-208 | AAYSRYRIGNYKLNT | 1865 |
|  | YSRYRIGNY | HLA-DRB1*04:05 | 193-207 | FAAYSRYRIGNYKLN | 1867 |
|  | YSRYRIGNY | HLA-DRB1*04:05 | 192-206 | GFAAYSRYRIGNYKL | 1893 |
|  | YSRYRIGNY | HLA-DPA1*02:01/DPB1*05:01 | 193-207 | FAAYSRYRIGNYKLN | 2119 |
|  | YSRYRIGNY | HLA-DPA1*02:01/DPB1*05:01 | 194-208 | AAYSRYRIGNYKLNT | 2208 |
|  | YSRYRIGNY | HLA-DRB1*08:02 | 193-207 | FAAYSRYRIGNYKLN | 2651 |
|  | YSRYRIGNY | HLA-DRB1*08:02 | 192-206 | GFAAYSRYRIGNYKL | 2847 |
|  | YSRYRIGNY | HLA-DRB1*08:02 | 194-208 | AAYSRYRIGNYKLNT | 2855 |
|  | YSRYRIGNY | HLA-DPA1*02:01/DPB1*05:01 | 191-205 | SGFAAYSRYRIGNYK | 2900 |
|  | YSRYRIGNY | HLA-DPA1*02:01/DPB1*05:01 | 192-206 | GFAAYSRYRIGNYKL | 2907 |
| **ORF6 protein** | | | | | |
| **No** | **Core sequence** | **MHC II alleles** | **position** | **Peptide** | **IC50 value** |
|  | HLVDFQVTI | HLA-DPA1*03:01/DPB1*04:02 | 1-15 | MFHLVDFQVTIAEIL | 45 |
|  | HLVDFQVTI | HLA-DPA1*02:01/DPB1*01:01 | 1-15 | MFHLVDFQVTIAEIL | 80 |
|  | HLVDFQVTI | HLA-DRB3*01:01 | 1-15 | MFHLVDFQVTIAEIL | 590 |
|  | HLVDFQVTI | HLA-DRB1*03:01 | 1-15 | MFHLVDFQVTIAEIL | 866 |
|  | HLVDFQVTI | HLA-DRB1*13:02 | 1-15 | MFHLVDFQVTIAEIL | 1057 |
|  | KVSIWNLDY | HLA-DRB1*07:01 | 23-37 | KVSIWNLDYIINLII | 14 |
|  | KVSIWNLDY | HLA-DQA1*01:01/DQB1*05:01 | 18-32 | IMRTFKVSIWNLDYI | 740 |
|  | KVSIWNLDY | HLA-DRB1*15:01 | 21-35 | TFKVSIWNLDYIINL | 1052 |
|  | KVSIWNLDY | HLA-DRB1*15:01 | 20-34 | RTFKVSIWNLDYIIN | 1496 |
|  | KVSIWNLDY | HLA-DQA1*05:01/DQB1*02:01 | 18-32 | IMRTFKVSIWNLDYI | 2020 |
|  | KVSIWNLDY | HLA-DRB1*11:01 | 21-35 | TFKVSIWNLDYIINL | 2548 |
|  | LLIIMRTFK | HLA-DRB1*11:01 | 10-24 | TIAEILLIIMRTFKV | 37 |
|  | LLIIMRTFK | HLA-DRB5*01:01 | 10-24 | TIAEILLIIMRTFKV | 77 |
|  | LLIIMRTFK | HLA-DRB1*08:02 | 12-26 | AEILLIIMRTFKVSI | 382 |
|  | LLIIMRTFK | HLA-DRB1*08:02 | 11-25 | IAEILLIIMRTFKVS | 676 |
|  | LLIIMRTFK | HLA-DRB1*08:02 | 10-24 | TIAEILLIIMRTFKV | 692 |
|  | LLIIMRTFK | HLA-DRB1*03:01 | 10-24 | TIAEILLIIMRTFKV | 895 |
|  | HLVDFQVTI | HLA-DPA1*03:01/DPB1*04:02 | 1-15 | MFHLVDFQVTIAEIL | 45 |
| **Nucleocapsid phosphoprotein** | | | | | |
| **No** | **Core sequence** | **MHC II alleles** | **position** | **Peptide** | **IC50 value** |
|  | DLSPRWYFY | HLA-DPA1*01:03/DPB1*02:01 | 98-112 | DGKMKDLSPRWYFYY | 515 |
|  | KKADETQAL | HLA-DRB3*01:01 | 369-383 | KKDKKKKADETQALP | 518 |
|  | KKADETQAL | HLA-DRB3*01:01 | 370-384 | KDKKKKADETQALPQ | 522 |
|  | KKADETQAL | HLA-DRB3*01:01 | 372-386 | KKKKADETQALPQRQ | 525 |
|  | KKADETQAL | HLA-DRB3*01:01 | 371-385 | DKKKKADETQALPQR | 526 |
|  | KKADETQAL | HLA-DQA1*01:02/DQB1*06:02 | 372-386 | KKKKADETQALPQRQ | 1425 |
|  | KKADETQAL | HLA-DRB3*01:01 | 373-387 | KKKADETQALPQRQK | 1587 |
|  | KKADETQAL | HLA-DRB3*01:01 | 374-388 | KKADETQALPQRQKK | 1625 |
|  | KKADETQAL | HLA-DQA1*05:01/DQB1*03:01 | 372-386 | KKKKADETQALPQRQ | 2131 |
|  | KKADETQAL | HLA-DQA1*05:01/DQB1*03:01 | 371-385 | DKKKKADETQALPQR | 2311 |
|  | KKADETQAL | HLA-DPA1*02:01/DPB1*01:01 | 369-383 | KKDKKKKADETQALP | 2443 |
|  | KKADETQAL | HLA-DQA1*05:01/DQB1*03:01 | 370-384 | KDKKKKADETQALPQ | 2485 |
|  | KKADETQAL | HLA-DRB1*13:02 | 371-385 | DKKKKADETQALPQR | 2605 |
|  | KKADETQAL | HLA-DRB1*13:02 | 372-386 | KKKKADETQALPQRQ | 2610 |
|  | KKADETQAL | HLA-DRB1*13:02 | 370-384 | KDKKKKADETQALPQ | 2634 |
|  | KKADETQAL | HLA-DQA1*01:02/DQB1*06:02 | 371-385 | DKKKKADETQALPQR | 2642 |
|  | KKADETQAL | HLA-DQA1*01:02/DQB1*06:02 | 370-384 | KDKKKKADETQALPQ | 2800 |
|  | KKADETQAL | HLA-DRB4*01:01 | 372-386 | KKKKADETQALPQRQ | 2834 |
|  | KKADETQAL | HLA-DQA1*05:01/DQB1*03:01 | 369-383 | KKDKKKKADETQALP | 2898 |
|  | KKADETQAL | HLA-DRB1*07:01 | 370-384 | KDKKKKADETQALPQ | 2901 |
|  | KKADETQAL | HLA-DRB1*01:01 | 372-386 | KKKKADETQALPQRQ | 2907 |
|  | KKADETQAL | HLA-DRB1*13:02 | 369-383 | KKDKKKKADETQALP | 2920 |
|  | LSPRWYFYY | HLA-DPA1*01:03/DPB1*02:01 | 101-115 | MKDLSPRWYFYYLGT | 298 |
|  | LSPRWYFYY | HLA-DPA1*01:03/DPB1*02:01 | 102-116 | KDLSPRWYFYYLGTG | 298 |
|  | LSPRWYFYY | HLA-DPA1*01:03/DPB1*02:01 | 100-114 | KMKDLSPRWYFYYLG | 359 |
|  | LSPRWYFYY | HLA-DPA1*01:03/DPB1*02:01 | 99-113 | GKMKDLSPRWYFYYL | 393 |
|  | LSPRWYFYY | HLA-DPA1*02:01/DPB1*01:01 | 103-117 | DLSPRWYFYYLGTGP | 716 |
|  | LSPRWYFYY | HLA-DPA1*02:01/DPB1*05:01 | 99-113 | GKMKDLSPRWYFYYL | 967 |
|  | LSPRWYFYY | HLA-DPA1*02:01/DPB1*05:01 | 101-115 | MKDLSPRWYFYYLGT | 1001 |
|  | LSPRWYFYY | HLA-DPA1*02:01/DPB1*05:01 | 100-114 | KMKDLSPRWYFYYLG | 1005 |
|  | LSPRWYFYY | HLA-DPA1*02:01/DPB1*05:01 | 102-116 | KDLSPRWYFYYLGTG | 1024 |
|  | LSPRWYFYY | HLA-DPA1*02:01/DPB1*01:01 | 102-116 | KDLSPRWYFYYLGTG | 1118 |
|  | LSPRWYFYY | HLA-DPA1*02:01/DPB1*01:01 | 101-115 | MKDLSPRWYFYYLGT | 1182 |
|  | LSPRWYFYY | HLA-DRB1*15:01 | 102-116 | KDLSPRWYFYYLGTG | 1214 |
|  | LSPRWYFYY | HLA-DPA1*02:01/DPB1*01:01 | 100-114 | KMKDLSPRWYFYYLG | 1344 |
|  | LSPRWYFYY | HLA-DPA1*02:01/DPB1*01:01 | 99-113 | GKMKDLSPRWYFYYL | 1415 |
|  | LSPRWYFYY | HLA-DRB1*15:01 | 101-115 | MKDLSPRWYFYYLGT | 1833 |
|  | LSPRWYFYY | HLA-DPA1*01/DPB1*04:01 | 100-114 | KMKDLSPRWYFYYLG | 2023 |
|  | LSPRWYFYY | HLA-DRB1*12:01 | 99-113 | GKMKDLSPRWYFYYL | 2135 |
|  | LSPRWYFYY | HLA-DPA1*01/DPB1*04:01 | 99-113 | GKMKDLSPRWYFYYL | 2581 |
|  | LSPRWYFYY | HLA-DRB1*12:01 | 100-114 | KMKDLSPRWYFYYLG | 2801 |
|  | LSPRWYFYY | HLA-DRB1*12:01 | 103-117 | DLSPRWYFYYLGTGP | 2992 |
|  | QRNAPRITF | HLA-DQA1*01:02/DQB1*06:02 | 4-18 | NGPQNQRNAPRITFG | 1095 |
|  | QRNAPRITF | HLA-DRB1*11:01 | 5-19 | GPQNQRNAPRITFGG | 1460 |
|  | QRNAPRITF | HLA-DRB1*11:01 | 6-20 | PQNQRNAPRITFGGP | 1482 |
|  | QRNAPRITF | HLA-DRB1*11:01 | 4-18 | NGPQNQRNAPRITFG | 1507 |
|  | QRNAPRITF | HLA-DRB1*11:01 | 7-21 | QNQRNAPRITFGGPS | 1565 |
|  | QRNAPRITF | HLA-DQA1*05:01/DQB1*03:01 | 7-21 | QNQRNAPRITFGGPS | 1659 |
|  | SPRWYFYYL | HLA-DPA1*02:01/DPB1*05:01 | 103-117 | DLSPRWYFYYLGTGP | 1409 |
|  | SPRWYFYYL | HLA-DPA1*02:01/DPB1*05:01 | 104-118 | LSPRWYFYYLGTGPE | 2038 |
|  | SPRWYFYYL | HLA-DQA1*01:01/DQB1*05:01 | 101-115 | MKDLSPRWYFYYLGT | 2051 |
|  | SPRWYFYYL | HLA-DPA1*03:01/DPB1*04:02 | 101-115 | MKDLSPRWYFYYLGT | 2083 |
|  | SPRWYFYYL | HLA-DPA1*02:01/DPB1*05:01 | 105-119 | SPRWYFYYLGTGPEA | 2546 |
|  | SSPDDQIGY | HLA-DRB1*03:01 | 78-92 | SSPDDQIGYYRRATR | 1198 |
|  | SSPDDQIGY | HLA-DRB3*01:01 | 73-87 | PINTNSSPDDQIGYY | 2077 |
|  | SSPDDQIGY | HLA-DRB3*01:01 | 74-88 | INTNSSPDDQIGYYR | 2098 |
|  | SSPDDQIGY | HLA-DRB3*01:01 | 75-89 | NTNSSPDDQIGYYRR | 2167 |
|  | SSPDDQIGY | HLA-DRB3*01:01 | 76-90 | TNSSPDDQIGYYRRA | 2185 |
|  | SSPDDQIGY | HLA-DRB1*04:04 | 78-92 | SSPDDQIGYYRRATR | 2280 |
|  | TWLTYTGAI | HLA-DRB1*07:01 | 324-338 | VTPSGTWLTYTGAIK | 767 |
|  | TWLTYTGAI | HLA-DRB1*13:02 | 325-339 | TPSGTWLTYTGAIKL | 903 |
|  | TWLTYTGAI | HLA-DPA1*01:03/DPB1*02:01 | 325-339 | TPSGTWLTYTGAIKL | 907 |
|  | TWLTYTGAI | HLA-DPA1*01/DPB1*04:01 | 325-339 | TPSGTWLTYTGAIKL | 2189 |
|  | TWLTYTGAI | HLA-DRB1*13:02 | 324-338 | VTPSGTWLTYTGAIK | 2762 |
