# Supplementary material for: Computational perspectives revealed prospective vaccine candidates from five structural proteins of novel SARS corona virus 2019 (SARS-CoV-2)
Source: PeerJ. 2020 Sep 29;8:e9855. doi: 10.7717/peerj.9855 (PMC7531350; doi:10.7717/peerj.9855)
Supplement: Supplemental Information 8 [file peerj-08-9855-s008.docx]

|  | **MHC-1** | | | | | **MHC-2** | |
| --- | --- | --- | --- | --- | --- | --- | --- |
| **PDB id** | **5VGE** | **6J1W** | **5VWH** | **3X13** | **3C9N** | **2FSE** | **5LAX** |
| **HLA molecule** | HLA-C*07:02 | HLA-A*30:01 | HLA-B*58:01 | HLA-B*08:01 | HLA-B*15:01 | HLA-DRB1*01:01 | HLA-DRB1*04:01 |
| **Epitope** | RYRPGTVAL | AIFQSSMTK | LSSPVTKSW | FLRGRAYGL | VQQESSFVM | AGFKGEQGPKGEPG | SKGLFRAAVPSGAS |
| HADDOCK score | -64.1 +/- 8.8 | -65.0 +/- 2.1 | -49.5 +/- 1.8 | -57.2 +/- 1.1 | -29.6 +/- 7.8 | -92.1 +/- 5.8 | -96.7 +/- 5.6 |
| Cluster size | 84 | 95 | 95 | 22 | 44 | 8 | 12 |
| RMSD from the overall  lowest-energy structure | 0.4 +/- 0.3 | 0.5 +/- 0.2 | 0.8 +/- 0.4 | 0.7 +/- 0.4 | 2.8 +/- 0.1 | 0.7 +/- 0.4 | 0.6 +/- 0.4 |
| Van der Waals energy | -70.8 +/- 6.0 | -59.0 +/- 2.4 | -69.5 +/- 6.9 | -61.5 +/- 1.9 | -67.4 +/- 8.6 | -78.9 +/- 4.8 | -85.7 +/- 4.6 |
| Electrostatic energy | -235.4 +/- 7.5 | -292.6 +/- 13.9 | -181.8 +/- 20.3 | -300.9 +/- 13.3 | -194.0 +/- 37.9 | -209.3 +/- 9.7 | -218.4 +/- 8.7 |
| Desolvation energy | -17.1 +/- 0.8 | -29.4 +/- 1.7 | -27.8 +/- 0.8 | -34.2 +/- 1.7 | -11.1 +/- 1.3 | -18.4 +/- 0.4 | -29.5 +/- 1.6 |
| Restraints violation energy | 709.9 +/- 49.4 | 818.9 +/- 48.9 | 841.8 +/- 105.3 | 986.1 +/- 15.5 | 877.0 +/- 93.0 | 470.6 +/- 53.2 | 621.7 +/- 69.4 |
| Buried Surface Area | 1896.3 +/- 27.4 | 1802.8 +/- 36.3 | 1801.8 +/- 24.7 | 1964.0 +/- 25.3 | 1784.7 +/- 95.4 | 2236.2 +/- 18.4 | 2131.9 +/- 17.8 |
| Z-Score | -2.3 | -1.2 | -1.0 | -1.0 | -1.6 | 0.0 | -1.5 |

**Supplementary Table-S6: Results of Re-dcoking and validation study performed using HADDOCK 2.4 tool**
