# Supplementary material for: Computational perspectives revealed prospective vaccine candidates from five structural proteins of novel SARS corona virus 2019 (SARS-CoV-2)
Source: PeerJ. 2020 Sep 29;8:e9855. doi: 10.7717/peerj.9855 (PMC7531350; doi:10.7717/peerj.9855)
Supplement: Supplemental Information 9 [file peerj-08-9855-s009.docx]

**Supplementary Table-S7: (a) Linear B-cell epitopes from SARS-CoV-2 predicted by BepiPred-2.0 (b) Linear B-cell epitopes from SARS-CoV-2 predicted by LBtope**

**(a)**

| **Surface glycoprotein** | | | | | | |
| --- | --- | --- | --- | --- | --- | --- |
| **B-cell epitopes** | **Position** | **Antigenicity**  **score** | **Conservancy** | **Toxicity** | **Allergenicity** | |
|  |  |  |  |  | **AllerTOP**  **2.0** | **AllergenFP**  **1.0** |
| QCVNLTTRTQLPPAYTNSFTRGV | 14-36 | 0.7515 | 26.09% | NT | YES | NO |
| FSNVTWFHAIHVSGTNGTKRFDN | 59-81 | 0.6767 | 39.13% | NT | YES | YES |
| LGVYYHKNNKSWMESEFRVYSSA | 141-163 | 0.4829 | 21.74% | NT | NO | YES |
| DLEGKQGNFKNLRE | 178-191 | 0.9256 | 64.29% | NT | YES | NO |
| HTPINLVRDLPQGFSA | 207-222 | 0.3936 | 56.25% | NT | NO | NO |
| YLTPGDSSSGWTA | 248-260 | 0.627 | 38.46% | NT | NO | NO |
| YQTSNFRVQP | 313-322 | 1.1866 | 90.00% | NT | NO | YES |
| FGEVFNATRFASVYAWNRK | 338-356 | 0.2386 | 84.21% | NT | YES | NO |
| NSASFSTFKCYGVSPTKLNDLCFTNV | 370-395 | 1.3609 | 84.62% | NT | NO | NO |
| GDEVRQIAPGQTGKIADYNYK | 404-424 | 1.3212 | 90.48% | NT | YES | YES |
| NNLDSKVGGNYNY | 439-451 | 0.9437 | 53.85% | NT | YES | NO |
| LFRKSNLKPFERDISTEIYQAGST | 455-478 | 0.129 | 37.50% | NT | YES | YES |
| VEGFNCYFPLQ | 483-493 | 0.5612 | 45.45% | NT | YES | YES |
| ELLHAPATVCGPKKSTNLVK | 516-535 | 0.0205 | 80.00% | NT | YES | NO |
| VNCTEVP | 615-621 | 1.129 | 71.43% | NT | YES | YES |
| ADQLTPTWRVYSTGSNVFQT | 626-645 | 0.3719 | 85.00% | NT | NO | NO |
| VNNSYECDIP | 656-665 | 0.5327 | 80.00% | NT | NO | YES |
| SYQTQTNSPRRARSVASQS | 673-691 | 0.2316 | 26.32% | NT | NO | NO |
| AYTMSLGAENSVAYSN | 694-709 | 0.6003 | 81.25% | NT | YES | NO |
| KQIYKTPPIKDFGGF | 786-800 | -0.3896 | 73.33% | NT | NO | NO |
| LPDPSKPSKR | 806-815 | 0.2641 | 80.00% | NT | YES | NO |
| LADAGFIKQYGDCLGD | 828-843 | 0.0965 | 87.50% | NT | YES | NO |
| VEAEVQI | 987-993 | 0.8205 | 100.00% | NT | YES | YES |
| GQSKRVDFC | 1035-1043 | 1.779 | 100.00% | NT | YES | YES |
| FYEPQIITTD | 1109-1118 | 0.4179 | 80.00% | NT | YES | YES |
| VNNTVYDPLQPELDSFKEELDKYFKNHTSPDVDLGDISGI | 1133-1172 | 0.1613 | 97.50% | NT | NO | NO |
| **orf3a protein** | | | | | | |
| **B-cell epitopes** | **Position** | **Antigenicity**  **score** | **Conservancy** | **Toxicity** | **Allergenicity** | |
|  |  |  |  |  | **AllerTOP**  **2.0** | **AllergenFP**  **1.0** |
| QGEIKDATPSDF | 17-28 | 1.1542 | 33.33% | NT | YES | NO |
| KIITLKKRWQL | 61-71 | 1.0171 | 81.82% | NT | NO | YES |
| GDGTTSPISEHDYQIGGYTEKWESGV | 172-197 | 0.3807 | 57.69% | NT | NO | NO |
| STQLSTDTGV | 216-225 | 0.1281 | 70.00% | NT | NO | YES |
| DEPEEHVQIHTIDGSSGVVNPVMEPIYDEPTTTTS | 238-272 | 0.1815 | 74.29% | NT | YES | YES |
| **Envelope protein** | | | | | | |
| **B-cell epitopes** | **Position** | **Antigenicity**  **score** | **Conservancy** | **Toxicity** | **Allergenicity** | |
|  |  |  |  |  | **AllerTOP**  **2.0** | **AllergenFP**  **1.0** |
| YVYSRVKNLNSSRVP | 57-71 | 0.4492 | 80.00% | NT | NO | NO |
| **membrane glycoprotein** | | | | | | |
| **B-cell epitopes** | **Position** | **Antigenicity**  **score** | **Conservancy** | **Toxicity** | **Allergenicity** | |
|  |  |  |  |  | **AllerTOP**  **2.0** | **AllergenFP**  **1.0** |
| NGTITVEELKKLLEQW | 5 20 | -0.1969 | 93.75% | NT | YES | NO |
| KLGASQRVAGDS | 180-191 | 0.0439 | 83.33% | NT | NO | NO |
| RYRIGNYKLNTDHSSSSDNIA | 198-218 | 0.1635 | 85.71% | NT | NO | YES |
| **orf6 protein** | | | | | | |
| **B-cell epitopes** | **Position** | **Antigenicity**  **score** | **Conservancy** | **Toxicity** | **Allergenicity** | |
|  |  |  |  |  | **AllerTOP**  **2.0** | **AllergenFP**  **1.0** |
| LTENKYSQLDEEQP | 44-57 | 0.5866 | 57.14% | NT | YES | YES |
| **nucleocapsid phosphoprotein** | | | | | | |
| **B-cell epitopes** | **Position** | **Antigenicity**  **score** | **Conservancy** | **Toxicity** | **Allergenicity** | |
|  |  |  |  |  | **AllerTOP**  **2.0** | **AllergenFP**  **1.0** |
| NGPQNQRNAPRITFGGPSDSTGSNQNGERSGARSKQRRPQGLPNN | 4--48 | 0.3644 | 75.56% | NT | NO | YES |
| HGKEDLKFPRGQGVPINTNSSPDDQIGYYRRATRRIRGGDGKMKDLS | 59-105 | 0.5773 | 89.36% | NT | NO | NO |
| AGLPYGAN | 119-126 | 0.217 | 87.50% | NT | NO | YES |
| GALNTPKDHIGTRNPANNAAI | 137-157 | -0.1862 | 90.48% | NT | NO | NO |
| TLPKGFYAEGSRGGSQASSRSSSRSRNSSRNSTPGSSRGTSPARMAGNGGD | 166-216 | 0.5064 | 88.24% | NT | NO | YES |
| LNQLESKMSGKGQQQQGQTVTKKSAAEASKKPRQKRTATK | 227-266 | 0.5387 | 97.50% | NT | NO | NO |
| RRGPEQTQGNFGDQELIRQGTDYK | 276-299 | 0.6277 | 95.83% | NT | NO | YES |
| DAYKTFPPTEPKKDKKKKADETQALPQRQKKQQTVTLLPAADLDDFSKQLQQSMSSADS | 358-416 | 0.3962 | 83.05% | NT | NO | NO |

**(b)**

| **Surface glycoprotein** | | | | | | |
| --- | --- | --- | --- | --- | --- | --- |
| **B-cell epitopes** | **Position** | **Antigenicity**  **score** | **Conservancy** | **Toxicity** | **Allergenicity** | |
|  |  |  |  |  | **AllerTOP**  **2.0** | **AllergenFP**  **1.0** |
| VFLVLLPLVSSQCVN | 03--17 | 0.5954 | 33.33% | NT | NO | NO |
| TQLPPAYTNSFTRGVYYPDKVFRSSVLHS | 22-50 | 0.0864 | 41.38% | NT | NO | YES |
| GWIFGTT | 103-109 | 0.2772 | 71.43% | NT | NO | YES |
| PFLGVYYHKNNKSW | 139-152 | 0.7487 | 28.57% | NT | NO | NO |
| SQPFLMDL | 172-179 | 0.4797 | 50.00% | NT | YES | YES |
| PINLVRDLPQGFSALEPLVDLPIGI | 209-233 | 0.6961 | 60.00% | NT | YES | NO |
| AGAAAYYVGYLQPRT | 260-274 | 0.9134 | 66.67% | NT | NO | NO |
| PLSETKCTLKSFT | 295-307 | 0.6582 | 61.54% | NT | YES | NO |
| CYGVSPTKLN | 379-388 | 1.5759 | 90.00% | NT | YES | NO |
| KVGGNYNYL | 444-452 | 0.5994 | 55.56% | NT | YES | YES |
| TPCNGVEGFNCY | 478-489 | 0.2717 | 41.67% | NT | YES | YES |
| GFQPTNGVGYQPYRVVVLSF | 496-515 | 0.8857 | 80.00% | NT | YES | NO |
| KKSTNLVKNKCV | 528-539 | 0.5949 | 66.67% | TOXIN | YES | NO |
| TLEILDITPC | 581-590 | 1.5604 | 80.00% | NT | YES | NO |
| RARSVASQ | 683-690 | 0.6389 | 37.50% | NT | NO | NO |
| DFGGFNFSQILPDPSKPSKRSFIEDLL | 796-822 | 0.3239 | 88.89% | NT | NO | NO |
| IQDSLSSTASALGK | 934-947 | 0.5193 | 64.29% | NT | YES | NO |
| TAPAICHDGKAHFPRE | 1077-1092 | -0.3097 | 87.50% | NT | YES | NO |
| TVYDPLQP | 1136-1143 | 0.3135 | 100.00% | NT | NO | YES |
| SVVNIQKEIDRLN | 1175-1187 | 0.3254 | 100.00% | NT | NO | NO |
| PVLKGVKLHY | 1263-1272 | 1.4055 | 100.00% | NT | NO | YES |
| **orf3a protein** | | | | | | |
| **B-cell epitopes** | **Position** | **Antigenicity**  **score** | **Conservancy** | **Toxicity** | **Allergenicity** | |
|  |  |  |  |  | **AllerTOP**  **2.0** | **AllergenFP**  **1.0** |
| EIKDATPSDF | 19-28 | 1.5094 | 40.00% | NT | YES | NO |
| WKCRSKNPLL | 131-140 | 1.2111 | 90.00% | TOXIN | YES | NO |
| YNKIVDEPEEHVQ | 233-245 | -0.0929 | 30.77% | NT | YES | NO |
| **Envelope protein** | | | | | | |
| **B-cell epitopes** | **Position** | **Antigenicity**  **score** | **Conservancy** | **Toxicity** | **Allergenicity** | |
|  |  |  |  |  | **AllerTOP**  **2.0** | **AllergenFP**  **1.0** |
| YVYSRVKNLNSSRVP | 57-71 | 0.4492 | 72.22% | NO | NO | NO |
| **membrane glycoprotein** | | | | | | |
| **B-cell epitopes** | **Position** | **Antigenicity**  **score** | **Conservancy** | **Toxicity** | **Allergenicity** | |
|  |  |  |  |  | **AllerTOP**  **2.0** | **AllergenFP**  **1.0** |
| VEELKKLLEQWNL | 10--22 | -0.3707 | 92.31% | NT | YES | NO |
| RLFARTRSMWSFNPETNILLN | 101-121 | 0.3943 | 100.00% | NT | YES | NO |
| ITVATSRTLSYYKLGASQR | 168-186 | 0.7666 | 100.00% | NT | NO | YES |
| SDNIALL | 214-219 | 0.4677 | 85.71% | NT | NO | YES |
| **orf6 protein** | | | | | | |
| **B-cell epitopes** | **Position** | **Antigenicity**  **score** | **Conservancy** | **Toxicity** | **Allergenicity** | |
|  |  |  |  |  | **AllerTOP**  **2.0** | **AllergenFP**  **1.0** |
| FHLVDFQVTI | 02--11 | 1.8174 | 100.00% | NT | YES | NO |
| SKSLTENKYSQLDEEQPME | 41-59 | 0.4682 | 57.89% | NT | NO | NO |
| **nucleocapsid phosphoprotein** | | | | | | |
| **B-cell epitopes** | **Position** | **Antigenicity**  **score** | **Conservancy** | **Toxicity** | **Allergenicity** | |
|  |  |  |  |  | **AllerTOP**  **2.0** | **AllergenFP**  **1.0** |
| DNGPQNQRNAPRITFGGP | 3--20 | 0.4751 | 66.67% | NT | NO | NO |
| GERSGARSKQRRPQGL | 29-45 | 0.5789 | 81.25% | NT | NO | NO |
| DLKFPRGQGVPINTNSSPDDQIGYYRRATRRIRGGDGKMKDLSPRWYFYYL | 63-113 | 0.6372 | 90.20% | NT | NO | YES |
| DPNFKDQV | 343-350 | 1.7958 | 75.00% | NT | YES | YES |
