# Supplementary material for: Computational perspectives revealed prospective vaccine candidates from five structural proteins of novel SARS corona virus 2019 (SARS-CoV-2)
Source: PeerJ. 2020 Sep 29;8:e9855. doi: 10.7717/peerj.9855 (PMC7531350; doi:10.7717/peerj.9855)
Supplement: Supplemental Information 10 [file peerj-08-9855-s010.docx]

Supplementary Table: 8 Predicted conformational B-cell epitopes of the SARS-CoV-2 structural proteins

| **Surface glycoprotein** | | | | |
| --- | --- | --- | --- | --- |
| **Epitope no.** | **Residues and their positions** | **No. of residues** | **Score** | **3D structure** |
| 1 | V705, A706, Y707, S708, N709, N710, S711, I712, T912, N914, K1073, N1074, F1075, T1076, T1077, A1078, P1079, A1080, I1081, C1082, H1083, D1084, G1085, K1086, A1087, H1088, F1089, P1090, R1091, E1092, G1093, V1094, F1095, V1096, S1097, N1098, G1099, T1100, H1101, W1102, F1103, V1104, T1105, Q1106, R1107, F1109, Y1110, E1111, P1112, Q1113, I1114, I1115, T1116, T1117, D1118, N1119, T1120, F1121, V1122, S1123, G1124, N1125, C1126, D1127, V1128, V1129, I1130, G1131, I1132 | 69 | 0.9 | Figure 4(a) |
| 2 | P337, G339, E340, N343, A344, T345, R346, F347, A348, S349, V350, Y351, A352, W353, N354, S399, F400, V401, I402, R403, G404, D405, E406, R408, Q409, I410, G413, Q414, T415, G416, K417, I418, A419, D420, Y421, N422, Y423, K424, P426, S438, N439, N440, L441, D442, S443, K444, V445, G446, G447, N448, Y449, N450, Y451, L452, Y453, R454, L455, F456, R457, K458, S459, N460, L461, K462, P463, F464, E465, R466, D467, I468, S469, T470, E471, I472, Y473, Q474, A475, G476, S477, T478, P479, C480, N481, G482, V483, E484, G485, F486, N487, C488, Y489, F490, P491, L492, Q493, S494, Y495, G496, F497, Q498, P499, T500, N501, G502, V503, G504, Y505, Q506, P507, R509 | 110 | 0.893 | Figure 4(b) |
| 3 | V3, F4, L5, V6, L7, L8, P9, L10, V11, S12, S13, Q14, C15, V16, N17, L18, T19, T20, R21, T22, Q23, A67, I68, H69, V70, S71, G72, T73, N74, G75, T76, K77, R78, F79, D80, K97, S98, I100, S112, N121, N122, A123, T124, N125, F133, Q134, F135, C136, N137, D138, P139, F140, L141, G142, V143, Y144, Y145, H146, K147, N148, N149, K150, S151, W152, M153, E154, S155, E156, F157, R158, V159, Y160, S161, S162, A163, P174, F175, L176, M177, D178, L179, E180, G181, K182, Q183, G184, A243, L244, H245, R246, S247, Y248, L249, T250, P251, G252, D253, S254, S255, S256, G257, W258, T259, A260, G261, A262, A263 | 107 | 0.836 | Figure 4(c) |
| 4 | K558, F559, L560, P561, F562, Q563 | 6 | 0.809 | Figure 4(d) |
| **orf3a protein** | | | | |
| 1 | M1, D2, L3, F4, M5, R6, T268, T269, T270, T271, S272, V273, P274, L275 | 14 | 0.877 | Figure 4(e) |
| 2 | P104, F105, L106, Y107, L108, Y109 | 6 | 0.83 | Figure 4(f) |
| **Membrane Glycoprotein** | | | | |
| 1 | R72, I73, N74 | 3 | 0.911 | Figure (4g) |
| 2 | Q36, F37, A38, Y39, A40, N41, R42, N43 | 8 | 0.903 | Figure 4(h) |
| **Nucleocapsid Phosphoprotein** | | | | |
| 1 | T91, G96, G97, D98, G99, K100, M101, K102, D103 | 9 | 0.885 | Figure 4(i) |
| 2 | Q58, H59, G60, K61, E62, D63, L64, K65, F66, P67, R68, G69, Q70, G71, V72, P73, I74, N75, S78, S79, P80, D81, D82, Q83, G114, T115, G116, P117, E118, A119, G120, L121, P122, Y123, G124, A125, N126, K127, D128, G129, W132, A134, T135, E136, G137, A138, L139, N140, T141, P142, K143, Q160, L161, P162, Q163, G164, T165, T166, L167, P168, K169, G170, F171, Y172, A173 | 65 | 0.836 | Figure 4(j) |
