# Supplementary material for: Computational perspectives revealed prospective vaccine candidates from five structural proteins of novel SARS corona virus 2019 (SARS-CoV-2)
Source: PeerJ. 2020 Sep 29;8:e9855. doi: 10.7717/peerj.9855 (PMC7531350; doi:10.7717/peerj.9855)
Supplement: Supplemental Information 11 — (A) Different physicochemical properties of the SARS-CoV-2 Structural proteins (B) Different physicochemical properties of the most potential epitopes for MHC-I molecule (C) Different physicochemical properties of the most potential epitopes for MHC - II molecule [file peerj-08-9855-s011.docx]

Supplementary Table: S9 (a) Different physicochemical properties of the SARS-CoV-2 Structural proteins (b) Different physicochemical properties of the most potential epitopes for MHC-I molecule (c) Different physicochemical properties of the most potential epitopes for MHC - II molecule

(a)

| **Features** | | **Surface glycoprotein** | **Orf3A Protein** | **Envelope protein** | **Membrane glycoprotein** | **ORF6 protein** | **Nucleocapsid phosphoprotein** |
| --- | --- | --- | --- | --- | --- | --- | --- |
| Number of amino acids | | 1273 | 275 | 75 | 222 | 61 | 419 |
| Molecular weight | | 141178.47 | 31122.94 | 8365.04 | 25146.62 | 7272.54 | 45625.70 |
| Theoretical pI | | 6.24 | 5.55 | 8.57 | 9.51 | 4.60 | 10.07 |
| Total number of negatively charged  residues (Asp + Glu) | | 110 | 24 | 3 | 13 | 9 | 36 |
| Total number of positively charged  residues (Arg + Lys) | | 103 | 17 | 5 | 21 | 5 | 60 |
| Total number of atoms | | 19710 | 4387 | 1213 | 3600 | 1043 | 6351 |
| Extinction coefficients | | 148960 | 58705 | 6085 | 52160 | 8480 | 43890 |
| Estimated half-life (in hours) | mammalian reticulocytes, in vitro | 30 | 30 | 30 | 30 | 30 | 30 |
|  | yeast, in vivo | >20 | >20 | >20 | >20 | >20 | >20 |
|  | Escherichia coli, in vivo | >10 | >10 | >10 | >10 | >10 | >10 |
| Instability index | | 33.01 | 32.96 | 38.68 | 39.14 | 31.16 | 55.09 |
| Aliphatic index | | 84.67 | 103.42 | 144.00 | 120.86 | 130.98 | 52.53 |
| Grand average of hydropathicity (GRAVY) | | -0.079 | 0.275 | 1.128 | 0.446 | 0.233 | -0.971 |

**(b)**

| **Epitope** | | **YQPYRVVVL** | **PYRVVVLSF** | **GVYFASTEK** | **QLTPTWRVY** | **LKKRWQLAL** | **HVTFFIYNK** | **RFLYIIKLI** | **LTWICLLQF** | **KTFPPTEPK** | **SPRWYFYYL** | **TWLTYTGAI** |
| --- | --- | --- | --- | --- | --- | --- | --- | --- | --- | --- | --- | --- |
| **Number of amino acids** | | 9 | 9 | 9 | 9 | 9 | 9 | 9 | 9 | 9 | 9 | 9 |
| **Molecular weight** | | 1136.36 | 1079.31 | 1001.1 | 1163.34 | 1155.45 | 1168.36 | 1178.53 | 1136.42 | 1044.22 | 1294.47 | 1025.17 |
| **Theoretical pI** | | 8.59 | 9.18 | 6 | 8.75 | 11.17 | 8.6 | 9.99 | 5.52 | 8.59 | 8.22 | 5.28 |
| **Total number of negatively charged**  **residues (Asp + Glu)** | | 0 | 0 | 1 | 0 | 0 | 0 | 0 | 0 | 1 | 0 | 0 |
| **Total number of positively charged**  **residues (Arg + Lys)** | | 1 | 1 | 1 | 1 | 3 | 1 | 2 | 0 | 2 | 1 | 0 |
| **Total number of atoms** | | 166 | 159 | 139 | 165 | 176 | 165 | 183 | 165 | 151 | 177 | 145 |
| **Extinction coefficients** | | 2980 | 1490 | 1490 | 6990 | 5500 | 1490 | 1490 | 5500 |  | 9970 | 6990 |
| **Estimated half-life** | **mammalian reticulocytes, in vitro** | 2.8 h | >20 h | 30 h | 0.8 h | 5.5 h | 3.5 h | 1 h | 5.5 h | 1.3 h | 1.9 h | 7.2 h |
|  | **yeast, in vivo** | 10 m | >20h | >20 h | 10 min | 3 min | 10 min | 2 min | 3 min | 3 min | >20 h | >20 h |
|  | **Escherichia coli, in vivo** | 2 m |  | >10 h | >10 h | 2 min | >10 h | 2 min | 2 min | 3 min | >10 h | >10 h |
| **Instability index** | | -9.9 | 21.91 | -16.19 | 99.32 | 39.48 | -9.98 | 41.43 | 108.2 | 162.91 | -22.4 |  |
| **Aliphatic index** | | 140 | 140 | 43.33 | 75.56 | 141.11 | 75.56 | 216.67 | 173.33 | 0 | 43.33 | 97.78 |
| **Grand average of hydropathicity (GRAVY)** | | 0.467 | 1.222 | -0.2 | -0.578 | -0.389 | 0.189 | 1.578 | 1.789 | 1.633 | -0.567 | 0.6 |

**(c)**

| **Epitope** | | YQPYRVVVLSFELLH | QLTPTWRVYSTGSNV | ITLKKRWQLALSKGV | IGFLFLTWICLLQFA |
| --- | --- | --- | --- | --- | --- |
| **Number of amino acids** | | 15 | 15 | 15 | 15 |
| **Molecular weight** | | 1863.19 | 1708.89 | 1741.15 | 1785.22 |
| **Theoretical pI** | | 6.75 | 8.75 | 11.26 | 5.52 |
| **Total number of negatively charged**  **residues (Asp + Glu)** | | 1 | 0 | 0 | 0 |
| **Total number of positively charged**  **residues (Arg + Lys)** | | 1 | 1 | 4 | 0 |
| **Total number of atoms** | | 268 | 238 | 264 | 260 |
| **Extinction coefficients** | | 2980 | 6990 | 5500 | 5500 |
| **Estimated half-life** | **mammalian reticulocytes, in vitro** | 2.8 h | 0.8 h | 20 h | 20 h |
|  | **yeast, in vivo** | 10 min | 10 min | 30 min | 30 min |
|  | **Escherichia coli, in vivo** | 2 min | 1. h | >10 h | >10 h |
| **Instability index** | | 10.90 | -11.37 | 57.93 | 28.86 |
| **Aliphatic index** | | 136 | 64.67 | 130 | 162.67 |
| **Grand average of hydropathicity (GRAVY)** | | 0.473 | -0.480 | -0.040 | 2.093 |
